# Taxonomic classification of genus *Aeromonas* using open reading frame-based binarized structure network analysis

**DOI:** 10.20407/fmj.2023-007

**Published:** 2023-11-29

**Authors:** Aki Sakurai, Masahiro Suzuki, Kengo Hayashi, Yohei Doi

**Affiliations:** 1 Department of Microbiology, Fujita Health University, School of Medicine, Toyoake, Aichi, Japan; 2 Department of Infectious Diseases, Fujita Health University, School of Medicine, Toyoake, Aichi, Japan; 3 Division of Infectious Diseases, University of Pittsburgh School of Medicine, Pittsburgh, Pennsylvania, USA

**Keywords:** *Aeromonas*, Classification, Open Reading Frame, Phylogenetic Inference, Whole-Genome Sequencing

## Abstract

**Objectives:**

Taxonomic assignment based on whole-genome sequencing data facilitates clear demarcation of species within a complex genus. Here, we applied a unique pan-genome phylogenetic method, open reading frame (ORF)-based binarized structure network analysis (OSNA), for taxonomic inference of *Aeromonas* spp., a complex taxonomic group consisting of 30 species.

**Methods:**

Data from 335 publicly available *Aeromonas* genomes, including the reference genomes of 30 species, were used to build a phylogenetic tree using OSNA. In OSNA, whole-genome structures are expressed as binary sequences based on the presence or absence of ORFs, and a tree is generated using neighbor-net, a distance-based method for constructing phylogenetic networks from binary sequences. The tree built by OSNA was compared to that constructed by a core-genome single-nucleotide polymorphism (SNP)-based analysis. Furthermore, the orthologous average nucleotide identity (OrthoANI) values of the sequences that clustered in a single clade in the OSNA-based tree were calculated.

**Results:**

The phylogenetic tree constructed with OSNA successfully delineated the majority of species of the genus *Aeromonas* forming conspecific clades for individual species, which was corroborated by OrthoANI values. Moreover, the OSNA-based phylogenetic tree demonstrated high compositional similarity to the core-genome SNP-based phylogenetic tree, supported by the Fowlkes–Mallows index.

**Conclusions:**

We propose that OSNA is a useful tool in predicting the taxonomic classification of complex bacterial genera.

## Introduction

The genus *Aeromonas* consists of gram-negative, facultative anaerobic bacilli that are ubiquitous in aquatic environments.^1,2^ *Aeromonas* spp. is an important pathogenic microorganism not only for fish and other poikilothermic animals but also for human beings. To date, *Aeromonas* has been linked to numerous human infectious diseases, including skin and soft-tissue infections, bloodstream infections, and gastroenteritis.^2^

Despite its growing clinical significance, the identification of *Aeromonas* spp. to the species level based on biochemical methods has remained challenging.^1,3^ Furthermore, the taxonomy of the genus *Aeromonas* has undergone significant changes over the past two decades with evolving phylogeny and newly discovered species. This has added to the complexity in the nomenclature of this taxonomic group, now consisting of 30 recognized species and 7 subspecies.^4^ Molecular techniques such as DNA–DNA hybridization (DDH) and 16S rRNA gene sequencing have been used for classification, with the former recognized as the gold standard method for species demarcation.^5,6^ Nevertheless, the use of DDH has been limited by its technical complexity and high probability of errors, while 16S rRNA gene sequencing demonstrates poor discriminatory power due to high interspecies similarities within the genus.^7,8^ Multilocus sequence analysis (MLSA) using at least seven concatenated housekeeping genes has been shown to be a useful tool for species demarcation,^9^ although its accuracy can be impaired by recombination events.^10,11^

With recent advances in whole-genome sequencing (WGS) techniques, genome-wide approaches have been increasingly used to elucidate the molecular epidemiology and species boundaries of bacteria. Average nucleotide identity (ANI), which measures nucleotide-level genomic similarity between two genomes based on whole-genome alignment, is one of the tools most frequently used for identifying species in place of DDH,^5,12^ and its improved algorithms, such as Orthologous ANI (OrthoANI) and FastANI, have become available.^13,14^ However, when using ANI, it has remained unclear how best to select reference sequences to assign against a query sequence for pairwise comparison among a wide variety of species.

Core-genome single-nucleotide polymorphism (cg-SNP)-based analysis has been employed in many studies to construct a phylogenetic tree with both query and reference sequences, which allows for visualization of individual clades that are conspecific and predict the taxonomic affiliation of query sequences.^12^ Nevertheless, the inherent limitation of cg-SNP-based analysis is that its accuracy could be affected by the size of the core genomes (i.e., orthologous sequences conserved in all aligned genomes) and by the linkage disequilibrium between SNP markers and casual variants.^15,16^ The first of these becomes more prominent when query sequences include those from genetically distinct species, in which case cg-SNP-based analysis would not provide sufficient resolution.

Here, we aimed to apply a phylogenetic method that uses the structure of pan-genome open reading frames (ORFs) for taxonomic classification of the genus *Aeromonas*.^17^ In this method that we recently developed, a genome is defined as a set of ORFs without reference to their positions or directions, and the structure of a given genome is described as a binary sequence generated from the presence (assigned “1”) or absence (assigned “0”) of each ORF. Subsequently, a phylogenetic network is constructed with “neighbor-net,” a tool to visualize the binary sequences through network analysis.^18,19^ To evaluate whether the phylogenetic network built by this method, termed ORF-based binarized structure network analysis (OSNA), with both query and reference sequences of *Aeromonas* spp. is useful in predicting the taxonomic assignment of a query sequence based on the clustering information, the OrthoANI values between a query sequence and a reference sequence visually falling in a single clade were calculated. Furthermore, the phylogenetic tree constructed with OSNA was compared to that built by cg-SNP-based analysis to assess the compositional similarity of the two trees. We reasoned that validating the resolution of OSNA in *Aeromonas*, a clinically relevant yet taxonomically complex genus, would indicate its potential utility in a broader set of bacterial genera.

## Materials and Methods

### Published genome data of *Aeromonas* spp.

Genome sequencing data of *Aeromonas* strains registered in the National Institutes of Health (NIH) genetic sequence database (GenBank) as of June 20, 2022, were downloaded. There were 829 genomes, consisting of 210 complete genomes and 619 draft genomes, labeled to belong to 30 species that have been validly published under the International Code of Nomenclature of Prokaryotes^20^ (Supplementary Table 1). Genome sequences of type strains were available in 29 species according to the List of Prokaryotic names with Standing in Nomenclature (LPSN) <https://www.bacterio.net/>,^4^ and they were used as reference genomes. For *A.* *rivipollensis*, the genome of a representative strain was used because that of a type strain was not available. For the reference genomes, the highest-quality genomes were selected from available type-strain genomes. The reference genome set representing 30 species (including 7 subspecies in 2 species) is listed in Table 1. Subsequently, 300 genomes were randomly selected from 829 downloaded *Aeromonas* genomes excluding those of type strains, and they were treated as query sequences, as shown in Supplementary Table 2.

### Pan-genome open reading frame-based binarized structure network analysis (OSNA) for phylogeny inference and presumptive species assignment

The complete or draft genome sequences were broken down into ORFs based on annotation data newly added by DFAST-core <https://dfast.ddbj.nig.ac.jp/dfc/distribution/>.^21^ ORFs collected from the genomes were compared with each other using BLASTn with a database built from each genome dataset. ORFs with ≥80% nucleotide sequence identity and ≥ 80% coverage were considered identical. Second, a hypothetical genome containing all ORFs was constructed as a reference, which was similar to the “pan-genome,” the entire set of orthologous and unique genomes present in the studied group. The positions and directions of the ORFs were not considered in the hypothetical genome architecture. Then, structures of the actual genomes targeted in the ORF analysis were compared to the hypothetical genome. ORFs were searched in the actual genomes using BLASTn to obtain binary sequences, expressed as presence (assigned “1”) or absence (assigned “0”) of each ORF (Supplementary Figure 1). The binary sequences were generated using a python script that is available from GitHub <https://github.com/suzukimasahiro/OSNAp.git>. A phylogenetic network was constructed using neighbor-net by inputting the binary sequences into SplitsTree4 software <http://ab.inf.unituebingen.de/software/splitstree4/welcome.html>.^18,19,22^ The pipeline settings of SplitsTree4 were as follows: distances setting, Uncorrected_P, which was equivalent to Hamming distance; networks setting, NeighborNet; and draw setting, EqualAngle. To validate the visual representation of binary sequences based on the neighbor-net, distances between the genomes were estimated with the Dice index, calculated as an index of the distance between each pair of genomes as follows: DSC (A, B)=2|A∩B|/(|A|+|B|).^17^

### Orthologous average nucleotide identity (OrthoANI) calculation for the identification of species

OrthoANI values were calculated between the query genome sequences and the reference genome sequences that clustered together in a single clade in the OSNA-based phylogenetic network, with an OrthoANI cut-off value of 95% used for species delineation.^5,13,23^ Query sequences with OrthoANI values of less than 95% against closely located reference sequences were compared against all reference genomes listed in Table 1.

### Comparison of OSNA-based phylogenetic network and cg-SNP-based phylogenetic tree

Snippy v4.6.0 <https://github.com/tseemann/snippy.git> was used for the construction of a cg-SNP-based phylogenetic tree, with *A. hydrophila* ATCC 7966 (GenBank accession no. CP000462.1) used as a reference. A variant call required a minimum base quality of 13 and read coverage of 10, with allele frequency of 0.9% at the locus. The final set of cg-SNP alignments were fed into RAxML (Randomized Axelerated Maximum Likelihood, version 8.2.11) to build the maximum likelihood phylogenetic tree with 100 bootstrap iterations. The OSNA-based binary sequences were also given to RAxML with the BINGAMMA model and 100 bootstrap iterations. Compositional similarity between the OSNA-based and cg-SNP-based trees was measured using Fowlkes–Mallows index, which quantifies the similarity of clusters obtained through various clustering algorithms.^24^ More specifically, two sample trees were cut at different levels to produce various values of “number of clusters” (=k) for each tree. Then, the FM index value (=Bk), ranging from 0 to 1 (with 1 indicating greater similarity), was measured for every value of k. These values were used to produce a Bk plot, a scatter plot of Bk versus k. The index was computed with the R packages ape, phytools, and dendextend.^25,26^

## Results

### Pan-genome OSNA-based phylogenetic network and its ability to predict taxonomic affiliation at the species level

**Figure 1:**
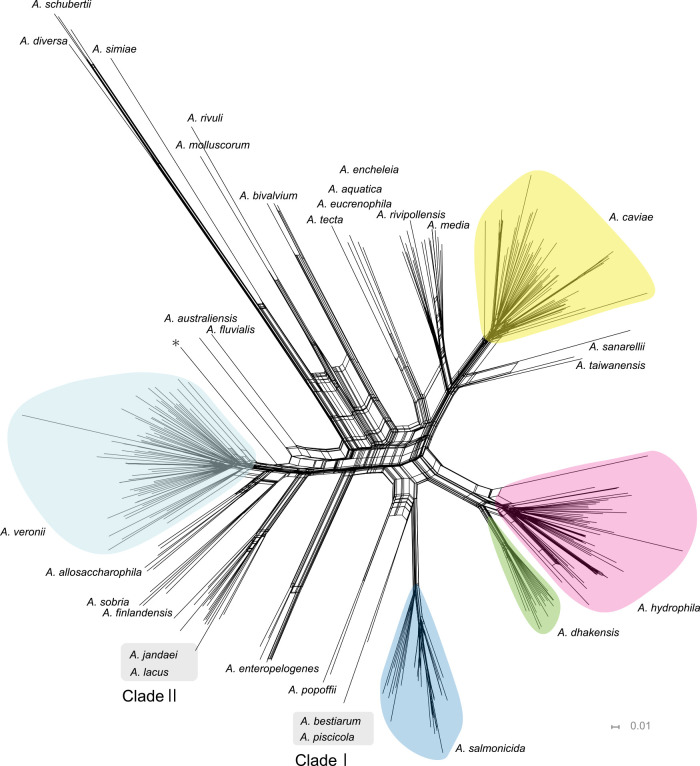
Neighbor-net phylogenetic network constructed by ORF-based binarized structure network analysis (OSNA) Neighbor-net networks were drawn using binary sequences obtained from 335 *Aeromonas* genome sequences, including reference genomes of 30 species. A clade composed of a reference sequence and query sequences with ≥95% OrthoANI values is highlighted in colored irregular circle as a single-species group. The genome with accession no. AGWU01 is shown by an asterisk.

In total, 300 randomly selected query sequences, consisting of 84 complete genomes and 216 draft genomes labeled to belong to 17 *Aeromonas* species, were combined with 35 reference genomes and used to build a phylogenetic tree by OSNA. A total of 49,300 ORFs extracted from 335 *Aeromonas* genomes were used to generate a hypothetical ORF set, representing the pan-genome structure. The total number of bases contained in the 49,300 ORFs was 41,458,401 bp, approximately 9.2 times larger than the average genome size of *Aeromonas* sp. (4,500,680 bp). The phylogenetic network constructed with OSNA successfully delineated the majority of species of the genus *Aeromonas*, as shown in Figure 1. Five prominent clades were formed, with each containing the reference genomes of *A. hydrophila*, *A. veronii*, *A. caviae*, *A. salmonicida*, and *A. dhakensis*, together accounting for 77% of all genomes in this analysis. The clade representing *A. veronii* was the largest and consisted of 78 genomes, followed by *A. caviae* (n=69), *A. hydrophila* (n=57), *A. salmonicida* (n=35), and *A. dhakensis* (n=20). Distinct minor clades were also formed for *A. media* (n=10), *A. rivipollensis* (n=9), *A. allosaccharophila* (n=6), *A. enteropelogenes* (n=5), *A. sobria* (n=4), *A. bivalvium* (n=3), *A. schubertii* (n=3), *A. popoffii* (n=2), *A. sanarellii* (n=2), and *A. encheleia* (n=2). The OrthoANI values between the reference genome and the query genomes that grouped together in the same clade were always greater than 95%, the generally accepted cut-off value for species demarcation, indicating that the topology of the ONSA-based phylogenetic network accurately predicted the taxonomic affiliation of the genus *Aeromonas* at the species level. For *A. australiensis*, *A. aquatica*, *A. eucrenophila*, *A. diversa*, *A. finlandensis*, *A. fluvialis*, *A. molluscorum*, *A. rivuli*, *A. simiae*, *A. taiwanensis*, and *A. tecta*, the reference genome of each species formed an independent terminal node in the tree, reflecting the limitation that the query genome set did not include those belonging to these species.

There were two clades, Clade Ⅰ and Clade Ⅱ shown in Figure 1, which included reference genomes of more than two discrete species and had species boundaries not delineated by the OrthoANI standard cut-off value of 95%. Specifically, Clade Ⅰ consisted of *A. piscicola* and *A. bestiarum*, and the OrthoANI value between their reference genomes was calculated as 95.04%. Similarly, for Clade Ⅱ consisting of *A. jandaei* and *A. lacus*, the OrthoANI value between reference genomes of these species was 95.52%.

Dice indexes between each pair of genomes generated through the analysis are listed in Supplementary Table 3. The intra-species Dice indexes were calculated for the species composed of more than five genomes, including the references. The overall median intra-species Dice index was 0.84 (IQR, 0.82–0.87; range, 0.66–1.0) and the median Dice index of individual species ranged from 0.80 (IQR, 0.79–0.82; range, 0.78–0.83) for *A. allosaccharophila* to 0.89 (IQR, 0.88–0.90; range, 0.86–0.97) for *A. dhakensis*, suggesting different levels of intra-species genetic heterogeneity among *Aeromonas* species.

### Comparison against cg-SNP-based phylogenetic analysis

**Figure 2:**
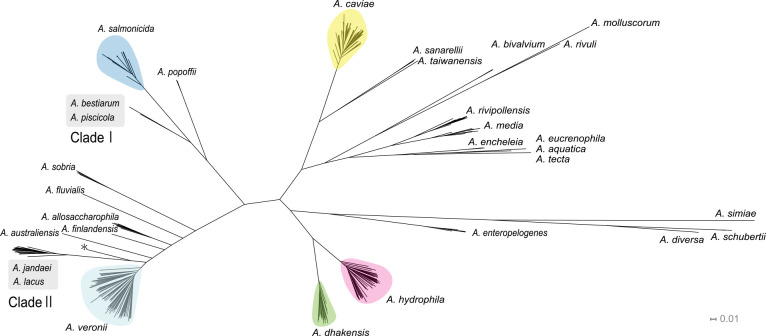
Maximum likelihood phylogenetic tree constructed by core-genome SNP (cg-SNP)-based analysis Maximum likelihood phylogenetic tree was built by cg-SNP-based analysis with 100 bootstrap iterations, using 335 *Aeromonas* genome sequences, including reference genomes of 30 species. A clade composed of a reference sequence and query sequences with ≥95% OrthoANI values is highlighted in colored irregular circle as a single-species group. The genome with accession no. AGWU01 is shown by an asterisk.

**Figure 3:**
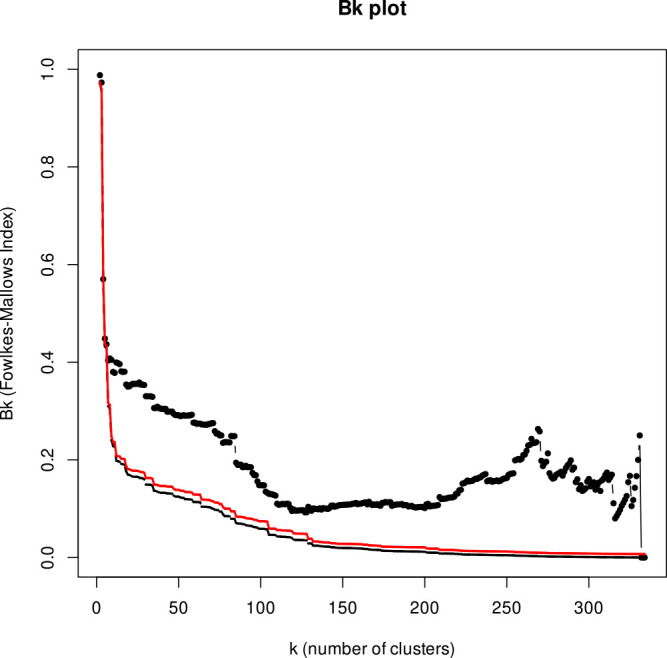
Fowlkes–Mallows index comparing OSNA-based phylogenetic network and cg-SNP-based phylogenetic tree Black line with dots indicates the change of the compositional similarity of clusters (Bk) with the number of clusters (k). Red line illustrates threshold values for rejection of the null hypothesis of non-significant similarity of the clusters’ composition in the dendrograms under comparison. Dashed line shows Bk values under the null hypothesis.

A maximum likelihood phylogenetic tree was constructed by cg-SNP-based analysis, using the same data set comprising 300 query and 35 reference genomes. The size of the core genome used in the cg-SNP analysis was 71,658 bp, which was approximately 1.5% of the total length of the *A. hydrophila* genome (4,744,448 bp in *A. hydrophila* ATCC 7966).^27^ As shown in Figure 2, the cg-SNP-based tree demonstrated congruent topology with the one built with OSNA with overall similarities across all clades. More specifically, query genomes grouped into 15 independent clades, with one representing *A. veronii* being the largest, with 78 genomes, followed by *A. caviae* (n=69), *A. hydrophila* (n=57), *A. salmonicida* (n=35), *A. dhakensis* (n=20), *A. media* (n=10), *A. rivipollensis* (n=9), *A. allosaccharophila* (n=6), *A. enteropelogenes* (n=5), *A. sobria* (n=4), *A. bivalvium* (n=3), *A. schubertii* (n=3), *A. popoffii* (n=2), *A. sanarellii* (n=2), and *A. encheleia* (n=2). The genomes included in individual clades were identical to those constructed by OSNA, with OrthoANI values against the reference genomes greater than 95%. As with OSNA, the reference genomes of *A. australiensis*, *A. aquatica*, *A. eucrenophila*, *A. diversa*, *A. finlandensis*, *A. fluvialis*, *A. molluscorum*, *A. rivuli*, *A. simiae*, *A. taiwanensis*, and *A. tecta* appeared as independent terminal nodes. Furthermore, the reference genomes of *A. piscicola* and *A. bestiarum* grouped together in the cg-SNP-based tree, as well as those of *A. jandaei* and *A. lacus*, shown as Clade Ⅰ and Clade Ⅱ in Figure 2, respectively. These findings were congruent with those obtained by OSNA. Finally, Fowlkes–Mallows (FM) index was calculated to assess cluster similarity of trees generated by OSNA and cg-SNP-based analysis. As shown in Figure 3, the FM index values were higher than those of the red line, the value indicating a critical significance level (i.e., the threshold to reject a null hypothesis that there is no connection between two clusters). This indicates that the topologies of the trees built by OSNA and cg-SNP-based analysis were significantly similar.

### Genome sequences registered with incorrect taxonomic annotation

Of the 300 query genomes downloaded from GenBank, 17 (5.7%) were found to be incorrectly assigned at the species level, as evidenced by both the topology of the phylogenetic trees and the OrthoANI values (Supplementary Table 4). Of these misidentified genomes, 65% (11/17) were re-assigned to *A. rivipollensis* and *A. dhakensis*, which were relatively recently recognized as species in 2016 and 2015, respectively.^28,29^ Notably, there was a genome sequence (Accession no. AGWU01) originally labeled as *A. veronii* that formed an isolated branch outside the clade of *A. veronii* (Figures 1 and 2). It exhibited OrthoANI values of less than 95% against all available reference sequences. This genome might belong to a new species within the genus *Aeromonas*, as implied in a previous study.^30^

## Discussion

With the application of high-throughput sequencing technologies, pan-genome analysis has been used to estimate heritability and genomic relatedness in various organisms.^31^ In this study, phylogenetic analysis using the pan-genome ORF structure successfully delineated the species boundaries of the genus *Aeromonas*, providing phylogenetic resolution comparable to that of the tree built by cg-SNP-based analysis.

Among various phylogenetic methods, a key strength of OSNA is its ability to infer genomic relationships based on its pan-genome data (i.e., the entire set of genes present in a studied group), even if the samples include genetically distant species, whether intentionally or not, with the latter due to bacterial contamination during processing or species misidentification. OSNA was originally developed as a tool to analyze the genetic relatedness of plasmids, for which conserved sequences are limited by the frequent occurrence of homologous recombination and horizontal gene transfer, making SNP-based comparison methods unsuitable.^17^ Because the genomic structure is described as a binary sequence generated from the presence or absence of each ORF with OSNA, the scarcity of a stable core genomic structure shared in a studied group does not affect the phylogenetic resolution of the analysis. Indeed, a genome with little or no genetic relationship to the rest of the group (i.e., a genome with few or no shared ORFs) can be depicted as an “outlier genome” in ONSA. This is because the Hamming distance is used to construct the phylogenetic network from binary sequences, where the number of different characters at the corresponding positions between two strings is computed to estimate genetic distance. Consequently, a genome without shared ORFs, expressed as a series of “0” in the binary sequence, can be joined through “0” in the neighbor-net phylogenetic network. Thus, the genomic relatedness of an outlier genome needs to be carefully evaluated, ideally combined with additional analyses using other methods.

Another strength of OSNA is that it is less affected by sequencing errors acquired during next-generation sequencing (NGS). The error rate by conventional NGS has been reported to range between 0.1% and 1%, depending on the sequencing platform, the GC content of the regions, and the fragment length.^32–34^ These errors are difficult to distinguish from true genetic variations, and thus this could degrade the quality of downstream analysis and potentially mislead phylogenetic inferences of studied genomes, especially when analyzing specific genomic regions with SNP markers. In OSNA, because the genome sequence data are expressed as binary sequences representing pan-genome ORFs, high discriminatory power is maintained irrespective of sequencing errors contained in the reads. These unique features of OSNA would be beneficial when evaluating the genetic relatedness of a group of taxa without sufficient lengths of preserved core genomes, or those including unidentified sequences.

In this study, 5.7% of analyzed *Aeromonas* genomes were incorrectly labeled at the species level in GenBank.^35,36^ Furthermore, there were several species (i.e., *A. piscicola* and *A. bestiarum* in Clade Ⅰ and *A. jandaei* and *A. lacus* in Clade Ⅱ) for which the boundaries were difficult to delineate either by phylogenetic analysis or OrthoANI values.^36^ These results might reflect the prolonged confusion over the complex nomenclature and taxonomy of the genus *Aeromonas* as well as methodological issues, as mentioned above.^2,8^ Accurate species assignment using whole-genome sequencing data would be a prerequisite for better understanding of the epidemiology, pathogenesis, and microbiological and clinical features of individual species.

This study had several limitations. First, the proposed phylogenetic method based on pan-genome ORF structures was not compared to other bioinformatic tools, such as MLSA and other pan-genome approaches, because a cg-SNP-based analysis has been shown by Du et al. to possess sufficient discriminatory power to differentiate *Aeromonas* spp.^37^ Second, we did not evaluate how OSNA performs under conditions where distantly related species from other genera are included in the studied group. This was because the inclusion of genetically remote species in the group was expected to make the comparison between OSNA and cg-SNP-based analysis difficult due to its effect on the length of core genomes and subsequent cg-SNP-based analysis.^15,16^ Third, the phylogenetic resolution of OSNA may be affected by truncated ORFs present at contig ends, which could result in missed prediction of the presence or absence of the ORFs. Finally, we limited our analysis to the genus *Aeromonas* as a proof of concept as we expanded the application of OSNA from plasmids to whole genomes.

In summary, OSNA, a novel phylogenetic network analysis using whole-genome ORF-based binary sequence data, was shown to be useful in predicting the taxonomic assignment of the genus *Aeromonas* using both reference genomes and query genomes. This unique method has the potential for application in other complex taxa where conventional approaches to taxonomy do not provide sufficient resolution to assign species with confidence.

## Data Availability

The genome sequence data presented in this study are publicly available, with their accession numbers listed in Table 1 and Supplementary Tables 1 and 2. The python script used to generate the binary sequences is freely accessible at https://github.com/suzukimasahiro/OSNAp.git. Other data that support the findings of this study are available from the corresponding author upon reasonable request.

## Figures and Tables

**Table1:** Reference genomes representing 30 species and 7 subspecies of the genus *Aeromonas*

*Aeromonas* species	Taxonomy ID	Accession no.	BioSample	BioProject	Strain	Level	Size (Mb)	GC%	Author citation (author(s)-year)
*A. allosaccharophila*	656	NKWZ01	SAMN07312751	PRJNA391781	ATCC 35942^T^	Scaffold	4.5	59	Martinez-Murcia et al. 1992
*A. aquatica*	558964	JRGL01	SAMN03023875	PRJNA260478	AE235^T^	Contig	4.6	61	Beaz-Hidalgo et al. 2015
*A. australiensis*	1114880	CDDH01	SAMEA2752426	PRJEB7021	CECT 8023^T^	Contig	4.1	58	Aravena-Román et al. 2013
*A. bestiarum*	105751	CDDA01	SAMEA2752425	PRJEB7022	CECT 4227^T^	Scaffold	4.7	61	Ali et al. 1996
*A. bivalvium*	440079	CDBT01	SAMEA2752424	PRJEB7023	CECT 7113^T^	Scaffold	4.3	62	Miñana-Galbis et al. 2007
*A. caviae*	648	LS483441.1	SAMEA4475690	PRJEB6403	NCTC12244	Complete	4.6	62	Popoff 1984
*A. dhakensis*	196024	CDBH01	SAMEA2752400	PRJEB7048	CIP 107500^T^	Scaffold	4.7	62	Beaz-Hidalgo et al. 2015
*A. diversa*	502790	CDCE01	SAMEA2752422	PRJEB7026	CECT 4254^T^	Scaffold	4.1	62	Miñana-Galbis et al. 2010
*A. encheleia*	73010	LR134376.1	SAMEA4475689	PRJEB6403	NCTC12917^T^	Complete	4.5	62	Esteve et al. 1995
*A. enteropelogenes*	29489	CDCG01	SAMEA2752420	PRJEB7028	CECT 4487^T^	Scaffold	4.5	60	Schubert et al. 1991
*A. eucrenophila*	649	CDDF01	SAMEA2752419	PRJEB7029	CECT 4224^T^	Scaffold	4.5	61	Schubert and Hegazi 1988
*A. finlandensis*	1543375	JRGK01	SAMN03023686	PRJNA260478	4287D^T^	Contig	4.7	59	Beaz-Hidalgo et al. 2015
*A. fluvialis*	591962	CDBO01	SAMEA2752418	PRJEB7030	CDBO01^T^	Scaffold	3.9	58	Alperi et al. 2010
*A. hydrophila* subsp. *hydrophila*	380703	CP000462.1	SAMN02604052	PRJNA16697	ATCC7966^T^	Complete	4.7	62	Stanier 1943
*A. hydrophila* subsp. *ranae*	208958	CDDC01	SAMEA2752399	PRJEB7049	CIP 107985^T^	Scaffold	4.7	62	Huys et al. 2003
*A. jandaei*	650	CDBV01	SAMEA2752417	PRJEB7031	CECT 4228^T^	Scaffold	4.5	59	Carnahan et al. 1992
*A. lacus*	558884	JRGM01	SAMN03023876	PRJNA260478	AE122^T^	Contig	4.4	59	Beaz-Hidalgo et al. 2015
*A. media*	651	CDBZ01	SAMEA2752416	PRJEB7032	CECT 4232^T^	Scaffold	4.5	61	Allen et al. 1983
*A. molluscorum*	271417	AQGQ01	SAMN02471397	PRJNA183610	848^T^	Contig	4.2	59	Miñana-Galbis et al. 2004
*A. piscicola*	600645	CDBL01	SAMEA2752415	PRJEB7033	LMG24783^T^	Scaffold	5.2	59	Beaz-Hidalgo et al. 2010
*A. popoffii*	70856	CDBI01	SAMEA2752414	PRJEB7034	CIP105493^T^	Scaffold	4.8	59	Huys et al. 1997
*A. rivipollensis*	948519	CP027856.1	SAMN08721782	PRJNA438570	KN-Mc-11N1^T^	Complete	4.5	62	Marti and Balcázar 2016
*A. rivuli*	648794	CDBJ01	SAMEA2752413	PRJEB7035	DSM 22539^T^	Scaffold	4.5	60	Figueras et al. 2011
*A. salmonicida* subsp. *salmonicida*	29491	CP027000.1	SAMN02469939	PRJNA264317	01-B526^T^	Complete	4.7	58	Griffin et al. 1953
*A. salmonicida* subsp. *masoucida*	197700	BAWQ01	SAMD00000014	PRJDB242	NBRC 13784^T^	Contig	4.5	59	Kimura 1969
*A. salmonicida* subsp. *pectinolytica*	96473	NKWI01	SAMN07312770	PRJNA391781	CIP107036^T^	Scaffold	4.8	59	Pavan et al. 2000
*A. salmonicida* subsp. *smithia*	80745	NKWJ01	SAMN07312769	PRJNA391781	CIP104757^T^	Scaffold	4.5	59	Austin et al. 1989
*A. salmonicida* subsp. *achromogenes*	113288	NKWK01	SAMN07312768	PRJNA391781	CIP104001^T^	Scaffold	4.6	59	Schubert 1967
*A. sanarellii*	633415	CDBN01	SAMEA2752411	PRJEB7037	LMG 24682^T^	Scaffold	4.2	63	Alperi et al. 2010
*A. schubertii*	652	CDDB01	SAMEA2752410	PRJEB7038	CECT 4240^T^	Scaffold	4.1	62	Hickman-Brenner et al. 1989
*A. simiae*	218936	CDBY01	SAMEA2752409	PRJEB7039	CIP 107798^T^	Scaffold	4.0	61	Harf-Monteil et al. 2004
*A. sobria*	646	CDBW01	SAMEA2752408	PRJEB7040	CECT 4245^T^	Scaffold	4.7	58	Popoff and Véron 1981
*A. taiwanensis*	633417	CDDD01	SAMEA2752407	PRJEB7041	LMG 24683^T^	Scaffold	4.3	63	Alperi et al. 2010
*A. tecta*	324617	CDCA01	SAMEA2752406	PRJEB7042	CDCA01^T^	Scaffold	4.8	60	Demarta et al. 2010
*A. veronii*	197701	CDDK01	SAMEA2752404	PRJEB7044	CECT4257^T^	Scaffold	4.5	59	Hickman-Brenner et al. 1988
