# Supplementary material for: Taxonomic classification of genus Aeromonas using open reading frame-based binarized structure network analysis
Source: Fujita Med J. 2023 Nov 29;10(1):8–15. doi: 10.20407/fmj.2023-007 (PMC10847635; doi:10.20407/fmj.2023-007)
Supplement: Supplementary file 1 — Supplementary Figure [file fmj-10-008-s001.pdf]

Supplementary Figure 1

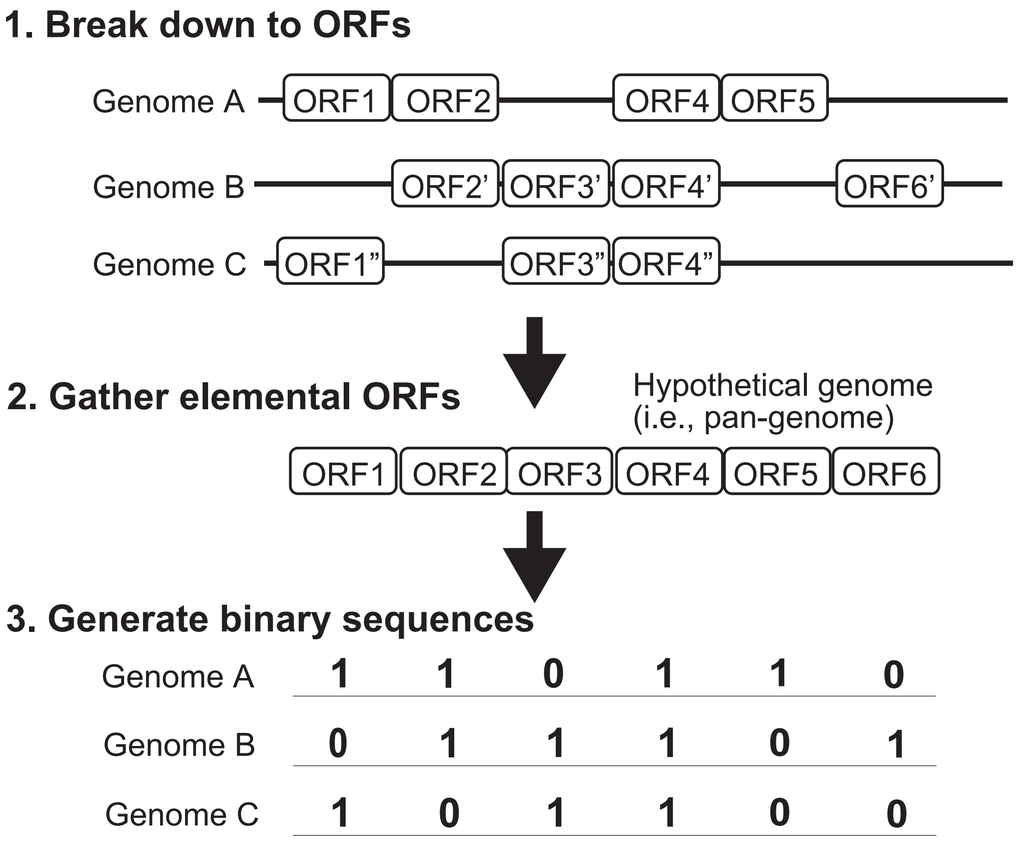

Supplementaty Figure 1. Schematic diagram of binary sequence generation for ORF-based binarized structure network analysis (OSNA)

1. All genome sequences used in this study were broken down into ORFs based on annotation data newly added by DFAST-core. 2. ORFs collected from genomes were compared with each other using BLASTn. ORFs with ≥ 80% nucleotide sequence identity and ≥ 80% cover ratio were considered identical. Subsequently, a hypothetical genome containing all ORFs (i.e., pan-genome) was generated. 3. ORF contents of actual genomes were compared to that of the hypothetical genome. ORFs were searched in the actual genomes using BLASTn to obtain binarized sequences, expressed as presence ("1") or absence ("0") of each ORF.
