# Supplementary material for: Taxonomic classification of genus Aeromonas using open reading frame-based binarized structure network analysis
Source: Fujita Med J. 2023 Nov 29;10(1):8–15. doi: 10.20407/fmj.2023-007 (PMC10847635; doi:10.20407/fmj.2023-007)
Supplement: Supplementary file 2 — Supplementary Tables [file fmj-10-008-s002.pdf]

Supplementary Table 1. List of 829 *Aeromonas* genomes registered in GenBank

Organism Name (GenBank)	Accession.no	BioSample	BioProject	Assembly	Strain	Level	Size(Mb)	GC%	Scaffolds	CDS	Release_Date
A. allosaccharophila	CP065745.1	SAMN13450463	PRJNA231221	GCA_016026615.1	FDAARGOS_933	Complete	4.63	58.8	2	4042	2020-12-14T00:00:00Z
A. australiensis	OU015320.1	SAMEA8533644	PRJEB44171	GCA_907163065.1	Isolate4	Complete	4.56	61.1	1	NA	2021-05-12T00:00:00Z
A. caviae	AP019195.1	SAMD00144878	PRJDB6962	GCA_003925855.2	GSH8M-1	Complete	4.83	60.7	4	4249	2018-11-03T00:00:00Z
A. caviae	AP021927.1	SAMD00194350	PRJDB6962	GCA_014168635.1	WP2-W18-ESBL-01	Complete	4.98	61.0	2	4401	2020-07-21T23:09:00Z
A. caviae	AP022013.1	SAMD00194421	PRJDB6962	GCA_014158455.1	WP3-S18-ESBL-02	Complete	4.86	61.6	1	4342	2020-07-21T23:20:00Z
A. caviae	AP022110.1	SAMD00194514	PRJDB6962	GCA_014169235.1	WP5-W18-ESBL-02	Complete	4.90	61.1	2	4387	2020-07-21T23:34:00Z
A. caviae	AP022214.1	SAMD00194595	PRJDB6962	GCA_014162015.1	WP8-S17-ESBL-03	Complete	4.35	61.7	1	3815	2020-07-21T23:47:00Z
A. caviae	AP022241.1	SAMD00194620	PRJDB6962	GCA_014169675.1	WP8-S18-CRE-01	Complete	4.83	60.7	4	4248	2020-07-21T23:51:00Z
A. caviae	AP022254.1	SAMD00194626	PRJDB6962	GCA_014169735.1	WP8-S18-ESBL-04	Complete	4.57	61.6	2	4019	2020-07-21T23:53:00Z
A. caviae	AP024136.2	SAMD00254948	PRJDB10427	GCA_015508545.2	KAM329	Complete	5.17	60.6	6	4607	2020-10-31T00:00:00Z
A. caviae	AP024402.1	SAMD00270046	PRJDB10427	GCA_016861425.1	KAM376	Complete	4.93	61.3	8	4433	2021-01-20T23:04:00Z
A. caviae	AP024940.1	SAMD00294556	PRJDB11802	GCA_019973835.2	KAM339	Complete	5.01	61.3	8	4525	2021-08-04T00:00:00Z
A. caviae	AP024948.1	SAMD00294561	PRJDB11802	GCA_019972775.2	KAM345	Complete	4.85	61.2	7	4327	2021-08-04T00:00:00Z
A. caviae	AP025280.1	SAMD00424815	PRJDB10427	GCA_021654375.1	NUITM-VA2	Complete	5.04	61.1	1	4469	2021-11-27T00:00:00Z
A. caviae	LS483441.1	SAMEA4475690	PRJEB6403	GCA_900476005.1	NCTC12244	Complete	4.59	61.6	1	4037	2018-06-17T00:00:00Z
A. caviae	CP062787.1	SAMN02934523	PRJNA231221	GCA_000783775.2	FDAARGOS_72	Complete	4.53	61.7	1	3741	2020-10-13T00:00:00Z
A. caviae	CP024198.1	SAMN03389335	PRJNA277314	GCA_000959705.2	8LM	Complete	4.55	61.7	1	3431	2017-10-24T00:00:00Z
A. caviae	CP025777.1	SAMN08290451	PRJNA428427	GCA_003294925.2	R25-2	Complete	5.01	60.7	3	3910	2018-07-09T00:00:00Z
A. caviae	CP025706.1	SAMN08290452	PRJNA428427	GCA_003294895.2	T25-39	Complete	4.97	60.7	3	4133	2018-07-09T00:00:00Z
A. caviae	CP025705.1	SAMN08290453	PRJNA428427	GCA_003294855.2	R25-6	Complete	4.71	61.4	2	4138	2018-07-09T00:00:00Z
A. caviae	CP039832.1	SAMN11521871	PRJNA540105	GCA_007179295.1	WCW1-2	Complete	4.68	61.3	1	4084	2019-07-19T00:00:00Z
A. caviae	CP047981.1	SAMN13871641	PRJNA601658	GCA_013487965.1	NY4617	Complete	4.52	61.6	1	4028	2020-07-23T00:00:00Z
A. caviae	CP047982.1	SAMN13875147	PRJNA601850	GCA_013487985.1	1507-17068	Complete	4.53	61.3	1	3972	2020-07-23T00:00:00Z
A. caviae	CP047983.1	SAMN13875308	PRJNA601853	GCA_013488005.1	1605-27183	Complete	4.86	61.0	1	4255	2020-07-23T00:00:00Z
A. caviae	CP068232.1	SAMN16998432	PRJNA512885	GCA_016728865.1	Aero19	Complete	4.48	61.5	2	3979	2021-01-20T00:00:00Z
A. caviae	CP065937.1	SAMN17014572	PRJNA512885	GCA_016126815.2	Aero21	Complete	5.37	60.5	2	4604	2020-12-20T00:00:00Z
A. caviae	CP066813.1	SAMN17150322	PRJNA512885	GCA_016598815.1	Aero52	Complete	4.53	61.4	4	4016	2021-01-10T00:00:00Z
A. caviae	CP071151.1	SAMN18079944	PRJNA705281	GCA_017280155.1	SS332	Complete	5.01	60.6	3	4171	2021-03-07T00:00:00Z
A. caviae	CP084350.1	SAMN21168050	PRJNA759416	GCA_020405325.1	71442	Complete	4.44	61.7	1	3909	2021-10-10T00:00:00Z
A. caviae	CP085468.1	SAMN21168053	PRJNA759416	GCA_020640975.1	71485	Complete	4.61	61.2	2	4037	2021-10-27T00:00:00Z
A. caviae	CP084031.1	SAMN21545965	PRJNA765169	GCA_020181575.1	K433	Complete	4.68	61.4	1	4166	2021-09-30T00:00:00Z
A. caviae	CP091176.1	SAMN25038853	PRJNA797866	GCA_021609985.1	SCLZS52	Complete	4.94	61.3	9	4419	2022-01-26T00:00:00Z
A. caviae	CP092181.1	SAMN25872065	PRJNA806340	GCA_022343945.1	211703	Complete	4.78	61.8	1	4256	2022-02-20T00:00:00Z
A. dhakensis	CP023141.1	SAMN07577462	PRJNA400818	GCA_002285935.1	KN-Mc-6U21	Complete	4.87	61.5	1	4294	2017-09-05T00:00:00Z
A. dhakensis	CP045311.1	SAMN13030278	PRJNA577584	GCA_022703095.1	Aer_OnlF1	Complete	4.83	61.6	1	4260	2022-03-28T00:00:00Z
A. dhakensis	CP054854.1	SAMN15184665	PRJNA638258	GCA_017310235.1	1706-28330	Complete	4.93	61.5	1	4359	2021-03-09T00:00:00Z
A. dhakensis	CP084351.1	SAMN21168049	PRJNA759416	GCA_020405345.1	71431	Complete	4.77	61.7	1	4190	2021-10-10T00:00:00Z
A. dhakensis	CP084349.1	SAMN21168051	PRJNA759416	GCA_020405305.1	71453	Complete	4.91	61.3	1	4277	2021-10-10T00:00:00Z
A. encheleia	LR134376.1	SAMEA4475689	PRJEB6403	GCA_900637545.1	NCTC12917	Complete	4.54	61.9	1	4027	2018-12-19T00:00:00Z
A. enteropelogenes	CP082887.1	SAMN20888876	PRJNA231221	GCA_019930825.1	FDAARGOS_1459	Complete	4.42	59.9	1	3887	2021-09-12T00:00:00Z
A. enteropelogenes	CP083626.1	SAMN21218865	PRJNA231221	GCA_020097315.1	FDAARGOS_1509	Complete	4.54	59.6	1	4024	2021-09-23T00:00:00Z
A. enteropelogenes	CP083625.1	SAMN21218866	PRJNA231221	GCA_020097295.1	FDAARGOS_1510	Complete	4.39	60.0	1	3852	2021-09-23T00:00:00Z
A. enteropelogenes	CP083612.1	SAMN21402703	PRJNA231221	GCA_020097175.1	FDAARGOS_1536	Complete	4.33	60.0	1	3810	2021-09-23T00:00:00Z
A. hydrophila	AP019193.1	SAMD00144877	PRJDB6962	GCA_004296435.1	GSH8-2	Complete	5.00	61.1	2	4375	2019-02-14T00:00:00Z
A. hydrophila	AP022206.1	SAMD00194576	PRJDB6962	GCA_014161955.1	WP7-S18-ESBL-06	Complete	4.94	61.2	1	4314	2020-07-21T23:45:00Z
A. hydrophila	AP022252.1	SAMD00194624	PRJDB6962	GCA_014169715.1	WP8-S18-ESBL-02	Complete	5.00	61.1	2	4375	2020-07-21T23:52:00Z
A. hydrophila	AP023398.1	SAMD00242552	PRJDB10427	GCA_014679585.1	KAM330	Complete	5.45	60.8	10	4860	2020-08-29T00:00:00Z
A. hydrophila	AP024234.1	SAMD00261012	PRJDB10897	GCA_016592295.1	RIMD111065	Complete	4.96	61.0	1	4331	2020-12-18T00:00:00Z
A. hydrophila	AP025277.1	SAMD00424814	PRJDB10427	GCA_021654355.1	NUITM-VA1	Complete	5.09	61.1	3	4478	2021-11-27T00:00:00Z
A. hydrophila	CP005966.1	SAMN02603614	PRJNA188141	GCA_000401555.1	ML09-119	Complete	5.02	60.8	1	4427	2013-05-24T00:00:00Z
A. hydrophila subsp. *hydrophila*	CP000462.1	SAMN02604052	PRJNA16697	GCA_000014805.1	ATCC 7966	Complete	4.74	61.5	1	4159	2006-11-06T00:00:00Z
A. hydrophila	CP006579.1	SAMN02641590	PRJNA210524	GCA_000512185.1	4AK4	Complete	4.53	62.0	1	3991	2013-12-30T00:00:00Z
A. hydrophila	CP007518.2	SAMN02713488	PRJNA234473	GCA_000612075.2	YL17	Complete	4.80	61.6	1	4186	2014-04-02T00:00:00Z
A. hydrophila	CP007566.1	SAMN02726090	PRJNA227037	GCA_000633175.1	AL09-71	Complete	5.02	60.8	1	4429	2014-04-18T00:00:00Z
A. hydrophila	CP007576.1	SAMN02728499	PRJNA227038	GCA_000635955.1	pc104A	Complete	5.02	60.8	1	4431	2014-04-22T00:00:00Z
A. hydrophila	CP006883.1	SAMN03283200	PRJNA227242	GCA_000819505.1	J-1	Complete	5.00	60.9	1	4391	2015-01-16T00:00:00Z
A. hydrophila	CP016380.1	SAMN03294822	PRJNA273636	GCA_001687125.1	AHNIH1	Complete	5.05	61.1	2	4488	2016-07-18T00:00:00Z
A. hydrophila	CP010947.1	SAMN03366652	PRJNA270887	GCA_000940915.1	AL06-06	Complete	4.90	61.4	4	4382	2015-03-04T00:00:00Z
A. hydrophila	CP011100.1	SAMN03421355	PRJNA278509	GCA_000963645.1	AH10	Complete	4.91	61.1	1	4319	2015-03-26T00:00:00Z
A. hydrophila	CP006870.1	SAMN03744001	PRJNA226230	GCA_001019645.1	NJ-35	Complete	5.28	60.5	1	4689	2015-06-03T00:00:00Z
A. hydrophila	CP013178.1	SAMN04266592	PRJNA302121	GCA_001455365.1	JBN2301	Complete	5.15	60.8	4	4565	2015-12-02T00:00:00Z
A. hydrophila	CP013965.1	SAMN04404355	PRJNA308632	GCA_001518775.1	D4	Complete	5.28	60.5	5	4690	2016-01-20T00:00:00Z
A. hydrophila	CP016392.1	SAMN05188441	PRJNA323754	GCA_001683535.1	GYK1	Complete	4.95	60.8	1	4327	2016-07-11T00:00:00Z
A. hydrophila	CP016990.1	SAMN05589911	PRJNA339336	GCA_003491245.1	ZYAH75	Complete	4.96	61.3	1	4361	2018-09-07T00:00:00Z
A. hydrophila	CP016989.1	SAMN05590109	PRJNA339368	GCA_003491225.1	ZYAH72	Complete	5.16	60.7	1	4536	2018-09-07T00:00:00Z
A. hydrophila	CP018201.1	SAMN06016471	PRJNA353572	GCA_001895965.1	MX16A	Complete	4.78	61.6	1	4201	2016-12-09T00:00:00Z
A. hydrophila	CP028568.2	SAMN07821593	PRJNA415336	GCA_002850695.3	WCHAH045096	Complete	5.37	60.8	7	4730	2018-04-05T00:00:00Z
A. hydrophila	CP028418.1	SAMN08427316	PRJNA431712	GCA_003999465.1	WCX23	Complete	4.30	60.7	2	4326	2019-01-08T00:00:00Z
A. hydrophila	CP027804.1	SAMN08715271	PRJNA438415	GCA_003014775.1	KN-Mc-1R2	Complete	4.91	61.0	1	4386	2018-03-24T00:00:00Z
A. hydrophila	CP038463.1	SAMN11293745	PRJNA530076	GCA_004684305.1	WCX23	Complete	5.31	60.4	2	4710	2019-04-07T00:00:00Z
A. hydrophila	CP038465.1	SAMN11293757	PRJNA530083	GCA_004684325.1	23-C-23	Complete	5.28	60.5	2	4687	2019-04-07T00:00:00Z
A. hydrophila	CP043324.1	SAMN12097413	PRJNA528964	GCA_014218195.1	2359	Complete	5.04	61.0	1	2496	2020-08-17T00:00:00Z
A. hydrophila	CP043323.1	SAMN12097415	PRJNA528964	GCA_014217525.1	3206	Complete	4.79	61.6	1	2738	2020-08-17T00:00:00Z
A. hydrophila	CP045220.1	SAMN13041436	PRJNA577922	GCA_022700815.1	Aer_Brac66	Complete	4.75	61.5	1	4192	2022-03-28T00:00:00Z
A. hydrophila	CP045501.1	SAMN13041437	PRJNA577921	GCA_022700835.1	Aer_Pi25.1HTAS	Complete	4.81	61.2	1	4261	2022-03-28T00:00:00Z
A. hydrophila	CP045502.1	SAMN13104751	PRJNA579172	GCA_022700855.1	Aer_Brac14A	Complete	4.76	61.5	1	4195	2022-03-28T00:00:00Z
A. hydrophila	CP050850.1	SAMN13353934	PRJNA591199	GCA_017310195.1	Brac6	Complete	4.77	61.5	1	4195	2021-03-09T00:00:00Z
A. hydrophila	CP065651.1	SAMN13450446	PRJNA231221	GCA_016026875.1	FDAARGOS_916	Complete	4.73	61.6	1	4192	2020-12-14T00:00:00Z
A. hydrophila	CP050851.1	SAMN13545457	PRJNA595062	GCA_017310215.1	OnP3.1	Complete	4.77	61.5	1	4194	2021-03-09T00:00:00Z
A. hydrophila	CP046954.1	SAMN13567714	PRJNA595800	GCA_009791455.1	HX-3	Complete	4.94	61.0	1	4313	2019-12-23T00:00:00Z
A. hydrophila	CP053885.1	SAMN13926349	PRJNA528964	GCA_018802385.1	3019	Complete	5.13	60.9	1	4527	2021-06-09T00:00:00Z
A. hydrophila	CP053884.1	SAMN13926350	PRJNA528964	GCA_018802345.1	3924	Complete	4.76	61.4	1	3638	2021-06-09T00:00:00Z
A. hydrophila	CP053883.1	SAMN13926351	PRJNA528964	GCA_018802305.1	4960	Complete	4.83	61.5	1	4079	2021-06-09T00:00:00Z
A. hydrophila	CP064382.1	SAMN14078805	PRJNA605254	GCA_022869925.1	PartN-Ahydrophila-RM837	Complete	4.73	61.6	1	4191	2022-04-11T00:00:00Z
A. hydrophila	CP050012.1	SAMN14351661	PRJNA611874	GCA_011602425.1	LHW39	Complete	5.10	60.8	1	4506	2020-03-21T00:00:00Z
A. hydrophila	CP050994.1	SAMN14533640	PRJNA622823	GCA_012273595.1	NEB724	Complete	4.57	61.7	1	3946	2020-04-12T00:00:00Z
A. hydrophila	CP053859.1	SAMN15009239	PRJNA634798	GCA_013205705.1	B11	Complete	4.78	61.8	1	4158	2020-06-02T00:00:00Z
A. hydrophila	CP084353.1	SAMN21168046	PRJNA759416	GCA_020405385.1	71317	Complete	4.70	61.7	1	4099	2021-10-10T00:00:00Z

A. hydrophila	CP084352.1	SAMN21168048	PRJNA759416	GCA_020405365.1	71339	Complete	4.79	61.4	1	4196	2021-10-10T00:00:00Z
A. hydrophila	CP083944.1	SAMN21540782	PRJNA765017	GCA_020162255.1	CSUSB2	Complete	4.92	61.0	3	4337	2021-09-28T00:00:00Z
A. hydrophila	CP092906.1	SAMN26244239	PRJNA810314	GCA_022557195.1	LP0103	Complete	5.02	60.9	1	4369	2022-03-13T00:00:00Z
A. hydrophila	CP093309.1	SAMN26377313	PRJNA812366	GCA_022631195.1	AC133	Complete	5.04	60.9	1	4400	2022-03-20T00:00:00Z
A. hydrophila	CP093308.1	SAMN26377314	PRJNA812366	GCA_022631175.1	AC185	Complete	4.96	61.3	1	4375	2022-03-20T00:00:00Z
A. hydrophila	CP094267.1	SAMN26806291	PRJNA817329	GCA_022759545.1	A008N2	Complete	4.78	61.6	1	4205	2022-03-30T00:00:00Z
A. hydrophila	CP095280.1	SAMN27350375	PRJNA823662	GCA_022982835.1	Ah2111	Complete	5.32	60.7	8	4724	2022-04-14T00:00:00Z
A. jandaei	AP024466.1	SAMD00276024	PRJDB11115	GCA_016865345.1	JUNP479	Complete	4.54	59.0	2	4034	2021-02-02T00:00:00Z
A. jandaei	CP043322.1	SAMN12097416	PRJNA528964	GCA_014217505.1	3299	Complete	4.58	58.9	1	3389	2020-08-17T00:00:00Z
A. jandaei	CP043321.1	SAMN12097417	PRJNA528964	GCA_014217485.1	3348	Complete	4.57	58.9	1	2499	2020-08-17T00:00:00Z
A. jandaei	CP053882.1	SAMN13926352	PRJNA528964	GCA_018802365.1	3036	Complete	4.59	59.0	1	3936	2021-06-09T00:00:00Z
A. jandaei	CP053881.1	SAMN13926353	PRJNA528964	GCA_018802325.1	4608	Complete	4.51	59.0	1	4004	2021-06-09T00:00:00Z
A. jandaei	CP053879.1	SAMN13926354	PRJNA528964	GCA_018802265.1	4956	Complete	4.65	58.5	2	3742	2021-06-09T00:00:00Z
A. jandaei	CP066092.1	SAMN16357155	PRJNA231221	GCA_016127195.1	FDAARGOS_986	Complete	4.55	59.0	1	4071	2020-12-20T00:00:00Z
A. media	AP022188.1	SAMD00194563	PRJDB6962	GCA_014161425.1	WP7-W18-ESBL-02	Complete	4.76	61.0	1	4165	2020-07-21T23:42:00Z
A. media	CP007567.1	SAMN02472129	PRJNA170164	GCA_000287215.3	WS	Complete	4.79	60.7	2	4089	2014-04-22T00:00:00Z
A. media	CP038441.1	SAMN11269565	PRJNA529511	GCA_013085485.1	T0.1-19	Complete	4.92	61.0	2	4269	2020-05-14T00:00:00Z
A. media	CP038443.1	SAMN11269566	PRJNA529511	GCA_013085725.1	R1-18	Complete	4.74	60.9	1	4006	2020-05-14T00:00:00Z
A. media	CP038444.1	SAMN11269567	PRJNA529511	GCA_013085745.1	T5-8	Complete	4.79	60.8	1	4133	2020-05-14T00:00:00Z
A. media	CP038445.1	SAMN11269568	PRJNA529511	GCA_013085765.1	R25-3	Complete	4.88	60.6	3	4233	2020-05-14T00:00:00Z
A. media	CP038448.1	SAMN11269569	PRJNA529511	GCA_013085785.1	R50-22	Complete	5.15	60.2	3	4430	2020-05-14T00:00:00Z
A. media	CP043579.1	SAMN12671375	PRJNA563308	GCA_014109865.1	R1-26	Complete	4.67	60.8	1	4069	2020-08-07T00:00:00Z
A. media	CP047962.1	SAMN13876930	PRJNA601918	GCA_009931135.1	MC64	Complete	5.02	60.4	3	4288	2020-01-27T00:00:00Z
A. media	CP061477.1	SAMN15963531	PRJNA660910	GCA_019455365.1	T5-1	Complete	5.04	60.9	2	3856	2021-08-05T00:00:00Z
A. media	CP067417.1	SAMN17256260	PRJNA690656	GCA_016653695.1	E31	Complete	5.31	60.2	3	4526	2021-01-17T00:00:00Z
A. media	CP075564.1	SAMN19237318	PRJNA730636	GCA_020423125.1	TR3_1	Complete	4.53	61.2	2	3954	2021-10-12T00:00:00Z
A. rivipollensis	CP027856.1	SAMN08721782	PRJNA438570	GCA_003015165.1	KN-Mc-11N1	Complete	4.51	61.9	1	3916	2018-03-26T00:00:00Z
A. rivuli	CP079742.1	SAMN20256631	PRJNA747139	GCA_020149575.1	20-VB00005	Complete	4.36	59.9	1	3829	2021-09-27T00:00:00Z
A. salmonicida subsp. *salmoni*	CP027000.1	SAMN02469939	PRJNA264317	GCA_003692675.1	01-B526	Contig	4.93	58.3	604	4330	2011-11-10T00:00:00Z
A. salmonicida subsp. *salmoni*	CP000644.1	SAMN02603664	PRJNA16723	GCA_000196395.1	A449	Complete	5.04	58.2	6	4435	2007-04-06T00:00:00Z
A. salmonicida subsp. *pectinol*	CP022426.1	SAMN04208118	PRJNA299262	GCA_002735225.1	34mel	Complete	5.01	58.3	1	4336	2017-10-27T00:00:00Z
A. salmonicida subsp. *salmoni*	CP048223.1	SAMN04449844	PRJNA310296	GCA_001643275.2	J223	Complete	4.90	58.4	5	4306	2020-01-29T00:00:00Z
A. salmonicida subsp. *masouc*	CP017143.1	SAMN05735119	PRJNA342065	GCA_002313065.1	RFAS1	Complete	4.92	58.3	3	4220	2017-09-19T00:00:00Z
A. salmonicida	CP021654.1	SAMN06812489	PRJNA383853	GCA_002180335.1	O23A	Complete	4.94	58.1	5	4379	2017-06-08T00:00:00Z
A. salmonicida	CP022175.1	SAMN07276469	PRJNA391818	GCA_002214245.1	S121	Complete	5.07	58.2	6	4389	2017-07-03T00:00:00Z
A. salmonicida	CP022186.1	SAMN07276873	PRJNA391844	GCA_002214265.1	S68	Complete	4.88	58.4	5	4216	2017-07-03T00:00:00Z
A. salmonicida	CP022181.1	SAMN07276874	PRJNA391845	GCA_002214305.1	S44	Complete	5.08	58.2	6	4396	2017-07-03T00:00:00Z
A. salmonicida	CP022550.1	SAMN07410266	PRJNA264317	GCA_002764135.1	A527	Complete	4.81	58.7	1	4176	2017-11-13T00:00:00Z
A. salmonicida subsp. *salmoni*	CP038102.1	SAMN11172869	PRJNA264317	GCA_008370735.1	SHY16-3432	Complete	5.01	58.3	4	4432	2019-09-12T00:00:00Z
A. salmonicida subsp. *salmoni*	CP047374.1	SAMN13518349	PRJNA594426	GCA_009858115.1	J409	Complete	4.73	58.6	2	4031	2020-01-13T00:00:00Z
A. salmonicida subsp. *salmoni*	CP047376.1	SAMN13518350	PRJNA594426	GCA_009858135.1	J410	Complete	4.69	58.6	2	4066	2020-01-13T00:00:00Z
A. salmonicida subsp. *salmoni*	CP052034.1	SAMN13518351	PRJNA594426	GCA_012933685.1	J411	Complete	4.72	58.6	2	3456	2020-04-30T00:00:00Z
A. salmonicida subsp. *masouc*	CP060030.1	SAMN14260301	PRJNA609824	GCA_014872735.1	BR19001YR	Complete	4.98	58.2	4	4335	2020-10-08T00:00:00Z
A. salmonicida subsp. *masouc*	CP049830.1	SAMN14325646	PRJNA610908	GCA_019443805.1	RZ6S-1	Complete	5.19	58.1	4	4363	2021-08-03T00:00:00Z
A. salmonicida subsp. *masouc*	CP050187.1	SAMN14351711	PRJNA611880	GCA_019443825.1	RZ6S-1	Complete	5.06	58.2	4	4189	2021-08-03T00:00:00Z
A. salmonicida	CP051883.1	SAMN14515089	PRJNA622411	GCA_012931585.1	SRW-OG1	Complete	4.62	59.0	1	4049	2020-04-29T00:00:00Z
A. salmonicida subsp. *salmoni*	CP085533.1	SAMN22480857	PRJNA773379	GCA_020683025.1	890054	Complete	5.04	58.2	9	4491	2021-10-31T00:00:00Z
A. sanarellii	CP079751.1	SAMN20179300	PRJNA746053	GCA_019334425.1	NS1	Complete	5.21	61.5	1	4201	2021-07-25T00:00:00Z
A. schubertii	CP013067.1	SAMN04110141	PRJNA297116	GCA_001447335.1	WL1483	Complete	4.40	61.5	1	4004	2015-11-23T00:00:00Z
A. schubertii	CP039611.1	SAMN11129962	PRJNA527158	GCA_004919485.1	LF1708	Complete	4.35	61.4	1	3790	2019-04-28T00:00:00Z
A. simiae	CP040449.1	SAMN11664857	PRJNA543361	GCA_014892695.1	A6	Complete	3.97	60.6	1	3565	2019-10-09T00:00:00Z
A. taiwanensis	CP081026.1	SAMN17793875	PRJNA668870	GCA_019646115.1	Colony382	Chromosc	4.53	58.9	1	NA	2021-08-17T00:00:00Z
A. veronii	AP021940.1	SAMD00194363	PRJDB6962	GCA_014168715.1	WP2-S18-CRE-03	Complete	4.94	58.6	4	4401	2020-07-21T23:12:00Z
A. veronii	AP022038.1	SAMD00194437	PRJDB6962	GCA_014168995.1	WP3-W19-ESBL-03	Complete	4.98	58.7	6	4344	2020-07-21T23:24:00Z
A. veronii	AP022264.1	SAMD00194633	PRJDB6962	GCA_014169795.1	WP8-S18-ESBL-11	Complete	4.91	58.7	4	4318	2020-07-21T23:55:00Z
A. veronii	AP022281.1	SAMD00194639	PRJDB6962	GCA_014169835.1	WP8-W19-CRE-03	Complete	4.79	58.5	6	4221	2020-07-21T23:57:00Z
A. veronii	AP022290.1	SAMD00194675	PRJDB6962	GCA_014169875.1	WP9-W18-ESBL-04	Complete	4.93	58.7	5	4322	2020-07-21T23:58:00Z
A. veronii	CP059396.1	SAMN02471703	PRJNA205862	GCA_000464515.2	Hm21	Complete	4.77	58.7	2	4223	2020-07-31T00:00:00Z
A. veronii	CP002607.1	SAMN02603940	PRJNA63671	GCA_000204115.1	B565	Complete	4.55	58.7	1	4027	2011-04-13T00:00:00Z
A. veronii	CP014774.1	SAMN03455926	PRJNA279607	GCA_001634325.1	AVNIH1	Complete	4.96	58.5	2	4366	2016-04-29T00:00:00Z
A. veronii	CP012504.1	SAMN04012505	PRJNA293940	GCA_001593245.1	TH0426	Complete	4.92	58.3	1	4372	2016-03-16T00:00:00Z
A. veronii	CP015448.1	SAMN04901561	PRJNA319612	GCA_001634345.1	CB51	Complete	4.58	58.6	1	3623	2016-04-29T00:00:00Z
A. veronii	CP024930.1	SAMN08042683	PRJNA419084	GCA_002803925.1	X11	Complete	4.28	58.8	1	3726	2017-12-04T00:00:00Z
A. veronii	CP024933.1	SAMN08042684	PRJNA419086	GCA_002803945.1	X12	Complete	4.77	58.3	1	4212	2017-12-04T00:00:00Z
A. veronii	CP032839.1	SAMN08281233	PRJNA428153	GCA_002866885.2	FC951	Complete	4.86	58.7	2	4256	2018-10-10T00:00:00Z
A. veronii	CP028133.1	SAMN08731619	PRJNA438884	GCA_003491365.1	17ISAe	Complete	4.66	58.5	2	4063	2018-09-07T00:00:00Z
A. veronii	CP033604.1	SAMN10389897	PRJNA504296	GCA_003722175.1	MS-18-37	Complete	4.68	58.6	1	4088	2018-11-13T00:00:00Z
A. veronii	CP034967.1	SAMN10696333	PRJNA513116	GCA_011045495.1	ZfB1	Complete	4.71	58.5	1	4169	2020-03-02T00:00:00Z
A. veronii	CP044060.1	SAMN11056347	PRJNA231221	GCA_008693705.1	FDAARGOS_632	Complete	4.56	58.9	2	4016	2019-09-25T00:00:00Z
A. veronii	CP040717.1	SAMN11868773	PRJNA545011	GCA_009755745.1	HX3	Complete	4.76	58.5	2	4176	2019-12-16T00:00:00Z
A. veronii	CP046407.1	SAMN13220457	PRJNA587884	GCA_009833025.1	A8-AHP	Complete	4.77	58.4	4	4205	2020-01-07T00:00:00Z
A. veronii	CP047155.1	SAMN13640848	PRJNA596872	GCA_009834065.1	AVNIH1	Complete	4.81	58.5	1	4263	2020-01-08T00:00:00Z
A. veronii	CP054855.1	SAMN15184730	PRJNA638265	GCA_017310275.1	1708-29120	Complete	4.50	58.9	1	3926	2021-03-09T00:00:00Z
A. veronii	CP058912.1	SAMN15395818	PRJNA642679	GCA_013415825.1	JC529	Complete	4.83	58.3	1	4282	2020-07-15T00:00:00Z
A. veronii	CP066125.1	SAMN17038035	PRJNA683808	GCA_020544445.1	32286	Complete	5.02	58.4	1	4444	2021-10-25T00:00:00Z
A. veronii	CP087266.1	SAMN20179696	PRJNA780685	GCA_022921155.1	HD6448	Complete	5.20	58.6	6	4612	2022-04-12T00:00:00Z
A. veronii	CP080630.1	SAMN20667377	PRJNA749892	GCA_021586915.1	A29V	Complete	4.54	58.7	3	3973	2022-01-25T00:00:00Z
A. veronii	CP085465.1	SAMN21168054	PRJNA759416	GCA_020640955.1	71506	Complete	4.69	58.4	3	4071	2021-10-27T00:00:00Z
A. veronii	CP083461.2	SAMN21418941	PRJNA762937	GCA_020172765.2	SW3814	Complete	4.84	58.2	3	4290	2021-09-29T00:00:00Z
A. veronii	CP095841.1	SAMN27615187	PRJNA827608	GCA_023100745.1	AV040	Complete	4.50	58.8	1	3930	2022-04-25T00:00:00Z
A. caviae	CP080052.1	SAMN17793873	PRJNA668870	GCA_019395125.1	Colony22	Chromosc	4.53	61.1	1	NA	2021-07-29T00:00:00Z
A. caviae	CP080051.1	SAMN17793874	PRJNA668870	GCA_019395105.1	Colony26	Chromosc	4.53	61.1	1	NA	2021-07-29T00:00:00Z
A. caviae	CP080050.1	SAMN17793876	PRJNA668870	GCA_019395085.1	Colony109	Chromosc	4.53	60.9	1	NA	2021-07-29T00:00:00Z
A. caviae	CP080049.1	SAMN17793877	PRJNA668870	GCA_019395065.1	Colony57	Chromosc	4.68	60.8	1	3567	2021-07-29T00:00:00Z
A. caviae	CP080048.1	SAMN17793878	PRJNA668870	GCA_019395045.1	Colony71	Chromosc	4.68	60.6	1	3545	2021-07-29T00:00:00Z
A. caviae	CP081290.1	SAMN17793887	PRJNA668870	GCA_019711295.1	Colony274	Chromosc	4.68	60.7	1	3625	2021-08-22T00:00:00Z
A. caviae	CP081293.1	SAMN18017934	PRJNA668870	GCA_019711275.1	Colony23	Chromosc	4.68	60.8	1	3599	2021-08-22T00:00:00Z
A. dhakensis	CP046626.1	SAMN13512574	PRJNA594314	GCA_017310095.1	Aer_On24M	Chromosc	4.93	61.4	1	4362	2021-03-09T00:00:00Z
A. dhakensis	CP070219.1	SAMN17793893	PRJNA668870	GCA_016902895.1	Colony496	Chromosc	4.58	56.3	1	NA	2021-02-17T00:00:00Z
A. dhakensis	CP070218.1	SAMN17793897	PRJNA668870	GCA_016902835.1	Colony59	Chromosc	4.58	56.2	1	NA	2021-02-17T00:00:00Z
A. dhakensis	CP070217.1	SAMN17793902	PRJNA668870	GCA_016902755.1	Colony437	Chromosc	4.58	56.3	1	NA	2021-02-17T00:00:00Z

A. dhakensis	CP075589.1	SAMN18017937	PRJNA668870	GCA_018596395.1	Colony37	Chromosc	5.10	60.2	1	3468	2021-05-30T00:00:00Z
A. encheleia	CP093843.1	SAMN26533773	PRJNA631893	GCA_022691185.1	H4-C21	Chromosc	4.62	61.7	1	3419	2022-03-24T00:00:00Z
A. enteropelogenes	CP081025.1	SAMN17793872	PRJNA668870	GCA_019646135.1	Colony21	Chromosc	4.53	54.9	1	NA	2021-08-17T00:00:00Z
A. enteropelogenes	CP084358.1	SAMN21402704	PRJNA231221	GCA_020341435.1	FDAARGOS_1537	Chromosc	4.35	59.9	1	3809	2021-10-07T00:00:00Z
A. hydrophila	CP046604.1	SAMN13507464	PRJNA594183	GCA_017310075.1	Aer_LaG34	Chromosc	4.72	61.5	1	4195	2021-03-09T00:00:00Z
A. hydrophila	CP046869.1	SAMN13514045	PRJNA594373	GCA_017310155.1	Aer_OnP2.2	Chromosc	4.75	61.5	1	4194	2021-03-09T00:00:00Z
A. hydrophila	CP046870.1	SAMN13514046	PRJNA594374	GCA_017310115.1	Aer_OnP4.2	Chromosc	4.75	61.5	1	4208	2021-03-09T00:00:00Z
A. hydrophila	CP046871.1	SAMN13540891	PRJNA594926	GCA_017310135.1	Aer_LaG33	Chromosc	4.75	61.5	1	4194	2021-03-09T00:00:00Z
A. hydrophila	CP092708.1	SAMN26095833	PRJNA806525	GCA_022488365.1	S-P-C-021.01	Chromosc	4.77	61.5	1	4193	2022-03-06T00:00:00Z
A. hydrophila	CP092709.1	SAMN26095834	PRJNA806525	GCA_022488385.1	S-P-C-022.01	Chromosc	4.77	61.5	1	4193	2022-03-06T00:00:00Z
A. jandaei	CP046270.1	SAMN13335576	PRJNA590577	GCA_017750905.1	GTBM29	Chromosc	4.71	58.7	1	4278	2021-04-05T00:00:00Z
A. jandaei	CP050701.1	SAMN13547657	PRJNA595181	GCA_017310175.1	Aer_Pi19.1HPAS	Chromosc	4.55	58.9	2	4116	2021-03-09T00:00:00Z
A. jandaei	CP070221.1	SAMN17793884	PRJNA668870	GCA_016902955.1	Colony119	Chromosc	5.10	54.8	1	NA	2021-02-17T00:00:00Z
A. jandaei	CP070220.1	SAMN17793891	PRJNA668870	GCA_016902935.1	Colony25	Chromosc	4.57	56.4	1	NA	2021-02-17T00:00:00Z
A. jandaei	CP084376.1	SAMN21402690	PRJNA231221	GCA_020341535.1	FDAARGOS_1523	Chromosc	4.55	59.0	1	4066	2021-10-07T00:00:00Z
A. media	CP070623.1	SAMN17978689	PRJNA668870	GCA_017068335.1	Colony414	Chromosc	4.75	60.0	1	3535	2021-02-28T00:00:00Z
A. salmonicida	CP080045.1	SAMN17793885	PRJNA668870	GCA_019394985.1	Colony462	Chromosc	4.81	58.6	1	3794	2021-07-29T00:00:00Z
A. salmonicida	CP080044.1	SAMN17793886	PRJNA668870	GCA_019394965.1	Colony197	Chromosc	4.81	58.6	1	3803	2021-07-29T00:00:00Z
A. salmonicida	CP080043.1	SAMN17793888	PRJNA668870	GCA_019394925.1	Colony314	Chromosc	4.81	58.6	1	3720	2021-07-29T00:00:00Z
A. salmonicida	CP080042.1	SAMN17793889	PRJNA668870	GCA_019394845.1	Colony381	Chromosc	4.81	58.5	1	3697	2021-07-29T00:00:00Z
A. salmonicida	CP080041.1	SAMN17793890	PRJNA668870	GCA_019394755.1	Colony418	Chromosc	4.81	58.3	1	3566	2021-07-29T00:00:00Z
A. veronii	CP070213.1	SAMN17793892	PRJNA668870	GCA_016902915.1	Colony125	Chromosc	4.58	58.0	1	3554	2021-02-17T00:00:00Z
A. veronii	CP070212.1	SAMN17793894	PRJNA668870	GCA_016902875.1	Colony512	Chromosc	4.60	58.5	1	3642	2021-02-17T00:00:00Z
A. veronii	CP080040.1	SAMN17793895	PRJNA668870	GCA_019394685.1	Colony54	Chromosc	4.57	57.3	1	NA	2021-07-29T00:00:00Z
A. veronii	CP070211.1	SAMN17793896	PRJNA668870	GCA_016902855.1	Colony58	Chromosc	4.58	58.0	1	3551	2021-02-17T00:00:00Z
A. veronii	CP070210.1	SAMN17793898	PRJNA668870	GCA_016902815.1	Colony108	Chromosc	4.57	58.0	1	3529	2021-02-17T00:00:00Z
A. veronii	CP070209.1	SAMN17793899	PRJNA668870	GCA_016902795.1	Colony112	Chromosc	4.58	57.5	1	NA	2021-02-17T00:00:00Z
A. veronii	CP070208.1	SAMN17793900	PRJNA668870	GCA_016902775.1	Colony121	Chromosc	4.57	58.0	1	3538	2021-02-17T00:00:00Z
A. veronii	CP070207.1	SAMN17793901	PRJNA668870	GCA_016902735.1	Colony111	Chromosc	4.58	57.4	1	NA	2021-02-17T00:00:00Z
A. veronii	CP070206.1	SAMN17793903	PRJNA668870	GCA_016902715.1	Colony27	Chromosc	4.57	57.5	1	NA	2021-02-17T00:00:00Z
A. veronii	CP070205.1	SAMN17793904	PRJNA668870	GCA_016902695.1	Colony604	Chromosc	4.57	57.5	1	NA	2021-02-17T00:00:00Z
A. allosaccharophila	CAAKNO01	SAMEA5282779	PRJEB31025	GCA_901212385.1	KLG2	Contig	4.51	58.9	85	4026	2019-05-08T00:00:00Z
A. allosaccharophila	JAGDER01	SAMN17101840	PRJNA685948	GCA_018359965.1	CECT4911	Contig	4.87	58.6	138	4288	2021-05-14T00:00:00Z
A. allosaccharophila	JAKCNX01	SAMN24245014	PRJNA762299	GCA_021441305.1	INSAq241	Contig	4.51	58.8	104	4026	2022-01-11T00:00:00Z
A. allosaccharophila	LKKI01	SAMN04099669	PRJNA296464	GCA_001446895.1	TTU2014-159ASC	Contig	4.58	59.0	48	4039	2015-11-19T00:00:00Z
A. allosaccharophila	MRZP01	SAMN05860754	PRJNA345312	GCA_001908545.1	CCM 4363	Contig	4.72	58.3	199	3922	2016-12-19T00:00:00Z
A. aquatica	JRGL01	SAMN03023875	PRJNA260478	GCA_000764655.1	AE235	Contig	4.58	61.2	171	4100	2014-10-08T00:00:00Z
A. australiensis	CDDH01	SAMEA2752426	PRJEB7021	GCA_000819725.1	CECT 8023	Contig	4.11	58.1	113	3640	2014-11-24T00:00:00Z
A. australiensis	RSEA01	SAMN10473215	PRJNA506732	GCA_021538905.1	Aer294	Contig	4.64	57.6	519	4190	2022-01-20T00:00:00Z
A. bestiarum	JPWL01	SAMN02954503	PRJNA257751	GCA_000746985.1	CBA100	Contig	4.79	60.5	118	4241	2014-09-02T00:00:00Z
A. bestiarum	PPUX01	SAMN08391293	PRJNA431444	GCA_002906925.1	GA97-22	Contig	4.71	60.5	26	4178	2018-01-30T00:00:00Z
A. caviae	BPNA01	SAMD00294551	PRJDB11802	GCA_019972535.1	KAM333	Contig	4.57	61.2	472	4142	2021-07-14T21:28:00Z
A. caviae	BPNB01	SAMD00294552	PRJDB11802	GCA_019972515.1	KAM334	Contig	4.55	61.3	214	4048	2021-07-14T21:18:00Z
A. caviae	BPNC01	SAMD00294553	PRJDB11802	GCA_019972495.1	KAM335	Contig	4.58	61.3	318	4132	2021-07-14T21:27:00Z
A. caviae	BPND01	SAMD00294554	PRJDB11802	GCA_019972475.1	KAM336	Contig	4.89	60.7	426	4453	2021-07-14T22:08:00Z
A. caviae	BPNE01	SAMD00294555	PRJDB11802	GCA_019972455.1	KAM337	Contig	4.76	61.0	313	4313	2021-07-14T21:49:00Z
A. caviae	BPNF01	SAMD00294557	PRJDB11802	GCA_019971135.1	KAM340	Contig	4.88	60.7	371	4453	2021-07-14T22:07:00Z
A. caviae	BPNG01	SAMD00294558	PRJDB11802	GCA_019971155.1	KAM341	Contig	4.89	60.4	526	4404	2021-07-14T22:11:00Z
A. caviae	BPNH01	SAMD00294559	PRJDB11802	GCA_019971175.1	KAM342	Contig	4.92	60.4	536	4403	2021-07-14T22:14:00Z
A. caviae	BPNI01	SAMD00294560	PRJDB11802	GCA_019971195.1	KAM343	Contig	4.97	60.5	562	4493	2021-07-14T22:17:00Z
A. caviae	BPNJ01	SAMD00294562	PRJDB11802	GCA_019971215.1	KAM346	Contig	4.89	61.1	390	4451	2021-07-14T22:09:00Z
A. caviae	BPNK01	SAMD00294563	PRJDB11802	GCA_019971305.1	KAM347	Contig	4.81	60.9	527	4415	2021-07-14T22:06:00Z
A. caviae	BPNL01	SAMD00294564	PRJDB11802	GCA_019971385.1	KAM348	Contig	4.81	61.3	294	4395	2021-07-14T21:45:00Z
A. caviae	BPNM01	SAMD00294565	PRJDB11802	GCA_019971485.1	KAM350	Contig	4.80	60.9	437	4388	2021-07-14T22:03:00Z
A. caviae	BPNN01	SAMD00294566	PRJDB11802	GCA_019971515.1	KAM351	Contig	4.97	60.7	320	4551	2021-07-14T22:15:00Z
A. caviae	BPNO01	SAMD00294567	PRJDB11802	GCA_019971575.1	KAM352	Contig	4.76	60.9	394	4334	2021-07-14T21:57:00Z
A. caviae	BPNP01	SAMD00294568	PRJDB11802	GCA_019971595.1	KAM353	Contig	4.83	61.0	451	4415	2021-07-14T22:05:00Z
A. caviae	BPNQ01	SAMD00294569	PRJDB11802	GCA_019971615.1	KAM354	Contig	5.13	60.4	622	4666	2021-07-14T22:19:00Z
A. caviae	BPNR01	SAMD00294570	PRJDB11802	GCA_019971635.1	KAM355	Contig	4.83	61.2	282	4411	2021-07-14T21:58:00Z
A. caviae	BPNS01	SAMD00294571	PRJDB11802	GCA_019971655.1	KAM356	Contig	4.54	61.4	323	4079	2021-07-14T21:23:00Z
A. caviae	BPNT01	SAMD00294572	PRJDB11802	GCA_019971675.1	KAM357	Contig	4.53	61.5	276	4059	2021-07-14T21:26:00Z
A. caviae	BPNU01	SAMD00294573	PRJDB11802	GCA_019971695.1	KAM358	Contig	4.91	60.6	643	4429	2021-07-14T22:16:00Z
A. caviae	BPNV01	SAMD00294574	PRJDB11802	GCA_019971715.1	KAM359	Contig	4.79	61.3	181	4374	2021-07-14T21:36:00Z
A. caviae	BPNW01	SAMD00294575	PRJDB11802	GCA_019971735.1	KAM360	Contig	4.75	60.9	531	4296	2021-07-14T21:43:00Z
A. caviae	BPNX01	SAMD00294576	PRJDB11802	GCA_019971755.1	KAM361	Contig	4.53	61.5	299	4065	2021-07-14T21:24:00Z
A. caviae	BPNY01	SAMD00294577	PRJDB11802	GCA_019971775.1	KAM362	Contig	4.86	61.2	251	4446	2021-07-14T22:00:00Z
A. caviae	BPNZ01	SAMD00294578	PRJDB11802	GCA_019971795.1	KAM363	Contig	4.52	61.5	284	4057	2021-07-14T21:21:00Z
A. caviae	BPOA01	SAMD00294579	PRJDB11802	GCA_019971815.1	KAM364	Contig	4.58	61.3	343	4119	2021-07-14T21:30:00Z
A. caviae	BPOB01	SAMD00294580	PRJDB11802	GCA_019971835.1	KAM365	Contig	4.86	61.2	250	4449	2021-07-14T22:01:00Z
A. caviae	BPOC01	SAMD00294581	PRJDB11802	GCA_019971855.1	KAM366	Contig	4.52	61.5	287	4049	2021-07-14T21:20:00Z
A. caviae	BPOD01	SAMD00294582	PRJDB11802	GCA_019971875.1	KAM367	Contig	4.86	61.2	251	4454	2021-07-14T21:59:00Z
A. caviae	BPOE01	SAMD00294583	PRJDB11802	GCA_019971895.1	KAM368	Contig	4.52	61.5	290	4053	2021-07-14T21:25:00Z
A. caviae	BPOF01	SAMD00294584	PRJDB11802	GCA_019971915.1	KAM369	Contig	4.84	61.3	172	4434	2021-07-14T21:41:00Z
A. caviae	BPOG01	SAMD00294585	PRJDB11802	GCA_019971935.1	KAM370	Contig	4.79	61.3	150	4382	2021-07-14T21:35:00Z
A. caviae	BPOH01	SAMD00294586	PRJDB11802	GCA_019971955.1	KAM372	Contig	4.78	61.3	153	4375	2021-07-14T21:32:00Z
A. caviae	BPOI01	SAMD00294587	PRJDB11802	GCA_019971975.1	KAM373	Contig	4.78	61.3	162	4372	2021-07-14T21:36:00Z
A. caviae	BPOJ01	SAMD00294588	PRJDB11802	GCA_019971995.1	KAM374	Contig	4.79	61.2	217	4400	2021-07-14T21:40:00Z
A. caviae	BPOK01	SAMD00294589	PRJDB11802	GCA_019972015.1	KAM375	Contig	4.83	61.3	166	4429	2021-07-14T21:41:00Z
A. caviae	BPOL01	SAMD00294591	PRJDB11802	GCA_019972035.1	KAM378	Contig	4.82	61.2	235	4396	2021-07-14T21:56:00Z
A. caviae	BPOM01	SAMD00294592	PRJDB11802	GCA_019972575.1	KAM379	Contig	4.89	61.3	163	4500	2021-07-14T21:53:00Z
A. caviae	BPOO01	SAMD00294594	PRJDB11802	GCA_019972615.1	KAM381	Contig	4.78	61.3	157	4382	2021-07-14T21:34:00Z
A. caviae	BPOP01	SAMD00294595	PRJDB11802	GCA_019972635.1	KAM382	Contig	4.82	61.2	234	4389	2021-07-14T21:44:00Z
A. caviae	BPOQ01	SAMD00294596	PRJDB11802	GCA_019972655.1	KAM383	Contig	4.72	61.0	373	4287	2021-07-14T21:48:00Z
A. caviae	BPOR01	SAMD00294597	PRJDB11802	GCA_019972675.1	KAM384	Contig	4.65	61.3	291	4221	2021-07-14T21:31:00Z
A. caviae	BPOV01	SAMD00294590	PRJDB11802	GCA_019972715.1	KAM377	Contig	4.81	61.4	148	4400	2021-07-14T21:32:00Z
A. caviae	BPOW01	SAMD00334391	PRJDB11802	GCA_019972735.1	KAM329	Contig	4.91	60.8	371	4472	2021-07-14T22:12:00Z
A. caviae	BPOX01	SAMD00334392	PRJDB11802	GCA_019972755.1	KAM376	Contig	4.84	61.4	176	4437	2021-07-14T21:38:00Z
A. caviae	BQUX01	SAMD00432548	PRJDB11802	GCA_022835715.1	KAM338	Contig	5.48	60.7	296	5012	2022-03-05T00:00:00Z
A. caviae	BQUY01	SAMD00432549	PRJDB11802	GCA_022835735.1	KAM344	Contig	4.63	61.1	515	4222	2022-03-05T00:00:00Z
A. caviae	BQUZ01	SAMD00432550	PRJDB11802	GCA_022835755.1	KAM371	Contig	4.82	61.2	218	4420	2022-03-05T00:00:00Z
A. caviae	BQVA01	SAMD00432551	PRJDB11802	GCA_022835775.1	KAM447	Contig	4.67	61.2	355	4226	2022-03-05T00:00:00Z

A. caviae	BQVB01	SAMD00432552	PRJDB11802	GCA_022835795.1	KAM448	Contig	4.85	61.2	245	4443	2022-03-05T00:00:00Z
A. caviae	BQVC01	SAMD00432553	PRJDB11802	GCA_022835815.1	KAM449	Contig	4.82	61.2	217	4412	2022-03-05T00:00:00Z
A. caviae	BQVD01	SAMD00432554	PRJDB11802	GCA_022835835.1	KAM450	Contig	4.81	61.3	240	4381	2022-03-05T00:00:00Z
A. caviae	BQVE01	SAMD00432555	PRJDB11802	GCA_022835855.1	KAM451	Contig	4.81	61.2	214	4413	2022-03-05T00:00:00Z
A. caviae	BQVG01	SAMD00432557	PRJDB11802	GCA_022835895.1	KAM462	Contig	4.72	60.9	805	4277	2022-03-05T00:00:00Z
A. caviae	BQVH01	SAMD00432558	PRJDB11802	GCA_022835915.1	KAM463	Contig	4.46	61.5	412	4012	2022-03-05T00:00:00Z
A. caviae	BQVI01	SAMD00432559	PRJDB11802	GCA_022835935.1	KAM465	Contig	4.69	60.9	474	4203	2022-03-05T00:00:00Z
A. caviae	BQVJ01	SAMD00432560	PRJDB11802	GCA_022835955.1	KAM466	Contig	4.68	60.9	478	4200	2022-03-05T00:00:00Z
A. caviae	BQVK01	SAMD00432561	PRJDB11802	GCA_022835975.1	KAM467	Contig	4.75	60.9	543	4233	2022-03-05T00:00:00Z
A. caviae	BQVL01	SAMD00432562	PRJDB11802	GCA_022835995.1	KAM468	Contig	4.69	60.9	470	4205	2022-03-05T00:00:00Z
A. caviae	BQVM01	SAMD00432563	PRJDB11802	GCA_022836015.1	KAM469	Contig	4.77	60.9	476	4293	2022-03-05T00:00:00Z
A. caviae	BQVN01	SAMD00432564	PRJDB11802	GCA_022836035.1	KAM470	Contig	4.72	60.9	499	4245	2022-03-05T00:00:00Z
A. caviae	BQVO01	SAMD00432565	PRJDB11802	GCA_022836055.1	KAM471	Contig	4.48	61.5	348	4047	2022-03-05T00:00:00Z
A. caviae	BQVP01	SAMD00432566	PRJDB11802	GCA_022836075.1	KAM472	Contig	4.73	60.9	766	4277	2022-03-05T00:00:00Z
A. caviae	BQVQ01	SAMD00432567	PRJDB11802	GCA_022836095.1	KAM473	Contig	4.51	61.2	467	4010	2022-03-05T00:00:00Z
A. caviae	BQVR01	SAMD00432568	PRJDB11802	GCA_022836115.1	KAM474	Contig	4.69	60.9	488	4214	2022-03-05T00:00:00Z
A. caviae	BQVS01	SAMD00432569	PRJDB11802	GCA_022836135.1	KAM475	Contig	4.72	61.0	497	4192	2022-03-05T00:00:00Z
A. caviae	BQVT01	SAMD00432570	PRJDB11802	GCA_022836155.1	KAM476	Contig	4.68	60.9	528	4203	2022-03-05T00:00:00Z
A. caviae	BQVU01	SAMD00432571	PRJDB11802	GCA_022836175.1	KAM477	Contig	4.87	60.8	672	4389	2022-03-05T00:00:00Z
A. caviae	BQVV01	SAMD00432572	PRJDB11802	GCA_022836195.1	KAM478	Contig	4.73	60.9	490	4245	2022-03-05T00:00:00Z
A. caviae	BQVW01	SAMD00432573	PRJDB11802	GCA_022836215.1	KAM479	Contig	4.63	61.2	363	4161	2022-03-05T00:00:00Z
A. caviae	BQVX01	SAMD00432574	PRJDB11802	GCA_022836235.1	KAM480	Contig	4.72	60.8	575	4276	2022-03-05T00:00:00Z
A. caviae	BQVY01	SAMD00432575	PRJDB11802	GCA_022836255.1	KAM481	Contig	4.63	61.3	349	4232	2022-03-05T00:00:00Z
A. caviae	BQVZ01	SAMD00432576	PRJDB11802	GCA_022836275.1	KAM482	Contig	4.72	60.9	494	4237	2022-03-05T00:00:00Z
A. caviae	BQWA01	SAMD00432577	PRJDB11802	GCA_022836295.1	KAM483	Contig	4.52	61.2	470	4010	2022-03-05T00:00:00Z
A. caviae	BQWB01	SAMD00432578	PRJDB11802	GCA_022836315.1	KAM484	Contig	4.71	61.0	477	4182	2022-03-05T00:00:00Z
A. caviae	BQWC01	SAMD00432579	PRJDB11802	GCA_022836335.1	KAM485	Contig	4.69	60.9	473	4210	2022-03-05T00:00:00Z
A. caviae	BQWD01	SAMD00432580	PRJDB11802	GCA_022836355.1	KAM486	Contig	4.80	61.3	230	4394	2022-03-05T00:00:00Z
A. caviae	CAAKNG01	SAMEA5282775	PRJEB31025	GCA_901202955.1	Sch29	Contig	4.43	61.3	195	3970	2019-05-08T00:00:00Z
A. caviae	CAAKNI01	SAMEA5282776	PRJEB31025	GCA_901212305.1	Sch3N	Contig	4.74	61.4	192	4341	2019-05-08T00:00:00Z
A. caviae	CABMOA01	SAMEA5852032	PRJEB33885	GCA_902388085.1	MGYG-HGUT-02527	Contig	4.48	61.8	34	4006	2019-08-12T00:00:00Z
A. caviae	CACP01	SAMEA2272404	PRJEA51677	GCA_000208825.1	Ae398	Contig	4.44	61.4	149	4015	2010-11-23T00:00:00Z
A. caviae	JABSNY01	SAMN14752501	PRJNA628854	GCA_013282845.1	TW2	Contig	4.60	61.4	108	4065	2020-06-06T00:00:00Z
A. caviae	JAEHHN01	SAMN17083347	PRJNA685342	GCA_016729625.1	CN17A0069	Contig	4.49	61.1	139	4011	2021-01-20T00:00:00Z
A. caviae	JAEHHR01	SAMN17083343	PRJNA685342	GCA_016729725.1	CN17A0056	Contig	4.42	61.8	36	3905	2021-01-20T00:00:00Z
A. caviae	JAEHHY01	SAMN17083336	PRJNA685342	GCA_016729825.1	CN17A0038	Contig	4.44	61.8	49	3973	2021-01-20T00:00:00Z
A. caviae	JAEHIC01	SAMN17083332	PRJNA685342	GCA_016729905.1	CN17A0028	Contig	4.42	61.8	61	3987	2021-01-20T00:00:00Z
A. caviae	JAEHID01	SAMN17083331	PRJNA685342	GCA_016729935.1	CN17A0027	Contig	4.45	61.7	94	3943	2021-01-20T00:00:00Z
A. caviae	JAEHIE01	SAMN17083330	PRJNA685342	GCA_016729965.1	CN17A0024	Contig	4.40	62.0	21	3955	2021-01-20T00:00:00Z
A. caviae	JAEHII01	SAMN17083324	PRJNA685342	GCA_016730085.1	CN17A0007	Contig	4.31	61.9	28	3848	2021-01-20T00:00:00Z
A. caviae	JAEHIJ01	SAMN17083323	PRJNA685342	GCA_016730095.1	CN17A0006	Contig	4.58	61.0	199	4118	2021-01-20T00:00:00Z
A. caviae	JAEHIL01	SAMN17083383	PRJNA685342	GCA_016728885.1	CN17A0191	Contig	4.35	61.6	154	3874	2021-01-20T00:00:00Z
A. caviae	JAEHIM01	SAMN17083382	PRJNA685342	GCA_016728905.1	CN17A0183	Contig	4.38	61.6	183	3889	2021-01-20T00:00:00Z
A. caviae	JAEHIO01	SAMN17083380	PRJNA685342	GCA_016728955.1	CN17A0173	Contig	4.36	61.6	62	3913	2021-01-20T00:00:00Z
A. caviae	JAEHIP01	SAMN17083379	PRJNA685342	GCA_016729005.1	CN17A0166	Contig	4.32	62.0	18	3843	2021-01-20T00:00:00Z
A. caviae	JAEHIR01	SAMN17083377	PRJNA685342	GCA_016729055.1	CN17A0158	Contig	4.53	61.3	92	4014	2021-01-20T00:00:00Z
A. caviae	JAEHIW01	SAMN17083372	PRJNA685342	GCA_016729105.1	CN17A0126	Contig	4.57	61.3	133	4145	2021-01-20T00:00:00Z
A. caviae	JAEHIX01	SAMN17083371	PRJNA685342	GCA_016729135.1	CN17A0125	Contig	4.56	61.3	34	4062	2021-01-20T00:00:00Z
A. caviae	JAEHJB01	SAMN17083366	PRJNA685342	GCA_016729255.1	CN17A0118	Contig	4.48	61.5	93	4027	2021-01-20T00:00:00Z
A. caviae	JAEHJE01	SAMN17083363	PRJNA685342	GCA_016729305.1	CN17A0110	Contig	4.59	61.8	24	4098	2021-01-20T00:00:00Z
A. caviae	JAEHJL01	SAMN17083356	PRJNA685342	GCA_016729435.1	CN17A0095	Contig	4.31	61.8	83	3834	2021-01-20T00:00:00Z
A. caviae	JAEHJN01	SAMN17083354	PRJNA685342	GCA_016729465.1	CN17A0091	Contig	4.45	61.6	123	4012	2021-01-20T00:00:00Z
A. caviae	JAEHJP01	SAMN17083352	PRJNA685342	GCA_016729505.1	CN17A0086	Contig	4.40	62.0	25	3933	2021-01-20T00:00:00Z
A. caviae	JAEHJR01	SAMN17083350	PRJNA685342	GCA_016729565.1	CN17A0082	Contig	4.36	61.9	20	3907	2021-01-20T00:00:00Z
A. caviae	JAGDEN01	SAMN17101836	PRJNA685948	GCA_018360005.1	CECT 838	Contig	4.48	61.8	51	3984	2021-05-14T00:00:00Z
A. caviae	JAGDEU01	SAMN17101843	PRJNA685948	GCA_018359825.1	549_SP	Contig	4.50	61.2	79	4027	2021-05-14T00:00:00Z
A. caviae	JAGDEV01	SAMN17101844	PRJNA685948	GCA_018359875.1	550_SP	Contig	4.52	61.7	47	4046	2021-05-14T00:00:00Z
A. caviae	JAGDEX01	SAMN17101846	PRJNA685948	GCA_018359805.1	552_SP	Contig	4.46	61.7	41	4011	2021-05-14T00:00:00Z
A. caviae	JOVP01	SAMN02870964	PRJNA242225	GCA_000721855.1	YL12	Contig	4.85	61.5	30	4212	2014-07-02T00:00:00Z
A. caviae	JTBG02	SAMN02934528	PRJNA231221	GCA_000783695.2	FDAARGOS_76	Contig	4.88	61.0	10	4343	2014-11-14T00:00:00Z
A. caviae	JTBH02	SAMN02934527	PRJNA231221	GCA_000783715.2	FDAARGOS_75	Contig	4.56	61.7	1	4061	2014-11-14T00:00:00Z
A. caviae	JWJP01	SAMN03265383	PRJNA270276	GCA_000813475.1	L12	Contig	4.38	61.7	91	3941	2015-01-02T00:00:00Z
A. caviae	LIIX01	SAMN03995874	PRJNA292995	GCA_001270765.1	429865	Contig	4.70	61.0	4	4147	2015-08-19T00:00:00Z
A. caviae	LXJO01	SAMN04914380	PRJNA320016	GCA_001702475.1	VBF856	Contig	4.50	61.4	216	3527	2016-08-10T00:00:00Z
A. caviae	NXBW01	SAMN07680230	PRJNA408193	GCA_003265605.1	ZJ33-3	Contig	4.48	61.5	223	3994	2018-06-26T00:00:00Z
A. caviae	OVEM01	SAMEA4644083	PRJEB26597	GCA_900407325.1	KLMC2	Contig	4.52	61.0	746	3998	2018-05-09T00:00:00Z
A. caviae	OVEN01	SAMEA4644084	PRJEB26597	GCA_900407335.1	KLMC4	Contig	4.62	61.4	768	4201	2018-05-09T00:00:00Z
A. caviae	PUTR01	SAMN08519207	PRJNA433857	GCA_003322775.1	AFG_SD01_1510_Aca_0	Contig	4.50	61.1	228	3996	2018-07-15T00:00:00Z
A. caviae	QKOS01	SAMN09289756	PRJNA472583	GCA_004024495.1	GEO_48_Eff_A	Contig	4.53	61.7	253	4100	2019-01-15T00:00:00Z
A. caviae	RQJZ01	SAMN10457757	PRJNA506284	GCA_003849745.1	Aer593	Contig	4.31	61.8	243	3855	2018-11-28T00:00:00Z
A. caviae	UETO01	SAMEA4743709	PRJEB27351	GCA_900491665.1	ZJ33-3	Contig	4.48	61.5	223	4031	2018-06-24T00:00:00Z
A. caviae	WUTZ01	SAMN13683804	PRJNA597794	GCA_009831085.1	ScAc2001	Contig	4.49	61.1	340	3966	2020-01-06T00:00:00Z
A. dhakensis	AOBN01	SAMN02471842	PRJNA183198	GCA_000354675.1	173	Contig	4.79	61.6	74	4256	2013-04-06T00:00:00Z
A. dhakensis	AOBQ01	SAMN02471843	PRJNA183202	GCA_000354715.1	277	Contig	4.79	61.6	41	4329	2013-04-06T00:00:00Z
A. dhakensis	BAFL01	SAMD00036618	PRJDB70	GCA_000315195.1	AAK1	Contig	4.77	61.8	37	4225	2012-04-17T00:00:00Z
A. dhakensis	JAACMW01	SAMN13338996	PRJNA590791	GCA_019348645.1	IF_2	Contig	4.73	61.9	17	4174	2021-07-26T00:00:00Z
A. dhakensis	JADPIC01	SAMN16824286	PRJNA679132	GCA_015666195.1	1P11S3	Contig	4.88	61.6	43	4362	2020-11-23T00:00:00Z
A. dhakensis	JAEHHU01	SAMN17083340	PRJNA685342	GCA_016729785.1	CN17A0050	Contig	4.77	61.7	24	4243	2021-01-20T00:00:00Z
A. dhakensis	JAEHIK01	SAMN17083384	PRJNA685342	GCA_016728895.1	CN17A0194	Contig	4.71	61.7	26	4184	2021-01-20T00:00:00Z
A. dhakensis	JAEHIQ01	SAMN17083378	PRJNA685342	GCA_016728985.1	CN17A0164	Contig	4.73	61.9	28	4231	2021-01-20T00:00:00Z
A. dhakensis	JAEHIY01	SAMN17083370	PRJNA685342	GCA_016729085.1	CN17A0124	Contig	4.84	61.3	90	4292	2021-01-20T00:00:00Z
A. dhakensis	JAEHJI01	SAMN17083359	PRJNA685342	GCA_016729375.1	CN17A0101	Contig	4.88	61.4	78	4315	2021-01-20T00:00:00Z
A. dhakensis	JAEHJJ01	SAMN17083358	PRJNA685342	GCA_016729405.1	CN17A0100	Contig	4.76	61.7	36	4226	2021-01-20T00:00:00Z
A. dhakensis	JAEHJT01	SAMN17083348	PRJNA685342	GCA_016729585.1	CN17A0075	Contig	4.75	61.7	21	4208	2021-01-20T00:00:00Z
A. dhakensis	JAEIJA01	SAMN17083329	PRJNA685342	GCA_016730005.1	CN17A0022	Contig	4.83	61.8	28	4349	2021-01-20T00:00:00Z
A. dhakensis	JAEIJB01	SAMN17083328	PRJNA685342	GCA_016729975.1	CN17A0014	Contig	4.72	61.9	24	4191	2021-01-20T00:00:00Z
A. dhakensis	JAFIMO01	SAMN18022885	PRJNA703740	GCA_018682365.1	Icb10mS2	Contig	5.09	61.7	51	4575	2021-06-02T00:00:00Z
A. dhakensis	JAGDET01	SAMN17101842	PRJNA685948	GCA_018359865.1	547_SP	Contig	4.81	61.7	38	4289	2021-05-14T00:00:00Z
A. dhakensis	JALGBK01	SAMN26920427	PRJNA819262	GCA_022836795.1	Igbk	Contig	4.82	61.6	209	4332	2022-04-08T00:00:00Z
A. dhakensis	JDWD01	SAMN02597481	PRJNA237907	GCA_000708085.1	SSU	Contig	4.88	61.6	271	4354	2014-06-16T00:00:00Z
A. dhakensis	LZFM01	SAMN04494559	PRJNA312130	GCA_001673685.1	F2S2-1	Contig	4.75	61.8	64	4192	2016-06-21T00:00:00Z

A. dhakensis	PJOL01	SAMN08173719	PRJNA422283	GCA_003989145.1	CAIM 1873	Contig	4.93	61.6	193	4459	2018-12-31T00:00:00Z
A. dhakensis	RQKD01	SAMN10457753	PRJNA506284	GCA_003849805.1	Aer283	Contig	4.74	61.9	182	4207	2018-11-28T00:00:00Z
A. dhakensis	WNLF01	SAMN13353935	PRJNA591201	GCA_018094645.1	Pi16.2MC	Contig	4.87	61.5	16	4286	2021-04-19T00:00:00Z
A. dhakensis	WNLH01	SAMN13354252	PRJNA591217	GCA_018094765.1	OnIF3	Contig	4.73	61.8	13	4206	2021-04-19T00:00:00Z
A. diversa	APVG01	SAMN02471398	PRJNA183611	GCA_000367845.1	2478-85	Contig	4.03	61.7	104	3657	2013-04-19T00:00:00Z
A. enteropelogenes	JAEHHW01	SAMN17083338	PRJNA685342	GCA_016729765.1	CN17A0048	Contig	4.39	59.8	29	3915	2021-01-20T00:00:00Z
A. enteropelogenes	JAEHIG01	SAMN17083326	PRJNA685342	GCA_016730045.1	CN17A0011	Contig	4.51	59.9	21	4016	2021-01-20T00:00:00Z
A. enteropelogenes	JMGO02	SAMN02732394	PRJNA245216	GCA_000687355.2	1999lcr	Contig	4.33	60.0	18	3839	2014-05-09T00:00:00Z
A. enteropelogenes	LDWG01	SAMN03744441	PRJNA277406	GCA_001038825.1	LK14	Contig	4.68	62.0	49	4191	2015-06-24T00:00:00Z
A. finlandensis	JRGK01	SAMN03023686	PRJNA260478	GCA_000764645.1	4287D	Contig	4.72	58.6	376	4,311	2014-10-08T00:00:00Z
A. hydrophila	AMQA01	SAMN02470915	PRJNA175471	GCA_000315835.1	SNUFPC-A8	Contig	4.97	60.8	41	4508	2012-11-29T00:00:00Z
A. hydrophila	ANPN01	SAMN02471861	PRJNA183196	GCA_000350405.1	116	Contig	4.68	62.0	45	4201	2013-04-02T00:00:00Z
A. hydrophila	AOBM01	SAMN02471854	PRJNA183195	GCA_000354655.1	14	Contig	4.67	62.0	75	4203	2013-04-06T00:00:00Z
A. hydrophila	AOBO01	SAMN02471855	PRJNA183199	GCA_000354635.1	187	Contig	4.78	61.6	59	4313	2013-04-06T00:00:00Z
A. hydrophila	AOBP01	SAMN02471868	PRJNA183201	GCA_000354695.1	259	Contig	4.70	61.7	80	4219	2013-04-06T00:00:00Z
A. hydrophila	BAXY01	SAMD00000402	PRJDB1739	GCA_000698205.1	Ae34	Contig	4.71	61.6	28	4019	2014-05-21T00:00:00Z
A. hydrophila	BEYT01	SAMD00097253	PRJDB6418	GCA_002897335.1	Ae25	Contig	4.76	61.3	45	NA	2017-12-07T00:00:00Z
A. hydrophila	BPOS01	SAMD00294598	PRJDB11802	GCA_019972695.1	KAM385	Contig	5.12	61.1	296	4686	2021-07-14T22:10:00Z
A. hydrophila	BQVF01	SAMD00432556	PRJDB11802	GCA_022835875.1	KAM461	Contig	4.93	61.3	107	4490	2022-03-05T00:00:00Z
A. hydrophila	CAAKNP01	SAMEA5282778	PRJEB31025	GCA_901212375.1	KLG1	Contig	4.91	61.2	154	4410	2019-05-08T00:00:00Z
A. hydrophila	CABMNZ01	SAMEA5852031	PRJEB33885	GCA_902388065.1	MGYG-HGUT-02526	Contig	5.05	61.0	2	4468	2019-08-12T00:00:00Z
A. hydrophila	CAHPSB01	SAMEA6863327	PRJEB38442	GCA_903684605.1	BB1457	Contig	4.93	61.5	196	4390	2022-02-17T00:00:00Z
A. hydrophila	JAANAW01	SAMN14278045	PRJNA610129	GCA_011603095.1	LZ-MG14	Contig	4.78	61.5	36	4272	2020-03-21T00:00:00Z
A. hydrophila	JABAJN01	SAMN14521489	PRJNA622569	GCA_012641195.1	RIT668	Contig	4.77	61.5	90	4281	2020-04-21T00:00:00Z
A. hydrophila	JACLAM01	SAMN15641156	PRJNA648413	GCA_014396945.1	B-2	Contig	5.22	60.9	4	4620	2020-09-02T00:00:00Z
A. hydrophila	JACLAN01	SAMN15641141	PRJNA648413	GCA_014396965.1	B-1	Contig	4.92	61.0	23	801	2020-09-02T00:00:00Z
A. hydrophila	JACLAO01	SAMN15641139	PRJNA648413	GCA_014397045.1	A-1	Contig	4.98	61.2	8	4390	2020-09-02T00:00:00Z
A. hydrophila	JAEHHP01	SAMN17083345	PRJNA685342	GCA_016729665.1	CN17A0062	Contig	4.72	61.5	18	4217	2021-01-20T00:00:00Z
A. hydrophila	JAEHHS01	SAMN17083342	PRJNA685342	GCA_016729685.1	CN17A0055	Contig	4.72	61.5	20	4220	2021-01-20T00:00:00Z
A. hydrophila	JAEHIT01	SAMN17083375	PRJNA685342	GCA_016729025.1	CN17A0136	Contig	4.94	61.1	49	4433	2021-01-20T00:00:00Z
A. hydrophila	JAEHIU01	SAMN17083374	PRJNA685342	GCA_016729115.1	CN17A0135	Contig	4.67	61.3	15	4143	2021-01-20T00:00:00Z
A. hydrophila	JAEHIV01	SAMN17083373	PRJNA685342	GCA_016729125.1	CN17A0134	Contig	4.71	61.6	24	4195	2021-01-20T00:00:00Z
A. hydrophila	JAEHJS01	SAMN17083349	PRJNA685342	GCA_016729605.1	CN17A0078	Contig	4.68	61.7	17	4143	2021-01-20T00:00:00Z
A. hydrophila subsp. *hydrophila*	JAFIMN01	SAMN18022882	PRJNA703737	GCA_018682315.1	Hla5mS2	Contig	4.97	61.2	108	4424	2021-06-02T00:00:00Z
A. hydrophila	JAGDEM01	SAMN17101835	PRJNA685948	GCA_018360025.1	ATCC 7966	Contig	4.68	61.6	31	4163	2021-05-14T00:00:00Z
A. hydrophila	JAIWZB01	SAMN21894389	PRJNA767215	GCA_020257365.1	LPL-1	Contig	5.05	60.8	36	4515	2021-10-04T00:00:00Z
A. hydrophila	JAJDSS01	SAMN21168047	PRJNA759416	GCA_020639395.1	71328	Contig	4.86	61.4	3	4298	2021-10-26T00:00:00Z
A. hydrophila	JDWB01	SAMN02597476	PRJNA237913	GCA_000708105.1	NF1	Contig	4.81	61.1	150	4317	2014-06-16T00:00:00Z
A. hydrophila	JDWC01	SAMN02597477	PRJNA237917	GCA_000708065.1	NF2	Contig	4.79	61.3	134	4314	2014-06-16T00:00:00Z
A. hydrophila	JEMK01	SAMN02643439	PRJNA183197	GCA_000586035.1	145	Contig	4.86	61.5	113	4308	2014-03-07T00:00:00Z
A. hydrophila	JEML01	SAMN02643440	PRJNA183200	GCA_000586055.1	226	Contig	5.11	60.9	135	4641	2014-03-07T00:00:00Z
A. hydrophila	JFJO01	SAMN02673382	PRJNA236257	GCA_000626755.1	AD9	Contig	4.91	61.3	221	4445	2014-04-11T00:00:00Z
A. hydrophila	JPEH01	SAMN02892980	PRJNA253773	GCA_000724965.1	RB-AH	Contig	5.09	60.8	3	4571	2014-07-11T00:00:00Z
A. hydrophila	JRWS01	SAMN03113148	PRJNA264123	GCA_000783255.1	M013	Contig	4.97	61.0	164	4494	2014-11-14T00:00:00Z
A. hydrophila	JSWA01	SAMN03162034	PRJNA265923	GCA_000786315.1	M023	Contig	4.91	60.9	183	4457	2014-11-23T00:00:00Z
A. hydrophila	JSXE01	SAMN03154072	PRJNA265924	GCA_000803185.1	M062	Contig	4.97	61.1	140	4551	2014-12-17T00:00:00Z
A. hydrophila	JTBD02	SAMN02934532	PRJNA231221	GCA_000783635.2	FDAARGOS_78	Contig	4.93	61.0	7	4290	2014-11-14T00:00:00Z
A. hydrophila	JWJQ01	SAMN03265385	PRJNA270277	GCA_000813465.1	L14f	Contig	4.68	61.7	70	4166	2015-01-02T00:00:00Z
A. hydrophila	LNUR01	SAMN04253181	PRJNA301651	GCA_001535585.1	TN-97-08	Contig	5.09	60.8	16	4586	2016-01-25T00:00:00Z
A. hydrophila	LRRV01	SAMN04274131	PRJNA302467	GCA_001604835.1	AL09-79	Contig	4.97	60.9	13	4420	2016-03-31T00:00:00Z
A. hydrophila	LRRW01	SAMN04273360	PRJNA302455	GCA_001604855.1	AL10-121	Contig	4.97	60.9	13	4429	2016-03-31T00:00:00Z
A. hydrophila	LRRX01	SAMN04274132	PRJNA302468	GCA_001604895.1	ML09-121	Contig	4.97	60.9	14	4423	2016-03-31T00:00:00Z
A. hydrophila	LRRY01	SAMN04274135	PRJNA302469	GCA_001604905.1	ML09-122	Contig	4.97	60.9	19	4425	2016-03-31T00:00:00Z
A. hydrophila	LXME01	SAMN04939662	PRJNA320461	GCA_001717755.1	2JBN101	Contig	5.09	60.8	47	4545	2016-08-31T00:00:00Z
A. hydrophila	LYXN01	SAMN05187193	PRJNA323709	GCA_001684445.1	AH-1	Contig	5.12	60.9	218	4719	2016-07-13T00:00:00Z
A. hydrophila	LYZF01	SAMN04967787	PRJNA321178	GCA_001705905.1	AL97-91	Contig	4.83	61.2	42	4363	2016-08-17T00:00:00Z
A. hydrophila	LYZG01	SAMN04967900	PRJNA321182	GCA_001705915.1	MN98-04	Contig	4.88	61.1	46	4415	2016-08-17T00:00:00Z
A. hydrophila	LYZH01	SAMN04967902	PRJNA321184	GCA_001705945.1	Arkansas 2010	Contig	4.97	60.9	12	4423	2016-08-17T00:00:00Z
A. hydrophila	MAKI01	SAMN05294042	PRJNA326907	GCA_001756325.1	M052	Contig	4.97	61.1	99	4506	2016-10-11T00:00:00Z
A. hydrophila	MAKJ01	SAMN05294043	PRJNA326908	GCA_001756345.1	M053	Contig	4.96	61.1	162	4513	2016-10-11T00:00:00Z
A. hydrophila	MTPO01	SAMN06233223	PRJNA361399	GCA_001982465.1	Ah-HSP	Contig	5.03	61.2	174	4517	2017-02-01T00:00:00Z
A. hydrophila	PQLL01	SAMN04286972	PRJNA430813	GCA_002918915.1	AHNIH2	Contig	5.36	60.8	137	4896	2018-02-06T00:00:00Z
A. hydrophila	PUTQ01	SAMN08519208	PRJNA433857	GCA_003323285.1	AFG_SD03_1510_Ahy_0	Contig	5.00	61.1	83	4487	2018-07-15T00:00:00Z
A. hydrophila	UFSL01	SAMEA4076704	PRJEB6403	GCA_900445175.1	NCTC8049	Contig	4.75	61.6	2	4158	2018-08-06T00:00:00Z
A. hydrophila	VHIW01	SAMN12097418	PRJNA528964	GCA_019742435.1	4484	Contig	4.83	61.2	13	2153	2021-08-24T00:00:00Z
A. hydrophila	VHIX01	SAMN12097414	PRJNA528964	GCA_019742455.1	2961	Contig	4.84	61.5	3	3947	2021-08-24T00:00:00Z
A. hydrophila	VWTU01	SAMN12706444	PRJNA564235	GCA_015353175.1	A34a	Contig	4.99	60.7	165	4514	2020-11-11T00:00:00Z
A. hydrophila	WNLG01	SAMN13354297	PRJNA591221	GCA_018094725.1	On9M	Contig	4.93	61.1	11	4388	2021-04-19T00:00:00Z
A. hydrophila	WOCB01	SAMN13387565	PRJNA591768	GCA_018094775.1	LaG38	Contig	4.75	61.5	13	4187	2021-04-19T00:00:00Z
A. hydrophila	WOCC01	SAMN13389033	PRJNA591795	GCA_018094805.1	OnP22	Contig	4.78	61.5	10	4207	2021-04-19T00:00:00Z
A. jandaei	JAACNC01	SAMN13343509	PRJNA590949	GCA_019348615.1	GT15	Contig	4.61	58.8	42	4147	2021-07-26T00:00:00Z
A. jandaei	JAEHIN01	SAMN17083381	PRJNA685342	GCA_016728915.1	CN17A0176	Contig	4.31	59.2	19	3855	2021-01-20T00:00:00Z
A. jandaei	JAEHJA01	SAMN17083367	PRJNA685342	GCA_016729225.1	CN17A0119	Contig	4.69	58.6	36	4236	2021-01-20T00:00:00Z
A. jandaei	JAEHJC01	SAMN17083365	PRJNA685342	GCA_016729205.1	CN17A0115	Contig	4.50	58.9	18	4031	2021-01-20T00:00:00Z
A. jandaei	JAEHJF01	SAMN17083362	PRJNA685342	GCA_016729325.1	CN17A0104	Contig	4.42	59.0	42	3958	2021-01-20T00:00:00Z
A. jandaei	JAEHJQ01	SAMN17083351	PRJNA685342	GCA_016729525.1	CN17A0084	Contig	4.51	58.9	25	4042	2021-01-20T00:00:00Z
A. jandaei	JFDL01	SAMN02597482	PRJNA237126	GCA_000708125.1	Riv2	Contig	4.48	59.0	43	4042	2014-06-16T00:00:00Z
A. jandaei	JWJR01	SAMN03265386	PRJNA270278	GCA_000813485.1	L14h	Contig	4.68	61.7	114	4187	2015-01-02T00:00:00Z
A. jandaei	RPDF01	SAMN10395257	PRJNA504557	GCA_009749285.1	82	Contig	4.76	58.5	481	4339	2019-12-13T00:00:00Z
A. jandaei	RQKB01	SAMN10457755	PRJNA506284	GCA_003849685.1	Aer337	Contig	4.71	58.6	101	4306	2018-11-28T00:00:00Z
A. lacus	JRGM01	SAMN03023876	PRJNA260478	GCA_000764665.1	AE122		4.39437	59.0	196	3994	2014-10-08T00:00:00Z
A. media	CAAKNK01	SAMEA5282781	PRJEB31025	GCA_901212365.1	KLG6	Contig	4.54	61.1	454	3961	2019-05-08T00:00:00Z
A. media	CALUBH01	SAMEA110089378	PRJEB47281	GCA_943914245.1	8t8T2iAkVV_bin.10.MAG	Contig	4.49	61.8	73	NA	2022-06-13T00:00:00Z
A. media	JAEHIH01	SAMN17083325	PRJNA685342	GCA_016730065.1	CN17A0010	Contig	4.56	62.3	18	4025	2021-01-20T00:00:00Z
A. media	JAGDEO01	SAMN17101837	PRJNA685948	GCA_018359985.1	ATCC 33907	Contig	4.47	61.2	199	4001	2021-05-14T00:00:00Z
A. media	JAGDES01	SAMN17101841	PRJNA685948	GCA_018359915.1	D180	Contig	4.50	61.8	52	3936	2021-05-14T00:00:00Z
A. media	JRBF01	SAMN03018686	PRJNA260228	GCA_000754915.1	ARB13	Contig	4.61	61.0	180	4124	2014-09-16T00:00:00Z
A. media	JRBG01	SAMN03018687	PRJNA260227	GCA_000754925.1	ARB20	Contig	4.62	61.0	185	4126	2014-09-16T00:00:00Z
A. molluscorum	AQGQ01	SAMN02471397	PRJNA183610	GCA_000388115.1	848		4.24	59.2	309	3786	2013-05-03T00:00:00Z
A. piscicola	LYXO01	SAMN05187196	PRJNA323710	GCA_001684435.1	AH-3	Contig	4.89	59.8	82	4221	2016-07-13T00:00:00Z
A. popoffii	JAGRZL01	SAMN18685274	PRJNA720925	GCA_018138755.1	ID682	Contig	4.68	58.6	128	4151	2021-04-24T00:00:00Z

A. rivipollensis	JAAIKZ01	SAMN14091307	PRJNA606446	GCA_010974815.1	G87	Contig	4.66	61.1	71	4118	2020-02-23T00:00:00Z
A. rivipollensis	JAAILA01	SAMN14091306	PRJNA606446	GCA_010974825.1	G78	Contig	4.53	61.5	57	4045	2020-02-23T00:00:00Z
A. rivipollensis	JAAILC01	SAMN14091304	PRJNA606446	GCA_010974925.1	G36	Contig	4.53	61.5	93	4057	2020-02-23T00:00:00Z
A. salmonicida subsp. *achromo*	AMQG02	SAMN02470909	PRJNA175472	GCA_000315855.2	AS03	Contig	4.96	58.3	69	4158	2012-11-29T00:00:00Z
A. salmonicida subsp. *pectinol*	ARYZ02	SAMN03322533	PRJNA198485	GCA_000447435.2	34mel	Contig	4.77	58.5	253	4246	2013-08-15T00:00:00Z
A. salmonicida subsp. *masouc*	BAWQ01	SAMD00000014	PRJDB242	GCA_000647955.1	NBRC 13784	Contig	4.50	58.8	227	3908	2014-04-15T20:32:00Z
A. salmonicida	CAACYG01	SAMEA104338356	PRJEB6403	GCA_900683655.1	3012STDY7122732	Contig	4.83	58.5	22	4281	2019-02-20T00:00:00Z
A. salmonicida subsp. *salmoni*	JADKRF01	SAMN16663060	PRJNA264317	GCA_016918885.1	BBCC2887	Contig	4.76	58.5	116	4263	2021-02-22T00:00:00Z
A. salmonicida subsp. *salmoni*	JAFBAV01	SAMN17709026	PRJNA264317	GCA_016811055.1	2004-072	Contig	4.75	58.5	117	4245	2021-02-06T00:00:00Z
A. salmonicida	JAGUQQ01	SAMN18904053	PRJNA725799	GCA_018274765.1	JAOP-5	Contig	5.01	58.6	159	4507	2021-05-04T00:00:00Z
A. salmonicida subsp. *salmoni*	JALKVL01	SAMN27544343	PRJNA698804	GCA_023108375.1	VI-88/09/03175	Contig	4.74	58.5	123	4204	2022-04-25T00:00:00Z
A. salmonicida subsp. *salmoni*	JRYV01	SAMN03120844	PRJNA264317	GCA_000786795.1	2009-144K3	Contig	4.96	58.3	214	4442	2014-11-25T00:00:00Z
A. salmonicida subsp. *salmoni*	JRYW01	SAMN03120845	PRJNA264317	GCA_000786805.1	2004-05MF26	Contig	5.02	58.3	126	4601	2014-11-25T00:00:00Z
A. salmonicida	JZTG01	SAMN03395036	PRJNA264317	GCA_001466435.1	Y567	Contig	4.55	58.9	47	4072	2015-12-15T00:00:00Z
A. salmonicida subsp. *smithia*	JZTI01	SAMN03396265	PRJNA264317	GCA_001466445.1	JF4097	Contig	4.31	58.7	344	3948	2015-12-15T00:00:00Z
A. salmonicida subsp. *salmoni*	LAIS01	SAMN03421178	PRJNA264317	GCA_002110585.1	M15879-11	Contig	5.03	58.1	916	4553	2017-04-26T00:00:00Z
A. salmonicida subsp. *salmoni*	LAIT01	SAMN03421189	PRJNA264317	GCA_005476635.1	M16474-11	Contig	4.96	58.4	115	4421	2019-05-17T00:00:00Z
A. salmonicida subsp. *salmoni*	LMTK01	SAMN04226662	PRJNA264317	GCA_001902165.1	09-0167	Contig	4.89	58.4	206	4275	2016-12-13T00:00:00Z
A. salmonicida subsp. *salmoni*	LMTT01	SAMN04219606	PRJNA264317	GCA_002811185.1	M22710-11	Contig	4.86	58.4	116	4411	2017-12-05T00:00:00Z
A. salmonicida subsp. *salmoni*	LNGB01	SAMN04272881	PRJNA264317	GCA_004151085.1	m11743-09	Contig	4.86	58.4	119	4412	2019-02-08T00:00:00Z
A. salmonicida subsp. *salmoni*	LSGW01	SAMN04449999	PRJNA310296	GCA_001643305.1	ATCC 33658	Contig	4.73	58.5	119	4165	2016-05-13T00:00:00Z
A. salmonicida subsp. *salmoni*	LSGX01	SAMN04450000	PRJNA310296	GCA_001643355.1	J227	Contig	4.70	58.5	122	4182	2016-05-13T00:00:00Z
A. salmonicida subsp. *salmoni*	LSGY01	SAMN04450001	PRJNA310296	GCA_001643285.1	J231	Contig	4.75	58.4	143	4252	2016-05-13T00:00:00Z
A. salmonicida subsp. *salmoni*	MIIM01	SAMN05728463	PRJNA264317	GCA_001901975.1	01-B522	Contig	5.17	58.1	233	4376	2016-12-13T00:00:00Z
A. salmonicida subsp. *salmoni*	MIIN01	SAMN05728464	PRJNA264317	GCA_001901965.1	170-68	Contig	4.82	58.4	115	4281	2016-12-13T00:00:00Z
A. salmonicida subsp. *salmoni*	MIIO01	SAMN05728465	PRJNA264317	GCA_001901985.1	JF2267	Contig	5.06	58.2	211	4232	2016-12-13T00:00:00Z
A. salmonicida subsp. *salmoni*	MIIP01	SAMN05728466	PRJNA264317	GCA_001902025.1	m23067-09	Contig	4.92	58.4	215	4112	2016-12-13T00:00:00Z
A. salmonicida subsp. *salmoni*	MIIQ01	SAMN05728467	PRJNA264317	GCA_001902045.1	2009-157 K5	Contig	4.87	58.4	112	4334	2016-12-13T00:00:00Z
A. salmonicida subsp. *salmoni*	MIIR01	SAMN05728468	PRJNA264317	GCA_001902055.1	2010-47 K18	Contig	4.90	58.4	116	4370	2016-12-13T00:00:00Z
A. salmonicida subsp. *salmoni*	MIIS01	SAMN05728470	PRJNA264317	GCA_001902065.1	JF2506	Contig	4.78	58.4	117	4217	2016-12-13T00:00:00Z
A. salmonicida subsp. *salmoni*	MIIT01	SAMN05728471	PRJNA264317	GCA_001902105.1	JF2507	Contig	4.77	58.5	109	4216	2016-12-13T00:00:00Z
A. salmonicida subsp. *salmoni*	MIIU01	SAMN05728472	PRJNA264317	GCA_001902125.1	JF3517	Contig	4.94	58.3	132	4388	2016-12-13T00:00:00Z
A. salmonicida	NQMJ01	SAMN07491057	PRJNA264317	GCA_002883135.1	M18076-11	Contig	4.43	58.7	228	4014	2018-01-16T00:00:00Z
A. salmonicida	NVQH01	SAMN07450651	PRJNA389557	GCA_002317045.1	ECFood+05	Contig	4.71	58.8	58	4161	2017-09-20T00:00:00Z
A. salmonicida	PSZI01	SAMN08524353	PRJNA264317	GCA_003947355.1	AJ83	Contig	4.80	58.3	47	4272	2018-12-14T00:00:00Z
A. salmonicida	PSZJ01	SAMN08524352	PRJNA264317	GCA_003947375.1	A308	Contig	4.97	58.2	110	4496	2018-12-14T00:00:00Z
A. salmonicida	UAPT01	SAMEA4530645	PRJEB6403	GCA_900445115.1	NCTC12959	Contig	4.93	58.3	10	4294	2018-06-12T00:00:00Z
A. salmonicida	UFSF01	SAMEA80462668	PRJEB6403	GCA_900445125.1	NCTC10402	Contig	4.87	58.4	7	4169	2018-08-06T00:00:00Z
A. salmonicida subsp. *achromo*	VCSA01	SAMN11836207	PRJNA264317	GCA_006044075.1	23055	Contig	4.42	58.7	305	3835	2019-06-05T00:00:00Z
A. salmonicida subsp. *achromo*	VCSB01	SAMN11836206	PRJNA264317	GCA_006044015.1	23053	Contig	4.42	58.7	308	3835	2019-06-05T00:00:00Z
A. salmonicida subsp. *achromo*	VCSC01	SAMN11836205	PRJNA264317	GCA_006043955.1	23051	Contig	4.42	58.7	304	3835	2019-06-05T00:00:00Z
A. salmonicida subsp. *achromo*	VCSD01	SAMN11836204	PRJNA264317	GCA_006044035.1	23056	Contig	4.37	58.7	297	3783	2019-06-05T00:00:00Z
A. salmonicida	VOIP01	SAMN12399690	PRJNA264317	GCA_009725185.1	JF2480	Contig	4.84	58.6	101	4345	2019-12-02T00:00:00Z
A. schubertii	JAIRBS01	SAMN21302987	PRJNA761361	GCA_020089835.1	CHULA2021b	Contig	4.23	61.9	41	3905	2021-09-21T00:00:00Z
A. schubertii	JAIRBT01	SAMN21302986	PRJNA761361	GCA_020089825.1	CHULA2021a	Contig	4.28	61.6	87	3953	2021-09-21T00:00:00Z
A. sobria	JAGDEP01	SAMN17101838	PRJNA685948	GCA_018359945.1	CIP 74.33	Contig	4.69	57.6	73	4161	2021-05-14T00:00:00Z
A. sobria	LJZX01	SAMN04100937	PRJNA296590	GCA_002843345.1	JF2635	Contig	4.80	57.8	121	4400	2017-12-15T00:00:00Z
A. sobria	NKWB01	SAMN07312777	PRJNA391781	GCA_006243235.1	PAQ091014-19	Contig	4.68	57.7	112	4182	2019-06-11T00:00:00Z
A. sobria	NQML01	SAMN07490922	PRJNA397895	GCA_002843445.1	TM12	Contig	4.54	57.8	98	4014	2017-12-15T00:00:00Z
A. sobria	NQMM01	SAMN07491056	PRJNA397895	GCA_002843435.1	TM18	Contig	4.70	57.7	101	4196	2017-12-15T00:00:00Z
A. sobria	VEMU01	SAMN11952138	PRJNA546108	GCA_008086875.1	CHT-30	Contig	4.91	57.7	343	4470	2019-08-26T00:00:00Z
A. taiwanensis	BAWK01	SAMD00000007	PRJDB1639	GCA_000699185.1	LMG 24683	Contig	4.23	62.8	104	3715	2014-05-22T20:31:00Z
A. taiwanensis	QORJ01	SAMN09519167	PRJNA478520	GCA_004524015.1	186	Contig	4.77	61.5	118	4281	2019-03-29T00:00:00Z
A. taiwanensis	QORK01	SAMN09519166	PRJNA478520	GCA_004524025.1	198	Contig	4.74	61.6	130	4252	2019-03-29T00:00:00Z
A. taiwanensis	QORL01	SAMN09519165	PRJNA478520	GCA_004524045.1	1713	Contig	4.74	61.6	130	4252	2019-03-29T00:00:00Z
A. veronii Phln2	ANNT01	SAMN02471783	PRJNA169110	GCA_000409545.1	Phln2	Contig	4.30	58.8	1899	NA	2013-06-10T00:00:00Z
A. veronii	BDGY01	SAMD00057823	PRJDB5119	GCA_001748325.1	Ae52	Contig	4.56	58.7	80	NA	2016-08-24T20:43:00Z
A. veronii	CAAKNH01	SAMEA5282777	PRJEB31025	GCA_901212295.1	BC88	Contig	4.60	58.5	155	4079	2019-05-08T00:00:00Z
A. veronii	CAAKNJ01	SAMEA5282782	PRJEB31025	GCA_901212345.1	KLG7	Contig	4.55	58.8	104	4076	2019-05-08T00:00:00Z
A. veronii	CAAKNL01	SAMEA5282780	PRJEB31025	GCA_901212355.1	KLG5	Contig	4.74	58.5	103	4270	2019-05-08T00:00:00Z
A. veronii	CAAKNM01	SAMEA5282783	PRJEB31025	GCA_901212395.1	KLG8	Contig	4.59	58.6	76	4143	2019-05-08T00:00:00Z
A. veronii	CAAKNN01	SAMEA5282784	PRJEB31025	GCA_901212405.1	KLG9	Contig	4.61	58.7	74	4149	2019-05-08T00:00:00Z
A. veronii	CABLBN01	SAMEA5851195	PRJEB33885	GCA_902377445.1	MGYG-HGUT-01691	Contig	4.54	58.5	24	4084	2019-08-12T00:00:00Z
A. veronii	CAJKQS01	SAMEA7846589	PRJEB37358	GCA_905202105.1	ERR1305902-bin.15	Contig	4.11	59.4	226	NA	2021-02-01T00:00:00Z
A. veronii	CDDU01	SAMEA2752401	PRJEB7047	GCA_000820285.1	CIP 107763	Contig	4.43	58.8	64	3989	2014-11-24T00:00:00Z
A. veronii	JAACND01	SAMN13344001	PRJNA590969	GCA_019348735.1	Pi 23.1 MC	Contig	4.81	58.4	35	4284	2021-07-26T00:00:00Z
A. veronii	JAAQQM01	SAMN14381339	PRJNA612772	GCA_012029595.1	UDRT09	Contig	4.61	58.5	186	4080	2020-04-03T00:00:00Z
A. veronii	JAAQQN01	SAMN14381366	PRJNA612772	GCA_012029535.1	CNRT12	Contig	4.90	58.1	479	4448	2020-04-03T00:00:00Z
A. veronii	JAAQQO01	SAMN14381434	PRJNA612772	GCA_012029545.1	NK01	Contig	4.56	58.5	95	4022	2020-04-03T00:00:00Z
A. veronii	JAAQQP01	SAMN14381462	PRJNA612772	GCA_012029575.1	NK02	Contig	4.80	58.2	400	4372	2020-04-03T00:00:00Z
A. veronii	JAAQQQ01	SAMN14381700	PRJNA612772	GCA_012029585.1	NK07	Contig	4.78	58.6	46	4292	2020-04-03T00:00:00Z
A. veronii	JACEGL01	SAMN15587301	PRJNA525849	GCA_013697145.1	C198	Contig	4.58	58.6	3	3995	2020-07-26T00:00:00Z
A. veronii	JAEHHO01	SAMN17083346	PRJNA685342	GCA_016729595.1	CN17A0067	Contig	4.55	58.7	60	3997	2021-01-20T00:00:00Z
A. veronii	JAEHHQ01	SAMN17083344	PRJNA685342	GCA_016729675.1	CN17A0059	Contig	4.26	58.9	48	3765	2021-01-20T00:00:00Z
A. veronii	JAEHHT01	SAMN17083341	PRJNA685342	GCA_016729735.1	CN17A0054	Contig	4.33	58.9	57	3811	2021-01-20T00:00:00Z
A. veronii	JAEHHV01	SAMN17083339	PRJNA685342	GCA_016729775.1	CN17A0049	Contig	4.30	58.9	31	3767	2021-01-20T00:00:00Z
A. veronii	JAEHHX01	SAMN17083337	PRJNA685342	GCA_016729835.1	CN17A0040	Contig	4.44	58.9	32	3896	2021-01-20T00:00:00Z
A. veronii	JAEHHZ01	SAMN17083335	PRJNA685342	GCA_016729865.1	CN17A0036	Contig	4.45	58.9	38	3993	2021-01-20T00:00:00Z
A. veronii	JAEHIA01	SAMN17083334	PRJNA685342	GCA_016729885.1	CN17A0031	Contig	4.42	58.9	45	3890	2021-01-20T00:00:00Z
A. veronii	JAEHIB01	SAMN17083333	PRJNA685342	GCA_016729895.1	CN17A0029	Contig	4.60	58.8	19	4101	2021-01-20T00:00:00Z
A. veronii	JAEHIF01	SAMN17083327	PRJNA685342	GCA_016729985.1	CN17A0013	Contig	4.45	58.9	49	3981	2021-01-20T00:00:00Z
A. veronii	JAEHIS01	SAMN17083376	PRJNA685342	GCA_016728995.1	CN17A0154	Contig	4.44	59.0	33	3938	2021-01-20T00:00:00Z
A. veronii	JAEHIZ01	SAMN17083369	PRJNA685342	GCA_016729185.1	CN17A0122	Contig	4.48	58.7	33	3964	2021-01-20T00:00:00Z
A. veronii	JAEHJD01	SAMN17083364	PRJNA685342	GCA_016729285.1	CN17A0114	Contig	4.43	58.9	35	3966	2021-01-20T00:00:00Z
A. veronii	JAEHJG01	SAMN17083361	PRJNA685342	GCA_016729355.1	CN17A0103	Contig	4.43	58.8	102	3892	2021-01-20T00:00:00Z
A. veronii	JAEHJH01	SAMN17083360	PRJNA685342	GCA_016729345.1	CN17A0102	Contig	4.47	58.7	75	3936	2021-01-20T00:00:00Z
A. veronii	JAEHJK01	SAMN17083357	PRJNA685342	GCA_016729425.1	CN17A0097	Contig	4.52	58.6	88	4004	2021-01-20T00:00:00Z
A. veronii	JAEHJM01	SAMN17083355	PRJNA685342	GCA_016729485.1	CN17A0093	Contig	4.35	58.6	64	3831	2021-01-20T00:00:00Z
A. veronii	JAEHJO01	SAMN17083353	PRJNA685342	GCA_016729495.1	CN17A0087	Contig	4.58	58.6	80	4069	2021-01-20T00:00:00Z
A. veronii	JAEHOJ01	SAMN17083368	PRJNA685342	GCA_016729245.1	CN17A0120	Contig	4.52	58.6	128	4045	2021-01-20T00:00:00Z
A. veronii	JAGDEQ01	SAMN17101839	PRJNA685948	GCA_018359905.1	ATCC 35624	Contig	4.51	58.9	40	4050	2021-05-14T00:00:00Z

A. veronii	JAGDEW01	SAMN17101845	PRJNA685948	GCA_018359845.1	551_SP	Contig	4.57	58.8	92	4103	2021-05-14T00:00:00Z
A. veronii	JAGDEY01	SAMN17101847	PRJNA685948	GCA_018359765.1	553_SP	Contig	4.60	58.7	49	4092	2021-05-14T00:00:00Z
A. veronii	JAHVAR01	SAMN20179697	PRJNA746069	GCA_022875935.1	HD6451	Contig	4.92	58.7	216	4482	2022-04-11T00:00:00Z
A. veronii	JAIEXX01	SAMN20667228	PRJNA749892	GCA_021608665.1	A20	Contig	4.67	58.7	42	4222	2022-01-25T00:00:00Z
A. veronii	JAIEXY01	SAMN20667229	PRJNA749892	GCA_021608705.1	A21	Contig	4.49	58.8	80	3967	2022-01-25T00:00:00Z
A. veronii	JAIEXZ01	SAMN20667230	PRJNA749892	GCA_021608675.1	A26	Contig	4.52	58.8	34	4016	2022-01-25T00:00:00Z
A. veronii	JAIEYA01	SAMN20667231	PRJNA749892	GCA_021608715.1	A27	Contig	4.47	58.7	57	3973	2022-01-25T00:00:00Z
A. veronii	JAIEYB01	SAMN20667232	PRJNA749892	GCA_021608645.1	A7	Contig	4.52	58.8	80	3989	2022-01-25T00:00:00Z
A. veronii	JAIEYC01	SAMN20667233	PRJNA749892	GCA_021608615.1	A8	Contig	4.42	58.8	60	3917	2022-01-25T00:00:00Z
A. veronii	JAIEYD01	SAMN20667234	PRJNA749892	GCA_021608575.1	A9	Contig	4.54	58.8	51	4075	2022-01-25T00:00:00Z
A. veronii	JAIEYE01	SAMN20667235	PRJNA749892	GCA_021608545.1	A20-10	Contig	4.51	58.6	36	3997	2022-01-25T00:00:00Z
A. veronii	JAIEYF01	SAMN20667236	PRJNA749892	GCA_021608585.1	A20-12	Contig	4.46	58.9	56	3978	2022-01-25T00:00:00Z
A. veronii	JAIEYG01	SAMN20667237	PRJNA749892	GCA_021608525.1	A20-14	Contig	4.59	58.5	76	4093	2022-01-25T00:00:00Z
A. veronii	JAIEYH01	SAMN20667238	PRJNA749892	GCA_021608505.1	A20-17	Contig	4.46	58.9	70	3971	2022-01-25T00:00:00Z
A. veronii	JAIEYI01	SAMN20667239	PRJNA749892	GCA_021608565.1	A20-5	Contig	4.50	58.8	40	3978	2022-01-25T00:00:00Z
A. veronii	JAIEYJ01	SAMN20667240	PRJNA749892	GCA_021608465.1	A20-8	Contig	4.40	59.0	40	3863	2022-01-25T00:00:00Z
A. veronii	JAIEYK01	SAMN20667241	PRJNA749892	GCA_021608445.1	A21-10	Contig	4.42	58.6	57	3914	2022-01-25T00:00:00Z
A. veronii	JAIEYL01	SAMN20667242	PRJNA749892	GCA_021608475.1	A21-11	Contig	4.44	58.9	54	3956	2022-01-25T00:00:00Z
A. veronii	JAIEYM01	SAMN20667243	PRJNA749892	GCA_021608425.1	A21-13	Contig	4.37	58.9	55	3873	2022-01-25T00:00:00Z
A. veronii	JAIEYN01	SAMN20667244	PRJNA749892	GCA_021608405.1	A21-14	Contig	4.65	58.4	82	4099	2022-01-25T00:00:00Z
A. veronii	JAIEYO01	SAMN20667245	PRJNA749892	GCA_021608355.1	A21-15	Contig	4.51	58.8	68	3984	2022-01-25T00:00:00Z
A. veronii	JAIEYP01	SAMN20667246	PRJNA749892	GCA_021608365.1	A21-16	Contig	4.37	58.8	37	3891	2022-01-25T00:00:00Z
A. veronii	JAIEYQ01	SAMN20667247	PRJNA749892	GCA_021608345.1	A21-19	Contig	4.66	58.6	37	4186	2022-01-25T00:00:00Z
A. veronii	JAIEYR01	SAMN20667248	PRJNA749892	GCA_021608325.1	A21-4	Contig	4.61	58.7	76	4151	2022-01-25T00:00:00Z
A. veronii	JAIEYS01	SAMN20667249	PRJNA749892	GCA_021608285.1	A21-5	Contig	4.64	58.8	48	4178	2022-01-25T00:00:00Z
A. veronii	JAIEYT01	SAMN20667250	PRJNA749892	GCA_021608275.1	A21-6	Contig	4.49	58.5	43	3980	2022-01-25T00:00:00Z
A. veronii	JAIEYU01	SAMN20667251	PRJNA749892	GCA_021608235.1	A21-8	Contig	4.52	58.8	50	4050	2022-01-25T00:00:00Z
A. veronii	JAJDSR01	SAMN21168052	PRJNA759416	GCA_020639385.1	71474	Contig	4.64	58.6	6	4069	2021-10-26T00:00:00Z
A. veronii	JALJOG01	SAMN26746239	PRJNA817062	GCA_022953355.1	NBCF28	Contig	4.62	58.7	87	4195	2022-04-12T00:00:00Z
A. veronii	JALJOH01	SAMN26746686	PRJNA817067	GCA_022953315.1	NPSF15	Contig	4.50	58.7	56	4025	2022-04-12T00:00:00Z
A. veronii	JALJOI01	SAMN26746759	PRJNA817070	GCA_022953335.1	CBSB03	Contig	4.62	58.6	78	4147	2022-04-12T00:00:00Z
A. veronii	JRBE01	SAMN03018688	PRJNA260226	GCA_000754905.1	ARB3	Contig	4.54	58.8	63	4052	2014-09-16T00:00:00Z
A. veronii	LKJN01	SAMN04099649	PRJNA296464	GCA_001446465.1	TTU2014-108AME	Contig	4.53	58.7	62	4034	2015-11-19T00:00:00Z
A. veronii	LKJP01	SAMN04099650	PRJNA296464	GCA_001446505.1	TTU2014-108ASC	Contig	4.53	58.7	58	4034	2015-11-19T00:00:00Z
A. veronii	LKJS01	SAMN04099653	PRJNA296464	GCA_001446575.1	TTU2014-115ASC	Contig	4.53	58.7	52	4032	2015-11-19T00:00:00Z
A. veronii	LKJV01	SAMN04099656	PRJNA296464	GCA_001446635.1	TTU2014-125ASC	Contig	4.68	58.6	58	4164	2015-11-19T00:00:00Z
A. veronii	LKJW01	SAMN04099657	PRJNA296464	GCA_001446645.1	TTU2014-130AME	Contig	4.68	58.6	64	4164	2015-11-19T00:00:00Z
A. veronii	LKJY01	SAMN04099659	PRJNA296464	GCA_001446695.1	TTU2014-131ASC	Contig	4.68	58.6	70	4168	2015-11-19T00:00:00Z
A. veronii	LKKA01	SAMN04099661	PRJNA296464	GCA_001446735.1	TTU2014-134AME	Contig	4.68	58.6	50	4164	2015-11-19T00:00:00Z
A. veronii	LKKB01	SAMN04099662	PRJNA296464	GCA_001446755.1	TTU2014-134ASC	Contig	4.68	58.6	59	4169	2015-11-19T00:00:00Z
A. veronii	LKKC01	SAMN04099663	PRJNA296464	GCA_001446775.1	TTU2014-140ASC	Contig	4.68	58.6	81	4170	2015-11-19T00:00:00Z
A. veronii	LKKE01	SAMN04099665	PRJNA296464	GCA_001446815.1	TTU2014-141ASC	Contig	4.68	58.6	45	4164	2015-11-19T00:00:00Z
A. veronii	LKKF01	SAMN04099666	PRJNA296464	GCA_001446835.1	TTU2014-142ASC	Contig	4.68	58.6	45	4167	2015-11-19T00:00:00Z
A. veronii	LKKG01	SAMN04099667	PRJNA296464	GCA_001446855.1	TTU2014-143AME	Contig	4.68	58.6	59	4168	2015-11-19T00:00:00Z
A. veronii	LKKH01	SAMN04099668	PRJNA296464	GCA_001446875.1	TTU2014-143ASC	Contig	4.68	58.6	54	4165	2015-11-19T00:00:00Z
A. veronii	LRBO01	SAMN03455937	PRJNA279618	GCA_001647435.1	AVNIH2	Contig	4.52	58.9	50	3971	2016-04-28T00:00:00Z
A. veronii	LXJN01	SAMN04914378	PRJNA320014	GCA_001696435.1	VBF557	Contig	4.70	58.4	526	3325	2016-07-28T00:00:00Z
A. veronii	MRUI01	SAMN06130232	PRJNA356925	GCA_001921885.1	pamvotica	Contig	4.92	58.1	21	4366	2016-12-26T00:00:00Z
A. veronii	MRZQ01	SAMN05860749	PRJNA345309	GCA_001908555.1	CCM 7244	Contig	4.42	58.9	74	3875	2016-12-19T00:00:00Z
A. veronii	MRZR01	SAMN05860752	PRJNA345311	GCA_001908535.1	CCM 4359	Contig	4.51	58.9	56	3943	2016-12-19T00:00:00Z
A. veronii	MVPS01	SAMN06329944	PRJNA374665	GCA_013620785.1	161	Contig	4.51	58.7	28	3999	2020-07-24T00:00:00Z
A. veronii	MVPT01	SAMN06335474	PRJNA374781	GCA_013620815.1	CL8155	Contig	4.68	58.6	50	4148	2020-07-24T00:00:00Z
A. veronii	NKWH01	SAMN07312771	PRJNA391781	GCA_006243395.1	Hm22	Contig	4.93	58.3	61	4442	2019-06-11T00:00:00Z
A. veronii	NMUR01	SAMN07357117	PRJNA396650	GCA_003367145.1	NS	Contig	4.71	58.5	140	4250	2018-08-07T00:00:00Z
A. veronii	NMUS01	SAMN07357136	PRJNA396650	GCA_003367105.1	PDB	Contig	4.72	58.5	141	4286	2018-08-07T00:00:00Z
A. veronii	NNSE01	SAMN07375086	PRJNA396650	GCA_003367135.1	AG_5.28.6	Contig	4.61	58.6	98	4124	2018-08-07T00:00:00Z
A. veronii	NNSF01	SAMN07375275	PRJNA396650	GCA_003367095.1	VCK_1	Contig	4.63	58.6	120	4141	2018-08-07T00:00:00Z
A. veronii	NPKC01	SAMN07430239	PRJNA396650	GCA_004379225.1	NS_6.15.2	Contig	4.72	58.5	149	4267	2019-03-26T00:00:00Z
A. veronii	NPKD01	SAMN07430296	PRJNA396650	GCA_004379215.1	BIOO50A	Contig	4.61	58.6	109	4123	2019-03-26T00:00:00Z
A. veronii	NPKE01	SAMN07430307	PRJNA396650	GCA_004379205.1	NS2	Contig	4.72	58.5	143	4264	2019-03-26T00:00:00Z
A. veronii	NQMB01	SAMN07488420	PRJNA396650	GCA_008119755.1	NS13	Contig	4.67	58.6	139	4212	2019-08-27T00:00:00Z
A. veronii	NQMC01	SAMN07488421	PRJNA396650	GCA_008119745.1	NS22	Contig	4.74	58.4	172	4305	2019-08-27T00:00:00Z
A. veronii	PPUW01	SAMN08391006	PRJNA431414	GCA_002906945.1	ML09-123	Contig	4.75	58.4	32	4230	2018-01-30T00:00:00Z
A. veronii	PXYZ01	SAMN07972600	PRJNA416949	GCA_003013515.1	ZWY-AV1	Contig	4.62	58.6	31	4158	2018-03-21T00:00:00Z
A. veronii	PZKL01	SAMN08812721	PRJNA447880	GCA_003036425.1	XH.VA.1	Contig	5.36	56.5	62	4948	2018-04-05T00:00:00Z
A. veronii	RAWX01	SAMN10105084	PRJNA492757	GCA_003611985.1	MS 17-88	Contig	5.18	58.2	13	4677	2018-10-04T00:00:00Z
A. veronii	RHDQ01	SAMN10289379	PRJNA498295	GCA_003859745.1	312M	Contig	4.57	58.6	14	4041	2018-12-03T00:00:00Z
A. veronii	VATZ02	SAMN11620977	PRJNA542390	GCA_008362895.2	CTe-01	Contig	4.68	58.6	200	4114	2019-09-09T00:00:00Z
A. veronii	VWTS01	SAMN12706442	PRJNA564235	GCA_015208765.1	A136	Contig	4.67	58.4	41	4141	2020-11-02T00:00:00Z
A. veronii	VWTT01	SAMN12706443	PRJNA564235	GCA_015208715.1	A86	Contig	4.64	58.5	42	4112	2020-11-02T00:00:00Z
A. veronii	VWTV01	SAMN12706445	PRJNA564235	GCA_015208735.1	A34	Contig	4.64	58.5	82	4121	2020-11-02T00:00:00Z
A. veronii	VWTW01	SAMN12706446	PRJNA564235	GCA_015208655.1	A31	Contig	4.64	58.5	84	4124	2020-11-02T00:00:00Z
A. veronii	VWTX01	SAMN12706447	PRJNA564235	GCA_015208695.1	A5	Contig	4.77	58.2	33	4253	2020-11-02T00:00:00Z
A. veronii	WVRP01	SAMN13631568	PRJNA596393	GCA_009834905.1	CMF	Contig	4.56	58.7	200	4084	2020-01-08T00:00:00Z
A. allosaccharophila	CDBR01	SAMEA2752428	PRJEB7019	GCA_000819685.1	CECT 4199	Scaffold	4.66	58.4	120	4137	2014-11-04T00:00:00Z
A. allosaccharophila	CDCB01	SAMEA2752403	PRJEB7045	GCA_000820245.1	BVH88	Scaffold	4.71	58.6	131	4245	2014-11-24T00:00:00Z
A. allosaccharophila	JAKCNC01	SAMN24244993	PRJNA762299	GCA_021441165.1	INSAq178	Scaffold	4.52	58.7	309	4032	2022-01-11T00:00:00Z
A. allosaccharophila	NKWZ01	SAMN07312751	PRJNA391781	GCA_006246445.1	ATCC 35942	Scaffold	4.53	59.1	102	4063	2019-06-11T00:00:00Z
A. allosaccharophila	NXBS01	SAMN07680234	PRJNA408193	GCA_003265495.1	Z9-6	Scaffold	4.59	58.8	66	4042	2018-06-26T00:00:00Z
A. allosaccharophila	PQWC01	SAMN08436981	PRJNA432149	GCA_010906985.1	AE59-TE2	Scaffold	4.52	58.7	57	3997	2020-02-21T00:00:00Z
A. allosaccharophila	UETN01	SAMEA4743713	PRJEB27351	GCA_900491725.1	Z9-6	Scaffold	4.59	58.8	66	4057	2018-06-24T00:00:00Z
A. bestiarum	CDDA01	SAMEA2752425	PRJEB7022	GCA_000819745.1	CECT 4227	Scaffold	4.69	60.6	41	4136	2014-11-24T00:00:00Z
A. bivalvium	CDBT01	SAMEA2752424	PRJEB7023	GCA_000819765.1	CECT 7113	Scaffold	4.30	62.2	63	3836	2014-11-24T00:00:00Z
A. bivalvium	NXBQ01	SAMN07680236	PRJNA408193	GCA_003265465.1	ZJ19-2	Scaffold	4.26	62.4	38	3794	2018-06-26T00:00:00Z
A. bivalvium	NXBX01	SAMN07680229	PRJNA408193	GCA_003265565.1	ZJ20-2	Scaffold	4.30	62.2	84	3829	2018-06-26T00:00:00Z
A. bivalvium	UETJ01	SAMEA4743708	PRJEB27351	GCA_900491735.1	ZJ20-2	Scaffold	4.30	62.2	84	3828	2018-06-24T00:00:00Z
A. bivalvium	UETP01	SAMEA4743715	PRJEB27351	GCA_900491705.1	ZJ19-2	Scaffold	4.26	62.4	38	3793	2018-06-24T00:00:00Z
A. caviae	CDBK01	SAMEA2752423	PRJEB7024	GCA_000819785.1	CECT 838	Scaffold	4.47	61.7	111	4004	2014-11-24T00:00:00Z
A. caviae	CDBS01	SAMEA2752402	PRJEB7046	GCA_000820265.2	CECT 4221	Scaffold	4.58	61.1	332	4129	2014-11-24T00:00:00Z
A. caviae	JAAALU01	SAMN13763659	PRJNA600002	GCA_009906325.1	AK245	Scaffold	4.45	61.6	203	3975	2020-01-19T00:00:00Z
A. caviae	JAAALV01	SAMN13763658	PRJNA600002	GCA_009905865.1	BVH98	Scaffold	4.46	61.4	159	3963	2020-01-19T00:00:00Z

A. caviae	JAAALW01	SAMN13763657	PRJNA600002	GCA_009906375.1	BVH84	Scaffold	4.48	61.3	151	3988	2020-01-19T00:00:00Z
A. caviae	JAAALX01	SAMN13763656	PRJNA600002	GCA_009906335.1	ADV118	Scaffold	4.39	61.5	140	3921	2020-01-19T00:00:00Z
A. caviae	JAAROF01	SAMN14272287	PRJNA612546	GCA_023093415.1	6597	Scaffold	4.46	61.4	153	4023	2022-04-22T00:00:00Z
A. caviae	JAAVMO01	SAMN14470507	PRJNA615899	GCA_012102435.1	UFMG-H8	Scaffold	4.55	61.7	30	4084	2020-04-06T00:00:00Z
A. caviae	JAKCNV01	SAMN24245012	PRJNA762299	GCA_021440765.1	INSAq239	Scaffold	4.37	61.3	363	3992	2022-01-11T00:00:00Z
A. caviae	LESK01	SAMN03280160	PRJNA271899	GCA_001030105.1	BWH65	Scaffold	4.45	61.7	5	3972	2015-06-19T00:00:00Z
A. caviae	LFXO01	SAMN03778994	PRJNA287226	GCA_001183595.1	A23	Scaffold	4.52	61.3	143	4080	2015-07-21T00:00:00Z
A. caviae	MDSC01	SAMN05525171	PRJNA338160	GCA_001730205.1	CHZ306	Scaffold	4.79	60.7	65	4180	2016-09-16T00:00:00Z
A. caviae	MDSD01	SAMN05587054	PRJNA339286	GCA_001730215.1	CH129	Scaffold	4.42	61.6	26	3871	2016-09-16T00:00:00Z
A. caviae	NMSG01	SAMN07312780	PRJNA391781	GCA_006243135.1	TCO22	Scaffold	4.57	61.2	178	4173	2019-06-11T00:00:00Z
A. caviae	NXBP01	SAMN07680237	PRJNA408193	GCA_003265425.1	ZJ66-1	Scaffold	4.39	61.5	150	3944	2018-06-26T00:00:00Z
A. caviae	NXBR01	SAMN07680235	PRJNA408193	GCA_003265475.1	ZJ17-2	Scaffold	4.56	61.3	256	4121	2018-06-26T00:00:00Z
A. caviae	QKOU01	SAMN09289754	PRJNA472583	GCA_004024475.1	GEO_47_Up_A	Scaffold	4.51	61.6	261	4086	2019-01-15T00:00:00Z
A. caviae	QKOZ01	SAMN09289749	PRJNA472583	GCA_004024195.1	GEO_39_Eff_A	Scaffold	4.56	61.6	239	4160	2019-01-15T00:00:00Z
A. caviae	QKPA01	SAMN09289748	PRJNA472583	GCA_004024185.1	GEO_37_Down_A	Scaffold	4.72	61.1	467	4377	2019-01-15T00:00:00Z
A. caviae	QKPH01	SAMN09289741	PRJNA472583	GCA_004024325.1	GEO_23_Down_B	Scaffold	4.93	60.8	250	4563	2019-01-15T00:00:00Z
A. caviae	QLLA01	SAMN09428654	PRJNA476209	GCA_003350165.1	HAMBI_1972	Scaffold	4.46	61.8	51	3998	2018-07-31T00:00:00Z
A. caviae	RQJY01	SAMN10457758	PRJNA506284	GCA_003857325.1	Aer268	Scaffold	4.54	61.2	612	4192	2018-12-03T00:00:00Z
A. caviae	UETG01	SAMEA4743716	PRJEB27351	GCA_900491695.1	ZJ66-1	Scaffold	4.39	61.5	150	3945	2018-06-24T00:00:00Z
A. caviae	UETK01	SAMEA4743714	PRJEB27351	GCA_900491645.1	ZJ17-2	Scaffold	4.56	61.3	256	4121	2018-06-24T00:00:00Z
A. caviae	VZQB01	SAMN12777809	PRJNA566093	GCA_008802295.1	D	Scaffold	4.35	62.0	107	3906	2019-10-01T00:00:00Z
A. dhakensis	AGWR01	SAMN02463947	PRJNA71509	GCA_000298055.1	SSU	Scaffold	4.94	61.5	2	4431	2012-09-17T00:00:00Z
A. dhakensis	CDBH01	SAMEA2752400	PRJEB7048	GCA_000820305.1	CIP 107500	Scaffold	4.71	61.8	73	4229	2014-11-24T00:00:00Z
A. dhakensis	CDBP01	SAMEA2752427	PRJEB7020	GCA_000819705.1	CECT 7289	Scaffold	4.69	61.9	78	4232	2014-11-24T00:00:00Z
A. dhakensis	JAACNA01	SAMN13343730	PRJNA590952	GCA_019348695.1	26M	Scaffold	4.90	61.4	26	4354	2021-07-26T00:00:00Z
A. dhakensis	JAAKGG01	SAMN14124457	PRJNA607226	GCA_017163915.1	Aer_On15M	Scaffold	4.88	61.4	18	4343	2021-03-03T00:00:00Z
A. dhakensis	JAGFDX01	SAMN17860435	PRJNA701275	GCA_017582505.1	15996	Scaffold	4.79	61.8	63	4250	2021-03-24T00:00:00Z
A. dhakensis	JAGFEC01	SAMN17860440	PRJNA701275	GCA_017582425.1	16006	Scaffold	4.88	61.8	160	4348	2021-03-24T00:00:00Z
A. dhakensis	LBDI01	SAMN03491905	PRJNA281575	GCA_002734175.1	Cr1	Scaffold	4.98	61.4	79	4589	2017-10-27T00:00:00Z
A. dhakensis	LBDJ01	SAMN03491910	PRJNA281576	GCA_002734165.1	Cr2	Scaffold	4.98	61.4	75	4586	2017-10-27T00:00:00Z
A. dhakensis	LJOE01	SAMN04038585	PRJNA294992	GCA_001306015.1	KOR1	Scaffold	4.78	61.8	5	4186	2015-10-02T00:00:00Z
A. dhakensis	NKWO01	SAMN07312764	PRJNA391781	GCA_006243335.1	BVH70	Scaffold	4.72	61.7	59	4247	2019-06-11T00:00:00Z
A. dhakensis	NKWP01	SAMN07312763	PRJNA391781	GCA_006246345.1	BVH69	Scaffold	4.80	61.7	61	4354	2019-06-11T00:00:00Z
A. dhakensis	NKWQ01	SAMN07312762	PRJNA391781	GCA_006246145.1	BVH68	Scaffold	4.86	61.7	82	4418	2019-06-11T00:00:00Z
A. dhakensis	NKWT01	SAMN07312758	PRJNA391781	GCA_006246155.1	BVH43	Scaffold	4.98	61.4	57	4546	2019-06-11T00:00:00Z
A. dhakensis	PDXI01	SAMN07312761	PRJNA391781	GCA_006226215.1	BVH65	Scaffold	4.79	61.7	83	4321	2019-06-10T00:00:00Z
A. dhakensis	QUOJ01	SAMN09770288	PRJNA484846	GCA_003432465.1	17FW001	Scaffold	4.67	61.9	28	4149	2018-08-22T00:00:00Z
A. dhakensis	RJCW01	SAMN10390361	PRJNA504324	GCA_003723975.1	AE-13	Scaffold	4.71	61.8	36	4237	2018-11-13T00:00:00Z
A. diversa	CDCE01	SAMEA2752422	PRJEB7026	GCA_000819805.1	CECT 4254	Scaffold	4.06	61.6	37	3696	2014-11-24T00:00:00Z
A. encheleia	CDDI01	SAMEA2752421	PRJEB7027	GCA_000819825.1	CECT 4342	Scaffold	4.47	62.0	35	4024	2014-11-24T00:00:00Z
A. enteropelogenes	CDCG01	SAMEA2752420	PRJEB7028	GCA_000819845.1	CECT 4487	Scaffold	4.47	59.7	46	4037	2014-11-24T00:00:00Z
A. enteropelogenes	CDDE01	SAMEA2752405	PRJEB7043	GCA_000820205.1	CECT 4255T	Scaffold	4.34	60.1	27	3861	2014-11-24T00:00:00Z
A. enteropelogenes	RQKA01	SAMN10457756	PRJNA506284	GCA_003849645.1	Aer371	Scaffold	4.33	60.0	280	3753	2018-11-28T00:00:00Z
A. eucrenophila	CDDF01	SAMEA2752419	PRJEB7029	GCA_000819865.1	CECT 4224	Scaffold	4.54	61.2	22	4028	2014-12-01T00:00:00Z
A. fluvialis	CDBO01	SAMEA2752418	PRJEB7030	GCA_000819885.1	CDBO01	Scaffold	3.90	58.3	76	3473	2014-12-01T00:00:00Z
A. hydrophila subsp. *ranae*	CDDC01	SAMEA2752399	PRJEB7049	GCA_000820325.1	CIP 107985	Scaffold	4.68	61.6	107	4223	2014-11-24T00:00:00Z
A. hydrophila	DBMF01	SAMN06453168	PRJNA348753	GCA_002297625.1	UBA705	Scaffold	4.25	61.6	282	NA	2017-09-12T00:00:00Z
A. hydrophila	DYMQ01	SAMN22209478	PRJNA767587	GCA_020721505.1	SulCav_FS06-10_54_7	Scaffold	1.72	54.4	1056	NA	2021-11-02T00:00:00Z
A. hydrophila	FTME01	SAMN05878295	PRJEB18852	GCA_900155975.1	RU34A	Scaffold	4.83	61.2	25	4314	2017-01-14T00:00:00Z
A. hydrophila	FTNG01	SAMN05880569	PRJEB18864	GCA_900156325.1	RU34C	Scaffold	4.81	61.2	23	4304	2017-01-14T00:00:00Z
A. hydrophila	JAABKF01	SAMN13424805	PRJNA592442	GCA_019399965.1	Brac54	Scaffold	4.75	61.5	22	4217	2021-07-29T00:00:00Z
A. hydrophila	JAABKG01	SAMN13412801	PRJNA592155	GCA_019399995.1	Pi11	Scaffold	4.77	61.4	40	4218	2021-07-29T00:00:00Z
A. hydrophila	JAABKI01	SAMN13427745	PRJNA592528	GCA_019399935.1	Aer_Brac46	Scaffold	4.90	61.3	48	4318	2021-07-29T00:00:00Z
A. hydrophila	JAACMX01	SAMN13339007	PRJNA590794	GCA_019348635.1	GTCBM_22	Scaffold	5.17	61.0	48	4652	2021-07-26T00:00:00Z
A. hydrophila	JAADJL01	SAMN13382606	PRJNA591484	GCA_019348745.1	Aer_On19M	Scaffold	4.80	61.5	47	4203	2021-07-26T00:00:00Z
A. hydrophila	JAADJN01	SAMN13383158	PRJNA591552	GCA_019348795.1	Aer_On1M	Scaffold	4.96	61.0	132	4462	2021-07-26T00:00:00Z
A. hydrophila	JACSWK01	SAMN15915178	PRJNA659547	GCA_021735805.1	Ah_On10M	Scaffold	5.15	61.1	52	4614	2022-01-31T00:00:00Z
A. hydrophila	JACSWL01	SAMN15915372	PRJNA659551	GCA_021735825.1	Ah_On23M	Scaffold	5.28	61.1	49	4728	2022-01-31T00:00:00Z
A. hydrophila	JACSWM01	SAMN15915553	PRJNA659562	GCA_021735845.1	Ah_On16M	Scaffold	5.12	61.3	42	4596	2022-01-31T00:00:00Z
A. hydrophila	JAFLWS01	SAMN18099584	PRJNA705736	GCA_017315485.1	S73-1	Scaffold	4.72	61.4	27	4220	2021-03-10T00:00:00Z
A. hydrophila	JALKAG01	SAMN27512291	PRJNA823662	GCA_023016135.1	Ah2101	Scaffold	5.19	60.8	113	4682	2022-04-16T00:00:00Z
A. hydrophila	JVCD01	SAMN03197759	PRJNA267549	GCA_001057275.1	56_AHYD	Scaffold	4.68	61.6	99	4183	2015-07-10T00:00:00Z
A. hydrophila	JVDL01	SAMN03197725	PRJNA267549	GCA_001055245.1	53_AHYD	Scaffold	4.67	61.6	101	4179	2015-07-10T00:00:00Z
A. hydrophila	JVDW01	SAMN03197714	PRJNA267549	GCA_001057115.1	52_AHYD	Scaffold	4.68	61.6	111	4180	2015-07-10T00:00:00Z
A. hydrophila	JVES01	SAMN03197692	PRJNA267549	GCA_001055195.1	50_AHYD	Scaffold	4.67	61.6	108	4179	2015-07-10T00:00:00Z
A. hydrophila	JVFM01	SAMN03197672	PRJNA267549	GCA_001056955.1	48_AHYD	Scaffold	4.70	61.6	72	4191	2015-07-10T00:00:00Z
A. hydrophila	LSZC01	SAMN03742454	PRJNA297179	GCA_002834265.1	AH1	Scaffold	4.76	61.4	53	4282	2017-12-12T00:00:00Z
A. hydrophila	LZDC01	SAMN05212262	PRJNA324645	GCA_014332675.1	PW01	Scaffold	4.72	61.4	18	4170	2020-08-28T00:00:00Z
A. hydrophila	MJGE01	SAMN05791072	PRJNA343595	GCA_014839605.1	4LNC202	Scaffold	5.00	60.9	32	4454	2020-09-30T00:00:00Z
A. hydrophila	MJGY01	SAMN05792599	PRJNA343741	GCA_001939675.1	4LNG101	Scaffold	4.99	60.1	27	3978	2017-01-09T00:00:00Z
A. hydrophila	MRDF01	SAMN06112127	PRJNA356344	GCA_001937135.1	HZAUAH	Scaffold	5.04	60.9	32	4475	2017-01-05T00:00:00Z
A. hydrophila	MTGJ01	SAMN05721785	PRJNA341407	GCA_001999685.1	ATCC 7966	Scaffold	4.67	61.6	132	4167	2017-02-13T00:00:00Z
A. hydrophila	NADM01	SAMN06618438	PRJNA379721	GCA_002093135.1	Ah DBHS101	Scaffold	5.00	60.9	36	4444	2017-04-17T00:00:00Z
A. hydrophila	NBOV01	SAMN06674713	PRJNA381330	GCA_002114075.1	BSK-10	Scaffold	4.96	61.0	34	4406	2017-04-28T00:00:00Z
A. hydrophila	NBWY01	SAMN06675356	PRJNA381370	GCA_002115515.1	TPS-30	Scaffold	4.93	61.2	32	4373	2017-04-28T00:00:00Z
A. hydrophila	NKWA01	SAMN07312778	PRJNA391781	GCA_006243205.1	PAQ091014-21	Scaffold	4.78	61.3	68	4257	2019-06-11T00:00:00Z
A. hydrophila	NKWC01	SAMN07312776	PRJNA391781	GCA_006243265.1	PAQ091014-12	Scaffold	4.97	61.4	95	4541	2019-06-11T00:00:00Z
A. hydrophila	NKWD01	SAMN07312775	PRJNA391781	GCA_006243275.1	PAQ091014-9	Scaffold	4.93	60.9	72	4431	2019-06-11T00:00:00Z
A. hydrophila	NKWF01	SAMN07312773	PRJNA391781	GCA_006243365.1	PAQ091014-1	Scaffold	4.94	60.9	114	4452	2019-06-11T00:00:00Z
A. hydrophila	NKWV01	SAMN07312756	PRJNA391781	GCA_006246385.1	BAQ071013-136	Scaffold	4.97	61.0	84	4542	2019-06-11T00:00:00Z
A. hydrophila	NKXB01	SAMN07312749	PRJNA391781	GCA_006246455.1	ARS-131-14	Scaffold	4.93	60.9	70	4436	2019-06-11T00:00:00Z
A. hydrophila	NKXI01	SAMN07312742	PRJNA391781	GCA_006246545.1	2014-10509-28-27	Scaffold	4.96	61.1	107	4503	2019-06-11T00:00:00Z
A. hydrophila	QQBF01	SAMN09704980	PRJNA482427	GCA_014324385.1	W37	Scaffold	5.03	60.8	32	4421	2020-08-28T00:00:00Z
A. hydrophila	RQKC01	SAMN10457754	PRJNA506284	GCA_003849735.1	Aer284	Scaffold	4.82	61.2	198	4268	2018-11-28T00:00:00Z
A. hydrophila	WSYK01	SAMN13513309	PRJNA594349	GCA_016805345.1	Aer_WatGTCBM23	Scaffold	4.82	61.3	64	4334	2021-02-03T00:00:00Z
A. hydrophila subsp. *ranae*	WSYM01	SAMN13512719	PRJNA594328	GCA_016805405.1	Aer_On29M	Scaffold	4.77	61.5	48	4185	2021-02-03T00:00:00Z
A. hydrophila	WTZB01	SAMN13521181	PRJNA594573	GCA_019348835.1	Aer_OnAA	Scaffold	4.74	61.5	18	4200	2021-07-26T00:00:00Z
A. hydrophila	WTZC01	SAMN13525389	PRJNA594645	GCA_019348855.1	Aer_OnRU2	Scaffold	5.24	60.8	54	4757	2021-07-26T00:00:00Z
A. hydrophila	WTZD01	SAMN13526581	PRJNA594661	GCA_019348875.1	Aer_On3M	Scaffold	5.22	60.8	72	4730	2021-07-26T00:00:00Z
A. hydrophila	WTZE01	SAMN13522434	PRJNA594706	GCA_019348895.1	Aer_OnRU3	Scaffold	5.24	60.8	67	4751	2021-07-26T00:00:00Z
A. hydrophila	WTZF01	SAMN13499920	PRJNA594013	GCA_019348935.1	Aer_On22M	Scaffold	5.01	61.0	55	4468	2021-07-26T00:00:00Z

A. hydrophila	WTZI01	SAMN13541507	PRJNA594949	GCA_019348925.1	Aer_Brac14	Scaffold	4.97	61.2	38	4375	2021-07-26T00:00:00Z
A. hydrophila	WTZJ01	SAMN13541511	PRJNA594950	GCA_019348915.1	Aer_On27M	Scaffold	4.94	61.1	30	4394	2021-07-26T00:00:00Z
A. jandaei	CDBV01	SAMEA2752417	PRJEB7031	GCA_000819955.1	CECT 4228	Scaffold	4.50	59.0	58	4096	2014-11-24T00:00:00Z
A. jandaei	JAACNF01	SAMN13344030	PRJNA590971	GCA_019348715.1	Pi 2.2 HP AS	Scaffold	4.57	58.8	39	4099	2021-07-26T00:00:00Z
A. jandaei	JAAKGH01	SAMN14124565	PRJNA607235	GCA_016805365.1	Aer_On5M	Scaffold	4.51	59.0	34	4018	2021-02-03T00:00:00Z
A. jandaei	JACTGU01	SAMN15915549	PRJNA659560	GCA_021735865.1	Aj_Pi13.2HPAS	Scaffold	4.89	58.7	72	4400	2022-01-31T00:00:00Z
A. jandaei	NKWG01	SAMN07312772	PRJNA391781	GCA_006243385.1	Ho603	Scaffold	4.63	58.5	186	4174	2019-06-11T00:00:00Z
A. jandaei	PRCV01	SAMN08457750	PRJNA432735	GCA_002925765.1	IMET J	Scaffold	4.73	58.4	20	4273	2018-02-12T00:00:00Z
A. jandaei	WOXB01	SAMN13428651	PRJNA592537	GCA_016805325.1	Aer_On4M	Scaffold	4.53	59.0	55	4035	2021-02-03T00:00:00Z
A. media	CDBZ01	SAMEA2752416	PRJEB7032	GCA_000819985.1	CECT 4232	Scaffold	4.48	61.1	233	4043	2014-11-24T00:00:00Z
A. media	JAKCNH01	SAMN24244998	PRJNA762299	GCA_021441425.1	INSAq193	Scaffold	5.18	60.0	532	4704	2022-01-11T00:00:00Z
A. media	NKWW01	SAMN07312754	PRJNA391781	GCA_006246225.1	BAQ071013-132	Scaffold	4.69	61.3	165	4174	2019-06-11T00:00:00Z
A. media	NKWY01	SAMN07312752	PRJNA391781	GCA_006246425.1	BAQ071013-115	Scaffold	4.56	62.3	89	4080	2019-06-11T00:00:00Z
A. media	NXBV01	SAMN07680231	PRJNA408193	GCA_003265595.1	Z1-6	Scaffold	4.52	61.4	131	4001	2018-06-26T00:00:00Z
A. media	UETL01	SAMEA4743710	PRJEB27351	GCA_900491715.1	Z1-6	Scaffold	4.52	61.4	131	4008	2018-06-24T00:00:00Z
A. piscicola	CDBL01	SAMEA2752415	PRJEB7033	GCA_000820005.1	LMG 24783	Scaffold	5.18	59.2	91	4729	2014-11-24T00:00:00Z
A. popoffii	CDBI01	SAMEA2752414	PRJEB7034	GCA_000820025.1	CIP 105493	Scaffold	4.76	58.6	105	4240	2014-11-24T00:00:00Z
A. rivipollensis	JAAILB01	SAMN14091305	PRJNA606446	GCA_010974915.1	G42	Scaffold	4.58	61.3	55	4099	2020-02-23T00:00:00Z
A. rivipollensis	JAKCNB01	SAMN24244992	PRJNA762299	GCA_021441245.1	INSAq177	Scaffold	4.58	61.4	249	4134	2022-01-11T00:00:00Z
A. rivipollensis	JAKCND01	SAMN24244994	PRJNA762299	GCA_021441265.1	INSAq180	Scaffold	4.65	61.6	228	4187	2022-01-11T00:00:00Z
A. rivuli	CDBJ01	SAMEA2752413	PRJEB7035	GCA_000820045.1	DSM 22539	Scaffold	4.53	60.0	102	4139	2014-11-24T00:00:00Z
A. salmonicida subsp. *salmoni*	CDDW01	SAMEA2752412	PRJEB7036	GCA_000820065.1	CIP 103209	Scaffold	4.74	58.5	128	4260	2014-11-24T00:00:00Z
A. salmonicida	DMXS01	SAMN08018182	PRJNA417962	GCA_003483825.1	UBA10746	Scaffold	3.69	59.1	328	3502	2018-09-06T00:00:00Z
A. salmonicida	DNXW01	SAMN08018876	PRJNA417962	GCA_003505635.1	UBA8809	Scaffold	4.65	59.0	182	4324	2018-09-07T00:00:00Z
A. salmonicida	JAKCMZ01	SAMN24244988	PRJNA762299	GCA_021440455.1	INSAq169	Scaffold	4.17	58.5	386	3872	2022-01-11T00:00:00Z
A. salmonicida	JAKCNI01	SAMN24244999	PRJNA762299	GCA_021440865.1	INSAq195	Scaffold	5.25	58.4	264	4800	2022-01-11T00:00:00Z
A. salmonicida subsp. *salmoni*	JXTA01	SAMN03295755	PRJNA264317	GCA_000931985.2	JF3224	Scaffold	4.81	58.4	133	4328	2015-02-24T00:00:00Z
A. salmonicida subsp. *salmoni*	JYFF01	SAMN03332961	PRJNA264317	GCA_001499805.2	RS534	Scaffold	4.89	58.2	123	4397	2015-12-15T00:00:00Z
A. salmonicida subsp. *salmoni*	JYFG01	SAMN03332968	PRJNA264317	GCA_001597895.1	JF3791	Scaffold	4.73	58.5	111	4237	2016-03-25T00:00:00Z
A. salmonicida	JZTF01	SAMN03395034	PRJNA264317	GCA_001481535.2	Y47	Scaffold	4.71	58.5	118	4228	2015-12-15T00:00:00Z
A. salmonicida	JZTH01	SAMN03395037	PRJNA264317	GCA_001481545.2	Y577	Scaffold	4.74	58.6	104	4225	2015-12-15T00:00:00Z
A. salmonicida	LUHO01	SAMN04529529	PRJNA314146	GCA_002093695.1	BG	Scaffold	4.79	58.6	236	4325	2017-04-14T00:00:00Z
A. salmonicida	LUHP01	SAMN04529550	PRJNA314146	GCA_002093675.1	YK	Scaffold	4.70	58.6	204	4179	2017-04-14T00:00:00Z
A. salmonicida subsp. *pectinol*	NKWI01	SAMN07312770	PRJNA391781	GCA_006243325.1	CIP 107036	Scaffold	4.80	58.5	225	4276	2019-06-11T00:00:00Z
A. salmonicida subsp. *smithia*	NKWJ01	SAMN07312769	PRJNA391781	GCA_006246305.1	CIP 104757	Scaffold	4.53	58.8	234	3957	2019-06-11T00:00:00Z
A. salmonicida subsp. *achrom*	NKWK01	SAMN07312768	PRJNA391781	GCA_006246315.1	CIP 104001	Scaffold	4.62	59.0	540	4214	2019-06-11T00:00:00Z
A. salmonicida	NKWL01	SAMN07312767	PRJNA391781	GCA_006246135.1	CIP 103210	Scaffold	4.54	58.7	238	3944	2019-06-11T00:00:00Z
A. salmonicida	NXBT01	SAMN07680233	PRJNA408193	GCA_003265515.1	Z5-5	Scaffold	4.71	58.6	110	4158	2018-06-26T00:00:00Z
A. salmonicida	PSZK01	SAMN08524351	PRJNA264317	GCA_003947395.1	947C	Scaffold	4.78	58.4	48	4317	2018-12-14T00:00:00Z
A. salmonicida	QLLM01	SAMN09062889	PRJNA456363	GCA_003259515.1	17	Scaffold	4.90	58.5	51	4365	2018-06-21T00:00:00Z
A. salmonicida	UETH01	SAMEA4743712	PRJEB27351	GCA_900491655.1	Z5-5	Scaffold	4.71	58.6	110	4195	2018-06-24T00:00:00Z
A. sanarellii	CDBN01	SAMEA2752411	PRJEB7037	GCA_000820085.1	LMG 24682	Scaffold	4.19	63.1	98	3760	2014-11-24T00:00:00Z
A. sanarellii	JAGIQF01	SAMN18477559	PRJNA717099	GCA_017815105.1	MaGu-431	Scaffold	4.28	63.1	62	3807	2021-04-06T00:00:00Z
A. schubertii	CDDB01	SAMEA2752410	PRJEB7038	GCA_000820105.2	CECT 4240	Scaffold	4.13	61.7	111	3805	2014-11-24T00:00:00Z
A. schubertii	LPUO01	SAMN04299626	PRJNA304368	GCA_001481395.1	ATCC 43700	Scaffold	4.14	61.7	57	3791	2015-12-22T00:00:00Z
A. simiae	CDBY01	SAMEA2752409	PRJEB7039	GCA_000820125.1	CIP 107798	Scaffold	3.99	61.3	100	3657	2014-11-24T00:00:00Z
A. sobria	CDBW01	SAMEA2752408	PRJEB7040	GCA_000820145.1	CECT 4245	Scaffold	4.68	57.6	48	4165	2014-11-24T00:00:00Z
A. sobria	MKFU01	SAMN05828312	PRJNA344548	GCA_001858225.1	8005	Scaffold	4.69	57.6	52	4147	2016-11-01T00:00:00Z
A. sobria	NKWE01	SAMN07312774	PRJNA391781	GCA_006243315.1	PAQ091014-5	Scaffold	4.66	57.7	101	4168	2019-06-11T00:00:00Z
A. sobria	NKXA01	SAMN07312750	PRJNA391781	GCA_006246215.1	ARS-145-14	Scaffold	4.79	57.6	100	4304	2019-06-11T00:00:00Z
A. sobria	NKXJ01	SAMN07312741	PRJNA391781	GCA_006246595.1	2014-10509-27-20	Scaffold	4.64	57.7	200	4186	2019-06-11T00:00:00Z
A. taiwanensis	CDDD01	SAMEA2752407	PRJEB7041	GCA_000820165.1	LMG 24683	Scaffold	4.25	62.8	106	3806	2014-11-24T00:00:00Z
A. tecta	CDCA01	SAMEA2752406	PRJEB7042	GCA_000820185.2	CDCA01	Scaffold	4.75522	60.1	51	4257	2015-01-30T00:00:00Z
A. veronii	AGWT01	SAMN02463948	PRJNA71513	GCA_000297975.1	AER39	Scaffold	4.42	58.8	4	3889	2012-09-17T00:00:00Z
A. veronii	AGWU01	SAMN02463949	PRJNA71515	GCA_000298015.1	AMC34	Scaffold	4.58	58.4	1	4084	2012-09-17T00:00:00Z
A. veronii	AGWV01	SAMN02463950	PRJNA71517	GCA_000297995.1	AER397	Scaffold	4.50	58.8	5	3949	2012-09-17T00:00:00Z
A. veronii	AGWW01	SAMN02463951	PRJNA71519	GCA_000298035.1	AMC35	Scaffold	4.57	58.5	2	3980	2012-09-17T00:00:00Z
A. veronii	CABMOE01	SAMEA5852034	PRJEB33885	GCA_902388095.1	MGYG-HGUT-02529	Scaffold	4.70	58.4	124	4164	2019-08-12T00:00:00Z
A. veronii	CDBQ01	SAMEA2752397	PRJEB7051	GCA_000820385.1	LMG 13067	Scaffold	4.74	58.4	72	4228	2014-11-24T00:00:00Z
A. veronii	CDBU01	SAMEA2752398	PRJEB7050	GCA_000820365.1	CECT 4486	Scaffold	4.41	58.9	66	3943	2014-11-24T00:00:00Z
A. veronii	CDDK01	SAMEA2752404	PRJEB7044	GCA_000820225.1	CECT 4257	Scaffold	4.52	58.9	52	4054	2014-11-24T00:00:00Z
A. veronii	DDJB01	SAMN06453457	PRJNA348753	GCA_002339005.1	UBA1835	Scaffold	4.11	59.0	323	NA	2017-09-22T00:00:00Z
A. veronii	FTMU01	SAMN05892873	PRJEB18858	GCA_900156085.1	RU31B	Scaffold	4.53	58.7	93	4046	2017-01-14T00:00:00Z
A. veronii	JAACNB01	SAMN13343884	PRJNA590960	GCA_019348625.1	Pi6.2MC	Scaffold	4.74	58.5	51	4221	2021-07-26T00:00:00Z
A. veronii	JAACNE01	SAMN13343850	PRJNA590958	GCA_019348775.1	GT1	Scaffold	4.63	58.7	30	4085	2021-07-26T00:00:00Z
A. veronii	JACGXR01	SAMN15693637	PRJNA650139	GCA_015318285.1	XhG1.2	Scaffold	4.57	58.7	34	4041	2020-11-09T00:00:00Z
A. veronii	JACTGT01	SAMN15915530	PRJNA659557	GCA_021735905.1	Av_Pi4.2HPAS	Scaffold	5.02	58.9	55	4448	2022-01-31T00:00:00Z
A. veronii	JAEMTZ01	SAMN17138615	PRJNA512885	GCA_016461675.1	22	Scaffold	5.09	58.3	185	4616	2021-01-03T00:00:00Z
A. veronii	JAEMUA01	SAMN17138649	PRJNA512885	GCA_016461755.1	28	Scaffold	4.97	58.5	108	4497	2021-01-03T00:00:00Z
A. veronii	JAFLWQ01	SAMN18099582	PRJNA705736	GCA_017315455.1	S50-1	Scaffold	4.56	58.5	130	4066	2021-03-10T00:00:00Z
A. veronii	JAJOPS01	SAMN23553021	PRJNA785277	GCA_021165625.1	Z12	Scaffold	4.94	58.2	100	4457	2021-12-11T00:00:00Z
A. veronii	LKJQ01	SAMN04099651	PRJNA296464	GCA_001446535.1	TTU2014-113AME	Scaffold	4.66	58.6	122	4167	2015-11-19T00:00:00Z
A. veronii	LKJR01	SAMN04099652	PRJNA296464	GCA_001446555.1	TTU2014-115AME	Scaffold	4.53	58.7	53	4032	2015-11-19T00:00:00Z
A. veronii	LKJX01	SAMN04099658	PRJNA296464	GCA_001446675.1	TTU2014-130ASC	Scaffold	4.68	58.6	49	4165	2015-11-19T00:00:00Z
A. veronii	LKKD01	SAMN04099664	PRJNA296464	GCA_001446795.1	TTU2014-141AME	Scaffold	4.68	58.6	48	4164	2015-11-19T00:00:00Z
A. veronii	NJGB01	SAMN07252140	PRJNA329585	GCA_002214865.1	A29	Scaffold	4.48	58.8	54	3999	2017-07-05T00:00:00Z
A. veronii	NKVZ01	SAMN07312779	PRJNA391781	GCA_006243165.1	TCO21	Scaffold	4.46	58.7	93	3983	2019-06-11T00:00:00Z
A. veronii	NKWM01	SAMN07312766	PRJNA391781	GCA_006243415.1	CECT 7059	Scaffold	4.81	58.4	31	4289	2019-06-11T00:00:00Z
A. veronii	NKWN01	SAMN07312765	PRJNA391781	GCA_006246325.1	CECT 4902	Scaffold	4.64	58.4	29	4112	2019-06-11T00:00:00Z
A. veronii	NKWR01	SAMN07312760	PRJNA391781	GCA_006246355.1	BVH47	Scaffold	4.64	58.9	108	4232	2019-06-11T00:00:00Z
A. veronii	NKWS01	SAMN07312759	PRJNA391781	GCA_006246165.1	BVH46	Scaffold	4.51	58.8	39	4033	2019-06-11T00:00:00Z
A. veronii	NKWU01	SAMN07312757	PRJNA391781	GCA_006246175.1	BVH37	Scaffold	4.46	58.8	55	3986	2019-06-11T00:00:00Z
A. veronii	NKWX01	SAMN07312753	PRJNA391781	GCA_006246395.1	BAQ071013-116	Scaffold	4.58	58.6	82	4093	2019-06-11T00:00:00Z
A. veronii	NKXC01	SAMN07312748	PRJNA391781	GCA_006246485.1	AMC 25	Scaffold	4.60	58.8	50	4152	2019-06-11T00:00:00Z
A. veronii	NKXD01	SAMN07312747	PRJNA391781	GCA_006246495.1	AK247	Scaffold	4.55	58.8	36	4077	2019-06-11T00:00:00Z
A. veronii	NKXE01	SAMN07312746	PRJNA391781	GCA_006246235.1	AK241	Scaffold	4.60	58.6	42	4115	2019-06-11T00:00:00Z
A. veronii	NKXF01	SAMN07312745	PRJNA391781	GCA_006246515.1	AK236	Scaffold	4.41	58.8	26	3919	2019-06-11T00:00:00Z
A. veronii	NKXG01	SAMN07312744	PRJNA391781	GCA_006246555.1	AK227	Scaffold	4.40	58.7	67	3953	2019-06-11T00:00:00Z
A. veronii	NKXH01	SAMN07312743	PRJNA391781	GCA_006246275.1	ADV102	Scaffold	4.52	58.6	87	4086	2019-06-11T00:00:00Z
A. veronii	NXBU01	SAMN07680232	PRJNA408193	GCA_003265545.1	Z2-7	Scaffold	4.41	58.7	48	3926	2018-06-26T00:00:00Z
A. veronii	NXBY01	SAMN07680228	PRJNA408193	GCA_003265585.1	ZJ12-3	Scaffold	4.70	58.4	124	4164	2018-06-26T00:00:00Z

A. veronii	PDXJ01	SAMN07312755	PRJNA391781	GCA_006226255.1	BAQ071013-135	Scaffold	4.62	58.9	50	4197	2019-06-10T00:00:00Z
A. veronii	PPTE01	SAMN08383672	PRJNA431081	GCA_002900255.1	126-14	Scaffold	4.37	58.6	146	3879	2018-01-26T00:00:00Z
A. veronii	QQOQ01	SAMN09711306	PRJNA482711	GCA_003345755.1	XH.VA.2	Scaffold	4.91	58.1	48	4410	2018-07-30T00:00:00Z
A. veronii	RSFC01	SAMN10523237	PRJNA508461	GCA_004123695.1	A134	Scaffold	4.41	58.7	151	3952	2019-01-31T00:00:00Z
A. veronii	RZII01	SAMN10644079	PRJNA511801	GCA_003990305.1	CQ-AV1	Scaffold	4.78	58.5	36	4328	2019-01-01T00:00:00Z
A. veronii	SSUX01	SAMN11445140	PRJNA396650	GCA_004803495.1	XU1	Scaffold	4.80	58.0	92	4340	2019-04-22T00:00:00Z
A. veronii	SZVM01	SAMN11533742	PRJNA540223	GCA_007654995.1	WB12	Scaffold	4.52	58.8	40	3974	2019-07-30T00:00:00Z
A. veronii	UETI01	SAMEA4743711	PRJEB27351	GCA_900491685.1	Z2-7	Scaffold	4.41	58.7	48	3926	2018-06-24T00:00:00Z
A. veronii	UETM01	SAMEA4743707	PRJEB27351	GCA_900491675.1	ZJ12-3	Scaffold	4.70	58.4	124	4164	2018-06-24T00:00:00Z
A. veronii	VZQA01	SAMN12777764	PRJNA566091	GCA_008802305.1	D	Scaffold	4.43	59.0	149	3962	2019-10-01T00:00:00Z
A. veronii	WSFR01	SAMN13514135	PRJNA594379	GCA_009765635.1	B44	Scaffold	4.61	58.6	51	4101	2019-12-18T00:00:00Z
A. veronii	WSFS01	SAMN13518075	PRJNA594423	GCA_009765645.1	B48	Scaffold	4.73	58.7	49	4261	2019-12-18T00:00:00Z
A. veronii	WSYL01	SAMN13513355	PRJNA594352	GCA_016805375.1	Aer_WatCTCBM21	Scaffold	4.60	58.7	45	4083	2021-02-03T00:00:00Z

Supplementary Table 2. List of 300 *Aeromonas* query genomes used in the study

Serial n	Organism Name (GenBank)	Accession.no	BioSample	BioProject	Assembly	Strain	Level	Size(Mb)	GC%	Scaffolds	CDS	Release_Date
1	*A. australiensis*	OU015320.1	SAMEA8533644	PRJEB44171	GCA_907163065.1	Isolate4	Complete	4.56	61.1	1	NA	2021-05-12T00:00:00Z
2	*A. caviae*	AP019195.1	SAMD00144878	PRJDB6962	GCA_003925855.2	GSH8M-1	Complete	4.83	60.7	4	4249	2018-11-03T00:00:00Z
3	*A. caviae*	AP022214.1	SAMD00194595	PRJDB6962	GCA_014162015.1	WP8-S17-ESB	Complete	4.35	61.7	1	3815	2020-07-21T23:47:00Z
4	*A. caviae*	AP022241.1	SAMD00194620	PRJDB6962	GCA_014169675.1	WP8-S18-CRE	Complete	4.83	60.7	4	4248	2020-07-21T23:51:00Z
5	*A. caviae*	CP062787.1	SAMN02934523	PRJNA231221	GCA_000783775.2	FDAARGOS_7	Complete	4.53	61.7	1	3741	2020-10-13T00:00:00Z
6	*A. caviae*	CP024198.1	SAMN03389335	PRJNA277314	GCA_000959705.2	8LM	Complete	4.55	61.7	1	3431	2017-10-24T00:00:00Z
7	*A. caviae*	CP039832.1	SAMN11521871	PRJNA540105	GCA_007179295.1	WCW1-2	Complete	4.68	61.3	1	4084	2019-07-19T00:00:00Z
8	*A. caviae*	CP047982.1	SAMN13875147	PRJNA601850	GCA_013487985.1	1507-17068	Complete	4.53	61.3	1	3972	2020-07-23T00:00:00Z
9	*A. caviae*	CP047983.1	SAMN13875308	PRJNA601853	GCA_013488005.1	1605-27183	Complete	4.86	61.0	1	4255	2020-07-23T00:00:00Z
10	*A. caviae*	CP065937.1	SAMN17014572	PRJNA512885	GCA_016126815.2	Aero21	Complete	5.37	60.5	2	4604	2020-12-20T00:00:00Z
11	*A. caviae*	CP066813.1	SAMN17150322	PRJNA512885	GCA_016598815.1	Aero52	Complete	4.53	61.4	4	4016	2021-01-10T00:00:00Z
12	*A. caviae*	CP084350.1	SAMN21168050	PRJNA759416	GCA_020405325.1	71442	Complete	4.44	61.7	1	3909	2021-10-10T00:00:00Z
13	*A. caviae*	CP085468.1	SAMN21168053	PRJNA759416	GCA_020640975.1	71485	Complete	4.61	61.2	2	4037	2021-10-27T00:00:00Z
14	*A. dhakensis*	CP045311.1	SAMN13030278	PRJNA577584	GCA_022703095.1	Aer_OnIF1	Complete	4.83	61.6	1	4260	2022-03-28T00:00:00Z
15	*A. dhakensis*	CP084351.1	SAMN21168049	PRJNA759416	GCA_020405345.1	71431	Complete	4.77	61.7	1	4190	2021-10-10T00:00:00Z
16	*A. enteropelogenes*	CP083626.1	SAMN21218865	PRJNA231221	GCA_020097315.1	FDAARGOS_1	Complete	4.54	59.6	1	4024	2021-09-23T00:00:00Z
17	*A. hydrophila*	AP024234.1	SAMD00261012	PRJDB10897	GCA_016592295.1	RIMD111065	Complete	4.96	61.0	1	4331	2020-12-18T00:00:00Z
18	*A. hydrophila*	CP005966.1	SAMN02603614	PRJNA188141	GCA_000401555.1	ML09-119	Complete	5.02	60.8	1	4427	2013-05-24T00:00:00Z
19	*A. hydrophila*	CP006579.1	SAMN02641590	PRJNA210524	GCA_000512185.1	4AK4	Complete	4.53	62.0	1	3991	2013-12-30T00:00:00Z
20	*A. hydrophila*	CP016380.1	SAMN03294822	PRJNA273636	GCA_001687125.1	AHNIH1	Complete	5.05	61.1	2	4488	2016-07-18T00:00:00Z
21	*A. hydrophila*	CP010947.1	SAMN03366652	PRJNA270887	GCA_000940915.1	AL06-06	Complete	4.90	61.4	4	4382	2015-03-04T00:00:00Z
22	*A. hydrophila*	CP011100.1	SAMN03421355	PRJNA278509	GCA_000963645.1	AH10	Complete	4.91	61.1	1	4319	2015-03-26T00:00:00Z
23	*A. hydrophila*	CP013178.1	SAMN04266592	PRJNA302121	GCA_001455365.1	JBN2301	Complete	5.15	60.8	4	4565	2015-12-02T00:00:00Z
24	*A. hydrophila*	CP013965.1	SAMN04404355	PRJNA308632	GCA_001518775.1	D4	Complete	5.28	60.5	5	4690	2016-01-20T00:00:00Z
25	*A. hydrophila*	CP016990.1	SAMN05589911	PRJNA339336	GCA_003491245.1	ZYAH75	Complete	4.96	61.3	1	4361	2018-09-07T00:00:00Z
26	*A. hydrophila*	CP016989.1	SAMN05590109	PRJNA339368	GCA_003491225.1	ZYAH72	Complete	5.16	60.7	1	4536	2018-09-07T00:00:00Z
27	*A. hydrophila*	CP038463.1	SAMN11293745	PRJNA530076	GCA_004684305.1	WCX23	Complete	5.31	60.4	2	4710	2019-04-07T00:00:00Z
28	*A. hydrophila*	CP043323.1	SAMN12097415	PRJNA528964	GCA_014217525.1	3206	Complete	4.79	61.6	1	2738	2020-08-17T00:00:00Z
29	*A. hydrophila*	CP045501.1	SAMN13041437	PRJNA577921	GCA_022700835.1	Aer_Pi25.1HT/	Complete	4.81	61.2	1	4261	2022-03-28T00:00:00Z
30	*A. hydrophila*	CP050850.1	SAMN13353934	PRJNA591199	GCA_017310195.1	Brac6	Complete	4.77	61.5	1	4195	2021-03-09T00:00:00Z
31	*A. hydrophila*	CP050851.1	SAMN13545457	PRJNA595062	GCA_017310215.1	OnP3.1	Complete	4.77	61.5	1	4194	2021-03-09T00:00:00Z
32	*A. hydrophila*	CP046954.1	SAMN13567714	PRJNA595800	GCA_009791455.1	HX-3	Complete	4.94	61.0	1	4313	2019-12-23T00:00:00Z
33	*A. hydrophila*	CP053883.1	SAMN13926351	PRJNA528964	GCA_018802305.1	4960	Complete	4.83	61.5	1	4079	2021-06-09T00:00:00Z
34	*A. hydrophila*	CP064382.1	SAMN14078805	PRJNA605254	GCA_022869925.1	PartN-Ahydrop	Complete	4.73	61.6	1	4191	2022-04-11T00:00:00Z
35	*A. hydrophila*	CP050994.1	SAMN14533640	PRJNA622823	GCA_012273595.1	NEB724	Complete	4.57	61.7	1	3946	2020-04-12T00:00:00Z
36	*A. hydrophila*	CP084353.1	SAMN21168046	PRJNA759416	GCA_020405385.1	71317	Complete	4.70	61.7	1	4099	2021-10-10T00:00:00Z
37	*A. hydrophila*	CP093309.1	SAMN26377313	PRJNA812366	GCA_022631195.1	AC133	Complete	5.04	60.9	1	4400	2022-03-20T00:00:00Z
38	*A. hydrophila*	CP094267.1	SAMN26806291	PRJNA817329	GCA_022759545.1	A008N2	Complete	4.78	61.6	1	4205	2022-03-30T00:00:00Z
39	*A. hydrophila*	CP095280.1	SAMN27350375	PRJNA823662	GCA_022982835.1	Ah2111	Complete	5.32	60.7	8	4724	2022-04-14T00:00:00Z
40	*A. jandaei*	AP024466.1	SAMD00276024	PRJDB11115	GCA_016865345.1	JUNP479	Complete	4.54	59.0	2	4034	2021-02-02T00:00:00Z
41	*A. jandaei*	CP043321.1	SAMN12097417	PRJNA528964	GCA_014217485.1	3348	Complete	4.57	58.9	1	2499	2020-08-17T00:00:00Z
42	*A. jandaei*	CP053882.1	SAMN13926352	PRJNA528964	GCA_018802365.1	3036	Complete	4.59	59.0	1	3936	2021-06-09T00:00:00Z
43	*A. jandaei*	CP053881.1	SAMN13926353	PRJNA528964	GCA_018802325.1	4608	Complete	4.51	59.0	1	4004	2021-06-09T00:00:00Z
44	*A. jandaei*	CP053879.1	SAMN13926354	PRJNA528964	GCA_018802265.1	4956	Complete	4.65	58.5	2	3742	2021-06-09T00:00:00Z
45	*A. media*	AP022188.1	SAMD00194563	PRJDB6962	GCA_014161425.1	WP7-W18-ESI	Complete	4.76	61.0	1	4165	2020-07-21T23:42:00Z
46	*A. media*	CP007567.1	SAMN02472129	PRJNA170164	GCA_000287215.3	WS	Complete	4.79	60.7	2	4089	2014-04-22T00:00:00Z
47	*A. media*	CP038441.1	SAMN11269565	PRJNA529511	GCA_013085485.1	T0.1-19	Complete	4.92	61.0	2	4269	2020-05-14T00:00:00Z
48	*A. media*	CP038444.1	SAMN11269567	PRJNA529511	GCA_013085745.1	T5-8	Complete	4.79	60.8	1	4133	2020-05-14T00:00:00Z
49	*A. media*	CP038448.1	SAMN11269569	PRJNA529511	GCA_013085785.1	R50-22	Complete	5.15	60.2	3	4430	2020-05-14T00:00:00Z
50	*A. media*	CP043579.1	SAMN12671375	PRJNA563308	GCA_014109865.1	R1-26	Complete	4.67	60.8	1	4069	2020-08-07T00:00:00Z
51	*A. media*	CP061477.1	SAMN15963531	PRJNA660910	GCA_019455365.1	T5-1	Complete	5.04	60.9	2	3856	2021-08-05T00:00:00Z
52	*A. media*	CP075564.1	SAMN19237318	PRJNA730636	GCA_020423125.1	TR3_1	Complete	4.53	61.2	2	3954	2021-10-12T00:00:00Z
53	*A. salmonicida* subsp. *masouci*	CP017143.1	SAMN05735119	PRJNA342065	GCA_002313065.1	RFAS1	Complete	4.92	58.3	3	4220	2017-09-19T00:00:00Z
54	*A. salmonicida*	CP021654.1	SAMN06812489	PRJNA383853	GCA_002180335.1	O23A	Complete	4.94	58.1	5	4379	2017-06-08T00:00:00Z
55	*A. salmonicida*	CP022186.1	SAMN07276873	PRJNA391844	GCA_002214265.1	S68	Complete	4.88	58.4	5	4216	2017-07-03T00:00:00Z
56	*A. salmonicida*	CP022181.1	SAMN07276874	PRJNA391845	GCA_002214305.1	S44	Complete	5.08	58.2	6	4396	2017-07-03T00:00:00Z
57	*A. salmonicida* subsp. *salmonic*	CP047374.1	SAMN13518349	PRJNA594426	GCA_009858115.1	J409	Complete	4.73	58.6	2	4031	2020-01-13T00:00:00Z
58	*A. salmonicida* subsp. *salmonic*	CP052034.1	SAMN13518351	PRJNA594426	GCA_012933685.1	J411	Complete	4.72	58.6	2	3456	2020-04-30T00:00:00Z
59	*A. salmonicida* subsp. *masouci*	CP060030.1	SAMN14260301	PRJNA609824	GCA_014872735.1	BR19001YR	Complete	4.98	58.2	4	4335	2020-10-08T00:00:00Z
60	*A. salmonicida* subsp. *masouci*	CP049830.1	SAMN14325646	PRJNA610908	GCA_019443805.1	RZ6S-1	Complete	5.19	58.1	4	4363	2021-08-03T00:00:00Z
61	*A. salmonicida* subsp. *masouci*	CP050187.1	SAMN14351711	PRJNA611880	GCA_019443825.1	RZ6S-1	Complete	5.06	58.2	4	4189	2021-08-03T00:00:00Z
62	*A. salmonicida*	CP051883.1	SAMN14515089	PRJNA622411	GCA_012931585.1	SRW-OG1	Complete	4.62	59.0	1	4049	2020-04-29T00:00:00Z
63	*A. salmonicida* subsp. *salmonic*	CP085533.1	SAMN22480857	PRJNA773379	GCA_020683025.1	890054	Complete	5.04	58.2	9	4491	2021-10-31T00:00:00Z
64	*A. sanarellii*	CP079751.1	SAMN20179300	PRJNA746053	GCA_019334425.1	NS1	Complete	5.21	61.5	1	4201	2021-07-25T00:00:00Z
65	*A. schubertii*	CP039611.1	SAMN11129962	PRJNA527158	GCA_004919485.1	LF1708	Complete	4.35	61.4	1	3790	2019-04-28T00:00:00Z
66	*A. veronii*	AP022038.1	SAMD00194437	PRJDB6962	GCA_014168995.1	WP3-W19-ESI	Complete	4.98	58.7	6	4344	2020-07-21T23:24:00Z
67	*A. veronii*	AP022264.1	SAMD00194633	PRJDB6962	GCA_014169795.1	WP8-S18-ESB	Complete	4.91	58.7	4	4318	2020-07-21T23:55:00Z
68	*A. veronii*	AP022290.1	SAMD00194675	PRJDB6962	GCA_014169875.1	WP9-W18-ESI	Complete	4.93	58.7	5	4322	2020-07-21T23:58:00Z
69	*A. veronii*	CP002607.1	SAMN02603940	PRJNA63671	GCA_000204115.1	B565	Complete	4.55	58.7	1	4027	2011-04-13T00:00:00Z
70	*A. veronii*	CP012504.1	SAMN04012505	PRJNA293940	GCA_001593245.1	TH0426	Complete	4.92	58.3	1	4372	2016-03-16T00:00:00Z
71	*A. veronii*	CP015448.1	SAMN04901561	PRJNA319612	GCA_001634345.1	CB51	Complete	4.58	58.6	1	3623	2016-04-29T00:00:00Z
72	*A. veronii*	CP032839.1	SAMN08281233	PRJNA428153	GCA_002866885.2	FC951	Complete	4.86	58.7	2	4256	2018-10-10T00:00:00Z
73	*A. veronii*	CP028133.1	SAMN08731619	PRJNA438884	GCA_003491365.1	17ISAe	Complete	4.66	58.5	2	4063	2018-09-07T00:00:00Z
74	*A. veronii*	CP033604.1	SAMN10389897	PRJNA504296	GCA_003722175.1	MS-18-37	Complete	4.68	58.6	1	4088	2018-11-13T00:00:00Z
75	*A. veronii*	CP040717.1	SAMN11868773	PRJNA545011	GCA_009755745.1	HX3	Complete	4.76	58.5	2	4176	2019-12-16T00:00:00Z
76	*A. veronii*	CP046407.1	SAMN13220457	PRJNA587884	GCA_009833025.1	A8-AHP	Complete	4.77	58.4	4	4205	2020-01-07T00:00:00Z
77	*A. veronii*	CP047155.1	SAMN13640848	PRJNA596872	GCA_009834065.1	AVNIH1	Complete	4.81	58.5	1	4263	2020-01-08T00:00:00Z
78	*A. veronii*	CP054855.1	SAMN15184730	PRJNA638265	GCA_017310275.1	1708-29120	Complete	4.50	58.9	1	3926	2021-03-09T00:00:00Z
79	*A. veronii*	CP066125.1	SAMN17038035	PRJNA683808	GCA_020544445.1	32286	Complete	5.02	58.4	1	4444	2021-10-25T00:00:00Z
80	*A. veronii*	CP095841.1	SAMN27615187	PRJNA827608	GCA_023100745.1	AV040	Complete	4.50	58.8	1	3930	2022-04-25T00:00:00Z
81	*A. encheleia*	CP093843.1	SAMN26533773	PRJNA631893	GCA_022691185.1	H4-C21	Chromoso	4.62	61.7	1	3419	2022-03-24T00:00:00Z
82	*A. hydrophila*	CP046604.1	SAMN13507464	PRJNA594183	GCA_017310075.1	Aer_LaG34	Chromoso	4.72	61.5	1	4195	2021-03-09T00:00:00Z
83	*A. hydrophila*	CP046871.1	SAMN13540891	PRJNA594926	GCA_017310135.1	Aer_LaG33	Chromoso	4.75	61.5	1	4194	2021-03-09T00:00:00Z
84	*A. jandaei*	CP050701.1	SAMN13547657	PRJNA595181	GCA_017310175.1	Aer_Pi19.1HP/	Chromoso	4.55	58.9	2	4116	2021-03-09T00:00:00Z
85	*A. media*	CALUBH01	SAMEA110089378	PRJEB47281	GCA_943914245.1	8t8T2iAkVV_bi	Contig	4.49	61.8	73	NA	2022-06-13T00:00:00Z
86	*A. sobria*	VEMU01	SAMN11952138	PRJNA546108	GCA_008086875.1	CHT-30	Contig	4.91	57.7	343	4470	2019-08-26T00:00:00Z
87	*A. caviae*	BQVA01	SAMD00432551	PRJDB11802	GCA_022835775.1	KAM447	Contig	4.67	61.2	355	4226	2022-03-05T00:00:00Z
88	*A. veronii*	BDGY01	SAMD00057823	PRJDB5119	GCA_001748325.1	Ae52	Contig	4.56	58.7	80	NA	2016-08-24T20:43:00Z
89	*A. caviae*	BQVU01	SAMD00432571	PRJDB11802	GCA_022836175.1	KAM477	Contig	4.87	60.8	672	4389	2022-03-05T00:00:00Z
90	*A. enteropelogenes*	JAEHIG01	SAMN17083326	PRJNA685342	GCA_016730045.1	CN17A0011	Contig	4.51	59.9	21	4016	2021-01-20T00:00:00Z
91	*A. veronii*	JAEHJD01	SAMN17083364	PRJNA685342	GCA_016729285.1	CN17A0114	Contig	4.43	58.9	35	3966	2021-01-20T00:00:00Z
92	*A. caviae*	BPOK01	SAMD00294589	PRJDB11802	GCA_019972015.1	KAM375	Contig	4.83	61.3	166	4429	2021-07-14T21:41:00Z
93	*A. caviae*	BQVH01	SAMD00432558	PRJDB11802	GCA_022835915.1	KAM463	Contig	4.46	61.5	412	4012	2022-03-05T00:00:00Z
94	*A. jandaei*	JAEHJQ01	SAMN17083351	PRJNA685342	GCA_016729525.1	CN17A0084	Contig	4.51	58.9	25	4042	2021-01-20T00:00:00Z
95	*A. veronii*	VWTT01	SAMN12706443	PRJNA564235	GCA_015208715.1	A86	Contig	4.64	58.5	42	4112	2020-11-02T00:00:00Z
96	*A. hydrophila*	JAEHIV01	SAMN17083373	PRJNA685342	GCA_016729125.1	CN17A0134	Contig	4.71	61.6	24	4195	2021-01-20T00:00:00Z
97	*A. dhakensis*	JAEHIY01	SAMN17083370	PRJNA685342	GCA_016729085.1	CN17A0124	Contig	4.84	61.3	90	4292	2021-01-20T00:00:00Z
98	*A. caviae*	WUTZ01	SAMN13683804	PRJNA597794	GCA_009831085.1	ScAc2001	Contig	4.49	61.1	340	3966	2020-01-06T00:00:00Z
99	*A. veronii*	NQMB01	SAMN07488420	PRJNA396650	GCA_008119755.1	NS13	Contig	4.67	58.6	139	4212	2019-08-27T00:00:00Z
100	*A. veronii*	NPKE01	SAMN07430307	PRJNA396650	GCA_004379205.1	NS2	Contig	4.72	58.5	143	4264	2019-03-26T00:00:00Z
101	*A. veronii*	JAEHHV01	SAMN17083339	PRJNA685342	GCA_016729775.1	CN17A0049	Contig	4.30	58.9	31	3767	2021-01-20T00:00:00Z
102	*A. veronii*	JAAQQN01	SAMN14381366	PRJNA612772	GCA_012029535.1	CNRT12	Contig	4.90	58.1	479	4448	2020-04-03T00:00:00Z
103	*A. veronii*	JAEHJO01	SAMN17083353	PRJNA685342	GCA_016729495.1	CN17A0087	Contig	4.58	58.6	80	4069	2021-01-20T00:00:00Z
104	*A. veronii*	VWTV01	SAMN12706445	PRJNA564235	GCA_015208735.1	A34	Contig	4.64	58.5	82	4121	2020-11-02T00:00:00Z
105	*A. veronii*	PZKL01	SAMN08812721	PRJNA447880	GCA_003036425.1	XH.VA.1	Contig	5.36	56.5	62	4948	2018-04-05T00:00:00Z
106	*A. enteropelogenes*	JAEHHW01	SAMN17083338	PRJNA685342	GCA_016729765.1	CN17A0048	Contig	4.39	59.8	29	3915	2021-01-20T00:00:00Z
107	*A. jandaei*	RPDF01	SAMN10395257	PRJNA504557	GCA_009749285.1	82	Contig	4.76	58.5	481	4339	2019-12-13T00:00:00Z
108	*A. veronii*	CAJKQS01	SAMEA7846589	PRJEB37358	GCA_905202105.1	ERR1305902-I	Contig	4.11	59.4	226	NA	2021-02-01T00:00:00Z
109	*A. schubertii*	JAIRBS01	SAMN21302987	PRJNA761361	GCA_020089835.1	CHULA2021b	Contig	4.23	61.9	41	3905	2021-09-21T00:00:00Z
110	*A. caviae*	BPNG01	SAMD00294558	PRJDB11802	GCA_019971155.1	KAM341	Contig	4.89	60.4	526	4404	2021-07-14T22:11:00Z

111	*A. salmonicida* subsp. *smithia*	JZTI01	SAMN03396265	PRJNA264317	GCA_001466445.1	JF4097	Contig	4.31	58.7	344	3948	2015-12-15T00:00:00Z
112	*A. veronii*	JAIEYI01	SAMN20667239	PRJNA749892	GCA_021608565.1	A20-5	Contig	4.50	58.8	40	3978	2022-01-25T00:00:00Z
113	*A. veronii*	CDDU01	SAMEA2752401	PRJEB7047	GCA_000820285.1	CIP 107763	Contig	4.43	58.8	64	3989	2014-11-24T00:00:00Z
114	*A. hydrophila*	JAEHHP01	SAMN17083345	PRJNA685342	GCA_016729665.1	CN17A0062	Contig	4.72	61.5	18	4217	2021-01-20T00:00:00Z
115	*A. caviae*	JAEHJB01	SAMN17083366	PRJNA685342	GCA_016729255.1	CN17A0118	Contig	4.48	61.5	93	4027	2021-01-20T00:00:00Z
116	*A. hydrophila*	JEMK01	SAMN02643439	PRJNA183197	GCA_000586035.1	145	Contig	4.86	61.5	113	4308	2014-03-07T00:00:00Z
117	*A. veronii*	JAEHHQ01	SAMN17083344	PRJNA685342	GCA_016729675.1	CN17A0059	Contig	4.26	58.9	48	3765	2021-01-20T00:00:00Z
118	*A. caviae*	LIIX01	SAMN03995874	PRJNA292995	GCA_001270765.1	429865	Contig	4.70	61.0	4	4147	2015-08-19T00:00:00Z
119	*A. caviae*	BQVP01	SAMD00432566	PRJDB11802	GCA_022836075.1	KAM472	Contig	4.73	60.9	766	4277	2022-03-05T00:00:00Z
120	*A. dhakensis*	RQKD01	SAMN10457753	PRJNA506284	GCA_003849805.1	Aer283	Contig	4.74	61.9	182	4207	2018-11-28T00:00:00Z
121	*A. caviae*	BPNM01	SAMD00294565	PRJDB11802	GCA_019971485.1	KAM350	Contig	4.80	60.9	437	4388	2021-07-14T22:03:00Z
122	*A. veronii*	NNSE01	SAMN07375086	PRJNA396650	GCA_003367135.1	AG_5.28.6	Contig	4.61	58.6	98	4124	2018-08-07T00:00:00Z
123	*A. hydrophila*	AMQA01	SAMN02470915	PRJNA175471	GCA_000315835.1	SNUFPC-A8	Contig	4.97	60.8	41	4508	2012-11-29T00:00:00Z
124	*A. caviae*	BPOJ01	SAMD00294588	PRJDB11802	GCA_019971995.1	KAM374	Contig	4.79	61.2	217	4400	2021-07-14T21:40:00Z
125	*A. salmonicida*	UAPT01	SAMEA4530645	PRJEB6403	GCA_900445115.1	NCTC12959	Contig	4.93	58.3	10	4294	2018-06-12T00:00:00Z
126	*A. veronii*	JAIEYL01	SAMN20667242	PRJNA749892	GCA_021608475.1	A21-11	Contig	4.44	58.9	54	3956	2022-01-25T00:00:00Z
127	*A. caviae*	BQVT01	SAMD00432570	PRJDB11802	GCA_022836155.1	KAM476	Contig	4.68	60.9	528	4203	2022-03-05T00:00:00Z
128	*A. veronii*	JAIEYF01	SAMN20667236	PRJNA749892	GCA_021608585.1	A20-12	Contig	4.46	58.9	56	3978	2022-01-25T00:00:00Z
129	*A. veronii*	JAIEXZ01	SAMN20667230	PRJNA749892	GCA_021608675.1	A26	Contig	4.52	58.8	34	4016	2022-01-25T00:00:00Z
130	*A. caviae*	BPOM01	SAMD00294592	PRJDB11802	GCA_019972275.1	KAM379	Contig	4.89	61.3	163	4500	2021-07-14T21:53:00Z
131	*A. caviae*	BPOO01	SAMD00294594	PRJDB11802	GCA_019972615.1	KAM381	Contig	4.78	61.3	157	4382	2021-07-14T21:34:00Z
132	*A. hydrophila*	JFJO01	SAMN02673382	PRJNA236257	GCA_000626755.1	AD9	Contig	4.91	61.3	221	4445	2014-04-11T00:00:00Z
133	*A. caviae*	BPNL01	SAMD00294564	PRJDB11802	GCA_019971385.1	KAM348	Contig	4.81	61.3	294	4395	2021-07-14T21:45:00Z
134	*A. hydrophila*	JDWC01	SAMN02597477	PRJNA237917	GCA_000708065.1	NF2	Contig	4.79	61.3	134	4314	2014-06-16T00:00:00Z
135	*A. hydrophila*	VHIW01	SAMN12097418	PRJNA528964	GCA_019742435.1	4484	Contig	4.83	61.2	13	2153	2021-08-24T00:00:00Z
136	*A. veronii*	JAIEYJ01	SAMN20667240	PRJNA749892	GCA_021608465.1	A20-8	Contig	4.40	59.0	40	3863	2022-01-25T00:00:00Z
137	*A. veronii*	LKKB01	SAMN04099662	PRJNA296464	GCA_001446755.1	TTU2014-134A	Contig	4.68	58.6	59	4169	2015-11-19T00:00:00Z
138	*A. caviae*	BQVJ01	SAMD00432560	PRJDB11802	GCA_022835955.1	KAM466	Contig	4.68	60.9	478	4200	2022-03-05T00:00:00Z
139	*A. caviae*	JAEHJE01	SAMN17083363	PRJNA685342	GCA_016729305.1	CN17A0110	Contig	4.59	61.8	24	4098	2021-01-20T00:00:00Z
140	*A. veronii*	JAIEYG01	SAMN20667237	PRJNA749892	GCA_021608525.1	A20-14	Contig	4.59	58.5	76	4093	2022-01-25T00:00:00Z
141	*A. rivipollensis*	JAAIKZ01	SAMN14091307	PRJNA606446	GCA_010974815.1	G87	Contig	4.66	61.1	71	4118	2020-02-23T00:00:00Z
142	*A. caviae*	BQVL01	SAMD00432562	PRJDB11802	GCA_022835995.1	KAM468	Contig	4.69	60.9	470	4205	2022-03-05T00:00:00Z
143	*A. veronii*	RAWX01	SAMN10105084	PRJNA492757	GCA_003611985.1	MS 17-88	Contig	5.18	58.2	13	4677	2018-10-04T00:00:00Z
144	*A. caviae*	BQUX01	SAMD00432548	PRJDB11802	GCA_022835715.1	KAM338	Contig	5.48	60.7	296	5012	2022-03-05T00:00:00Z
145	*A. allosaccharophila*	MRZP01	SAMN05860754	PRJNA345312	GCA_001908545.1	CCM 4363	Contig	4.72	58.3	199	3922	2016-12-19T00:00:00Z
146	*A. veronii*	MRUI01	SAMN06130232	PRJNA356925	GCA_001921885.1	pamvotica	Contig	4.92	58.1	21	4366	2016-12-26T00:00:00Z
147	*A. veronii*	JAHVAR01	SAMN20179697	PRJNA746069	GCA_022875935.1	HD6451	Contig	4.92	58.7	216	4482	2022-04-11T00:00:00Z
148	*A. caviae*	BQVO01	SAMD00432565	PRJDB11802	GCA_022836055.1	KAM471	Contig	4.48	61.5	348	4047	2022-03-05T00:00:00Z
149	*A. caviae*	BPNC01	SAMD00294553	PRJDB11802	GCA_019972495.1	KAM335	Contig	4.58	61.3	318	4132	2021-07-14T21:27:00Z
150	*A. hydrophila*	JACLAO01	SAMN15641139	PRJNA648413	GCA_014397045.1	A-1	Contig	4.98	61.2	8	4390	2020-09-02T00:00:00Z
151	*A. caviae*	BPNT01	SAMD00294572	PRJDB11802	GCA_019971675.1	KAM357	Contig	4.53	61.5	276	4059	2021-07-14T21:26:00Z
152	*A. caviae*	JAEHIL01	SAMN17083383	PRJNA685342	GCA_016728885.1	CN17A0191	Contig	4.35	61.6	154	3874	2021-01-20T00:00:00Z
153	*A. caviae*	CAAKNG01	SAMEA5282775	PRJEB31025	GCA_901202955.1	Sch29	Contig	4.43	61.3	195	3970	2019-05-08T00:00:00Z
154	*A. hydrophila*	LNUR01	SAMN04253181	PRJNA301651	GCA_001535585.1	TN-97-08	Contig	5.09	60.8	16	4586	2016-01-25T00:00:00Z
155	*A. enteropelogenes*	JMGO02	SAMN02732394	PRJNA245216	GCA_000687355.2	1999lcr	Contig	4.33	60.0	18	3839	2014-05-09T00:00:00Z
156	*A. veronii*	JAEHHZ01	SAMN17083335	PRJNA685342	GCA_016729865.1	CN17A0036	Contig	4.45	58.9	38	3993	2021-01-20T00:00:00Z
157	*A. salmonicida* subsp. *salmonic*	MIIQ01	SAMN05728467	PRJNA264317	GCA_001902045.1	2009-157 K5	Contig	4.87	58.4	112	4334	2016-12-13T00:00:00Z
158	*A. caviae*	JAEHIC01	SAMN17083332	PRJNA685342	GCA_016729905.1	CN17A0028	Contig	4.42	61.8	61	3987	2021-01-20T00:00:00Z
159	*A. salmonicida*	PSZJ01	SAMN08524352	PRJNA264317	GCA_003947375.1	A308	Contig	4.97	58.2	110	4496	2018-12-14T00:00:00Z
160	*A. caviae*	JAEHHN01	SAMN17083347	PRJNA685342	GCA_016729625.1	CN17A0069	Contig	4.49	61.1	139	4011	2021-01-20T00:00:00Z
161	*A. caviae*	BPNN01	SAMD00294566	PRJDB11802	GCA_019971515.1	KAM351	Contig	4.97	60.7	320	4551	2021-07-14T22:15:00Z
162	*A. veronii*	LKJS01	SAMN04099653	PRJNA296464	GCA_001446575.1	TTU2014-115A	Contig	4.53	58.7	52	4032	2015-11-19T00:00:00Z
163	*A. hydrophila*	VHIX01	SAMN12097414	PRJNA528964	GCA_019742455.1	2961	Contig	4.84	61.5	3	3947	2021-08-24T00:00:00Z
164	*A. veronii*	JAIEYH01	SAMN20667238	PRJNA749892	GCA_021608505.1	A20-17	Contig	4.46	58.9	70	3971	2022-01-25T00:00:00Z
165	*A. caviae*	BQUY01	SAMD00432549	PRJDB11802	GCA_022835735.1	KAM344	Contig	4.63	61.1	515	4222	2022-03-05T00:00:00Z
166	*A. veronii*	LKKC01	SAMN04099663	PRJNA296464	GCA_001446775.1	TTU2014-140A	Contig	4.68	58.6	81	4170	2015-11-19T00:00:00Z
167	*A. veronii*	JAEHIF01	SAMN17083327	PRJNA685342	GCA_016729985.1	CN17A0013	Contig	4.45	58.9	49	3981	2021-01-20T00:00:00Z
168	*A. salmonicida* subsp. *salmonic*	JRYW01	SAMN03120845	PRJNA264317	GCA_000786805.1	2004-05MF26	Contig	5.02	58.3	126	4601	2014-11-25T00:00:00Z
169	*A. veronii*	NMUS01	SAMN07357136	PRJNA396650	GCA_003367105.1	PDB	Contig	4.72	58.5	141	4286	2018-08-07T00:00:00Z
170	*A. hydrophila*	MAKI01	SAMN05294042	PRJNA326907	GCA_001756325.1	M052	Contig	4.97	61.1	99	4506	2016-10-11T00:00:00Z
171	*A. caviae*	UETO01	SAMEA4743709	PRJEB27351	GCA_900491665.1	ZJ33-3	Contig	4.48	61.5	223	4031	2018-06-24T00:00:00Z
172	*A. jandaei*	JAEHIN01	SAMN17083381	PRJNA685342	GCA_016728915.1	CN17A0176	Contig	4.31	59.2	19	3855	2021-01-20T00:00:00Z
173	*A. veronii*	PPUW01	SAMN08391006	PRJNA431414	GCA_002906945.1	ML09-123	Contig	4.75	58.4	32	4230	2018-01-30T00:00:00Z
174	*A. salmonicida* subsp. *salmonic*	LNGB01	SAMN04272881	PRJNA264317	GCA_004151085.1	m11743-09	Contig	4.86	58.4	119	4412	2019-02-08T00:00:00Z
175	*A. hydrophila*	LYZH01	SAMN04967902	PRJNA321184	GCA_001705945.1	Arkansas 2010	Contig	4.97	60.9	12	4423	2016-08-17T00:00:00Z
176	*A. caviae*	JAEHIM01	SAMN17083382	PRJNA685342	GCA_016728905.1	CN17A0183	Contig	4.38	61.6	183	3889	2021-01-20T00:00:00Z
177	*A. hydrophila*	ANPN01	SAMN02471861	PRJNA183196	GCA_000350405.1	116	Contig	4.68	62.0	45	4201	2013-04-02T00:00:00Z
178	*A. caviae*	CABMOA01	SAMEA5852032	PRJEB33885	GCA_902388085.1	MGYG-HGUT-	Contig	4.48	61.8	34	4006	2019-08-12T00:00:00Z
179	*A. caviae*	JAGDEV01	SAMN17101844	PRJNA685948	GCA_018359875.1	550_SP	Contig	4.52	61.7	47	4046	2021-05-14T00:00:00Z
180	*A. caviae*	JAEHIE01	SAMN17083330	PRJNA685342	GCA_016729965.1	CN17A0024	Contig	4.40	62.0	21	3955	2021-01-20T00:00:00Z
181	*A. caviae*	BPOE01	SAMD00294583	PRJDB11802	GCA_019971895.1	KAM368	Contig	4.52	61.5	290	4053	2021-07-14T21:25:00Z
182	*A. veronii*	JAAQQP01	SAMN14381462	PRJNA612772	GCA_012029575.1	NK02	Contig	4.80	58.2	400	4372	2020-04-03T00:00:00Z
183	*A. caviae*	JAEHHY01	SAMN17083336	PRJNA685342	GCA_016729825.1	CN17A0038	Contig	4.44	61.8	49	3973	2021-01-20T00:00:00Z
184	*A. jandaei*	JWJR01	SAMN03265386	PRJNA270278	GCA_000813485.1	L14h	Contig	4.68	61.7	114	4187	2015-01-02T00:00:00Z
185	*A. veronii*	JAEHJG01	SAMN17083361	PRJNA685342	GCA_016729355.1	CN17A0103	Contig	4.43	58.8	102	3892	2021-01-20T00:00:00Z
186	*A. salmonicida* subsp. *achromo*	VCSD01	SAMN11836204	PRJNA264317	GCA_006044035.1	23056	Contig	4.37	58.7	297	3783	2019-06-05T00:00:00Z
187	*A. caviae*	BPOR01	SAMD00294597	PRJDB11802	GCA_019972675.1	KAM384	Contig	4.65	61.3	291	4221	2021-07-14T21:31:00Z
188	*A. popoffii*	JAGRZL01	SAMN18686274	PRJNA720925	GCA_018138755.1	ID682	Contig	4.68	58.6	128	4151	2021-04-24T00:00:00Z
189	*A. caviae*	PUTR01	SAMN08519207	PRJNA433857	GCA_003322775.1	AFG_SD01_1	Contig	4.50	61.1	228	3996	2018-07-15T00:00:00Z
190	*A. caviae*	JAGDEX01	SAMN17101846	PRJNA685948	GCA_018359805.1	552_SP	Contig	4.46	61.7	41	4011	2021-05-14T00:00:00Z
191	*A. salmonicida* subsp. *achromo*	VCSA01	SAMN11836207	PRJNA264317	GCA_006044075.1	23055	Contig	4.42	58.7	305	3835	2019-06-05T00:00:00Z
192	*A. jandaei*	RQKB01	SAMN10457755	PRJNA506284	GCA_003849685.1	Aer337	Contig	4.71	58.6	101	4306	2018-11-28T00:00:00Z
193	*A. dhakensis*	JAEHJJ01	SAMN17083358	PRJNA685342	GCA_016729405.1	CN17A0100	Contig	4.76	61.7	36	4226	2021-01-20T00:00:00Z
194	*A. veronii*	LKJP01	SAMN04099650	PRJNA296464	GCA_001446505.1	TTU2014-108A	Contig	4.53	58.7	58	4034	2015-11-19T00:00:00Z
195	*A. hydrophila*	LXME01	SAMN04939662	PRJNA320461	GCA_001717755.1	2JBN101	Contig	5.09	60.8	47	4545	2016-08-31T00:00:00Z
196	*A. veronii*	JACEGL01	SAMN15587301	PRJNA525849	GCA_013697145.1	C198	Contig	4.58	58.6	3	3995	2020-07-26T00:00:00Z
197	*A. hydrophila*	JACLAN01	SAMN15641141	PRJNA648413	GCA_014396965.1	B-1	Contig	4.92	61.0	23	801	2020-09-02T00:00:00Z
198	*A. veronii*	JAIEYM01	SAMN20667243	PRJNA749892	GCA_021608425.1	A21-13	Contig	4.37	58.9	55	3873	2022-01-25T00:00:00Z
199	*A. veronii*	JALJOG01	SAMN26746239	PRJNA817062	GCA_022953355.1	NBCF28	Contig	4.62	58.7	87	4195	2022-04-12T00:00:00Z
200	*A. media*	JRBG01	SAMN03018687	PRJNA260227	GCA_000754925.1	ARB20	Contig	4.62	61.0	185	4126	2014-09-16T00:00:00Z
201	*A. salmonicida*	NQMJ01	SAMN07491057	PRJNA264317	GCA_002883135.1	M18076-11	Contig	4.43	58.7	228	4014	2018-01-16T00:00:00Z
202	*A. media*	JRBF01	SAMN03018686	PRJNA260228	GCA_000754915.1	ARB13	Contig	4.61	61.0	180	4124	2014-09-16T00:00:00Z
203	*A. hydrophila*	AOBO01	SAMN02471855	PRJNA183199	GCA_000354635.1	187	Contig	4.78	61.6	59	4313	2013-04-06T00:00:00Z
204	*A. hydrophila*	AOBP01	SAMN02471868	PRJNA183201	GCA_000354655.1	259	Contig	4.70	61.7	80	4219	2013-04-06T00:00:00Z
205	*A. hydrophila*	WOCB01	SAMN13387565	PRJNA591768	GCA_018094775.1	LaG38	Contig	4.75	61.5	13	4187	2021-04-19T00:00:00Z
206	*A. veronii*	JALJOH01	SAMN26746686	PRJNA817067	GCA_022953315.1	NPSF15	Contig	4.50	58.7	56	4025	2022-04-12T00:00:00Z
207	*A. caviae*	BPNA01	SAMD00294551	PRJDB11802	GCA_019972535.1	KAM333	Contig	4.57	61.2	472	4142	2021-07-14T21:28:00Z
208	*A. dhakensis*	JAEHJI01	SAMN17083359	PRJNA685342	GCA_016729375.1	CN17A0101	Contig	4.88	61.4	78	4315	2021-01-20T00:00:00Z
209	*A. hydrophila*	JPEH01	SAMN02892980	PRJNA253773	GCA_000724965.1	RB-AH	Contig	5.09	60.8	3	4571	2014-07-11T00:00:00Z
210	*A. veronii*	LXJN01	SAMN04914378	PRJNA320014	GCA_001696435.1	VBF557	Contig	4.70	58.4	526	3325	2016-07-28T00:00:00Z
211	*A. hydrophila*	JAEHHS01	SAMN17083342	PRJNA685342	GCA_016729685.1	CN17A0055	Contig	4.72	61.5	20	4220	2021-01-20T00:00:00Z
212	*A. caviae*	BPOL01	SAMD00294591	PRJDB11802	GCA_019972035.1	KAM378	Contig	4.82	61.2	235	4396	2021-07-14T21:56:00Z
213	*A. veronii*	JAEHOJ01	SAMN17083368	PRJNA685342	GCA_016729245.1	CN17A0120	Contig	4.52	58.6	128	4045	2021-01-20T00:00:00Z
214	*A. dhakensis*	WNLF01	SAMN13353935	PRJNA591201	GCA_018094645.1	Pi16.2MC	Contig	4.87	61.5	16	4286	2021-04-19T00:00:00Z
215	*A. hydrophila* subsp. *hydrophila*	JAFIMN01	SAMN18022882	PRJNA703737	GCA_018682315.1	HIa5mS2	Contig	4.97	61.2	108	4424	2021-06-02T00:00:00Z
216	*A. hydrophila*	JAEHJS01	SAMN17083349	PRJNA685342	GCA_016729605.1	CN17A0078	Contig	4.68	61.7	17	4143	2021-01-20T00:00:00Z
217	*A. hydrophila*	WOCC01	SAMN13389033	PRJNA591795	GCA_018094805.1	OnP22	Contig	4.78	61.5	10	4207	2021-04-19T00:00:00Z
218	*A. veronii*	LKKG01	SAMN04099667	PRJNA296464	GCA_001446855.1	TTU2014-143A	Contig	4.68	58.6	59	4168	2015-11-19T00:00:00Z
219	*A. caviae*	JAEHIJ01	SAMN17083323	PRJNA685342	GCA_016730095.1	CN17A0006	Contig	4.58	61.0	199	4118	2021-01-20T00:00:00Z
220	*A. caviae*	BPNK01	SAMD00294563	PRJDB11802	GCA_019971305.1	KAM347	Contig	4.81	60.9	527	4415	2021-07-14T22:06:00Z
221	*A. jandaei*	JFDL01	SAMN02597482	PRJNA237126	GCA_000708125.1	Riv2	Contig	4.48	59.0	43	4042	2014-06-16T00:00:00Z
222	*A. hydrophila*	LRRV01	SAMN04274131	PRJNA302467	GCA_001604835.1	AL09-79	Contig	4.97	60.9	13	4420	2016-03-31T00:00:00Z
223	*A. salmonicida*	JZTG01	SAMN03395036	PRJNA264317	GCA_001466435.1	Y567	Contig	4.55	58.9	47	4072	2015-12-15T00:00:00Z

224	*A. caviae*	BPNY01	SAMD00294577	PRJDB11802	GCA_019971775.1	KAM362	Contig	4.86	61.2	251	4446	2021-07-14T22:00:00Z
225	*A. veronii*	JALJOI01	SAMN26746759	PRJNA817070	GCA_022953335.1	CBSB03	Contig	4.62	58.6	78	4147	2022-04-12T00:00:00Z
226	*A. hydrophila*	JDWB01	SAMN02597476	PRJNA237913	GCA_000708105.1	NF1	Contig	4.81	61.1	150	4317	2014-06-16T00:00:00Z
227	*A. dhakensis*	BAFL01	SAMD00036618	PRJDB70	GCA_000315195.1	AAK1	Contig	4.77	61.8	37	4225	2012-04-17T00:00:00Z
228	*A. caviae*	JAEHJL01	SAMN17083356	PRJNA685342	GCA_016729435.1	CN17A0095	Contig	4.31	61.8	83	3834	2021-01-20T00:00:00Z
229	*A. veronii*	LKKF01	SAMN04099666	PRJNA296464	GCA_001446835.1	TTU2014-142A	Contig	4.68	58.6	45	4167	2015-11-19T00:00:00Z
230	*A. caviae*	BQVX01	SAMD00432574	PRJDB11802	GCA_022836235.1	KAM480	Contig	4.72	60.8	575	4276	2022-03-05T00:00:00Z
231	*A. salmonicida* subsp. *salmonic*	LSGX01	SAMN04450000	PRJNA310296	GCA_001643355.1	J227	Contig	4.70	58.5	122	4182	2016-05-13T00:00:00Z
232	*A. veronii*	JAIEYS01	SAMN20667249	PRJNA749892	GCA_021608285.1	A21-5	Contig	4.64	58.8	48	4178	2022-01-25T00:00:00Z
233	*A. veronii*	CAAKNM01	SAMEA5282783	PRJEB31025	GCA_901212395.1	KLG8	Contig	4.59	58.6	76	4143	2019-05-08T00:00:00Z
234	*A. veronii*	JAEHIS01	SAMN17083376	PRJNA685342	GCA_016728995.1	CN17A0154	Contig	4.44	59.0	33	3938	2021-01-20T00:00:00Z
235	*A. caviae*	BPOH01	SAMD00294586	PRJDB11802	GCA_019971955.1	KAM372	Contig	4.78	61.3	153	4375	2021-07-14T21:32:00Z
236	*A. salmonicida*	CAACYG01	SAMEA104338356	PRJEB6403	GCA_900683655.1	3012STDY712	Contig	4.83	58.5	22	4281	2019-02-20T00:00:00Z
237	*A. veronii*	JAAQQO01	SAMN14381434	PRJNA612772	GCA_012029545.1	NK01	Contig	4.56	58.5	95	4022	2020-04-03T00:00:00Z
238	*A. veronii*	VWTW01	SAMN12706446	PRJNA564235	GCA_015208655.1	A31	Contig	4.64	58.5	84	4124	2020-11-02T00:00:00Z
239	*A. veronii*	NKWN01	SAMN07312765	PRJNA391781	GCA_006246325.1	CECT 4902	Scaffold	4.64	58.4	29	4112	2019-06-11T00:00:00Z
240	*A. veronii*	NKXC01	SAMN07312748	PRJNA391781	GCA_006246485.1	AMC 25	Scaffold	4.60	58.8	50	4152	2019-06-11T00:00:00Z
241	*A. allosaccharophila*	UETN01	SAMEA4743713	PRJEB27351	GCA_900491725.1	Z9-6	Scaffold	4.59	58.8	66	4057	2018-06-24T00:00:00Z
242	*A. dhakensis*	JAACNA01	SAMN13343730	PRJNA590952	GCA_019348695.1	26M	Scaffold	4.90	61.4	26	4354	2021-07-26T00:00:00Z
243	*A. salmonicida* subsp. *salmonic*	JYFF01	SAMN03332961	PRJNA264317	GCA_001499805.2	RS534	Scaffold	4.89	58.2	123	4397	2015-12-15T00:00:00Z
244	*A. hydrophila*	JAABKF01	SAMN13424805	PRJNA592442	GCA_019399965.1	Brac54	Scaffold	4.75	61.5	22	4217	2021-07-29T00:00:00Z
245	*A. salmonicida*	LUHO01	SAMN04529529	PRJNA314146	GCA_002093695.1	BG	Scaffold	4.79	58.6	236	4325	2017-04-14T00:00:00Z
246	*A. veronii*	NKVZ01	SAMN07312779	PRJNA391781	GCA_006243165.1	TCO21	Scaffold	4.46	58.7	93	3983	2019-06-11T00:00:00Z
247	*A. salmonicida*	UETH01	SAMEA4743712	PRJEB27351	GCA_900491655.1	Z5-5	Scaffold	4.71	58.6	110	4195	2018-06-24T00:00:00Z
248	*A. veronii*	CDBQ01	SAMEA2752397	PRJEB7051	GCA_000820385.1	LMG 13067	Scaffold	4.74	58.4	72	4228	2014-11-24T00:00:00Z
249	*A. salmonicida*	NKWL01	SAMN07312767	PRJNA391781	GCA_006246135.1	CIP 103210	Scaffold	4.54	58.7	238	3944	2019-06-11T00:00:00Z
250	*A. dhakensis*	NKWQ01	SAMN07312762	PRJNA391781	GCA_006246145.1	BVH68	Scaffold	4.86	61.7	82	4418	2019-06-11T00:00:00Z
251	*A. bivalvium*	NXBQ01	SAMN07680236	PRJNA408193	GCA_003265465.1	ZJ19-2	Scaffold	4.26	62.4	38	3794	2018-06-26T00:00:00Z
252	*A. caviae*	JAAALU01	SAMN13763659	PRJNA600002	GCA_009906325.1	AK245	Scaffold	4.45	61.6	203	3975	2020-01-19T00:00:00Z
253	*A. jandaei*	NKWG01	SAMN07312772	PRJNA391781	GCA_006243385.1	Ho603	Scaffold	4.63	58.5	186	4174	2019-06-11T00:00:00Z
254	*A. caviae*	CDBK01	SAMEA2752423	PRJEB7024	GCA_000819785.1	CECT 838	Scaffold	4.47	61.7	111	4004	2014-11-24T00:00:00Z
255	*A. veronii*	NKWS01	SAMN07312759	PRJNA391781	GCA_006246165.1	BVH46	Scaffold	4.51	58.8	39	4033	2019-06-11T00:00:00Z
256	*A. hydrophila*	MRDF01	SAMN06112127	PRJNA356344	GCA_001937135.1	HZAUAH	Scaffold	5.04	60.9	32	4475	2017-01-05T00:00:00Z
257	*A. veronii*	JAACNB01	SAMN13343884	PRJNA590960	GCA_019348625.1	Pi6.2MC	Scaffold	4.74	58.5	51	4221	2021-07-26T00:00:00Z
258	*A. jandaei*	JAAKGH01	SAMN14124565	PRJNA607235	GCA_016805365.1	Aer_On5M	Scaffold	4.51	59.0	34	4018	2021-02-03T00:00:00Z
259	*A. caviae*	JAAALX01	SAMN13763656	PRJNA600002	GCA_009906335.1	ADV118	Scaffold	4.39	61.5	140	3921	2020-01-19T00:00:00Z
260	*A. veronii*	NKXE01	SAMN07312746	PRJNA391781	GCA_006246235.1	AK241	Scaffold	4.60	58.6	42	4115	2019-06-11T00:00:00Z
261	*A. media*	UETL01	SAMEA4743710	PRJEB27351	GCA_900491715.1	Z1-6	Scaffold	4.52	61.4	131	4008	2018-06-24T00:00:00Z
262	*A. dhakensis*	NKWP01	SAMN07312763	PRJNA391781	GCA_006246345.1	BVH69	Scaffold	4.80	61.7	61	4354	2019-06-11T00:00:00Z
263	*A. hydrophila*	JVDW01	SAMN03197714	PRJNA267549	GCA_001057115.1	52_AHYD	Scaffold	4.68	61.6	111	4180	2015-07-10T00:00:00Z
264	*A. hydrophila*	JVCD01	SAMN03197759	PRJNA267549	GCA_001057275.1	56_AHYD	Scaffold	4.68	61.6	99	4183	2015-07-10T00:00:00Z
265	*A. hydrophila*	JAABKG01	SAMN13412801	PRJNA592155	GCA_019399995.1	Pi11	Scaffold	4.77	61.4	40	4218	2021-07-29T00:00:00Z
266	*A. jandaei*	PRCV01	SAMN08457750	PRJNA432735	GCA_002925765.1	IMET J	Scaffold	4.73	58.4	20	4273	2018-02-12T00:00:00Z
267	*A. veronii*	LKJQ01	SAMN04099651	PRJNA296464	GCA_001446535.1	TTU2014-113A	Scaffold	4.66	58.6	122	4167	2015-11-19T00:00:00Z
268	*A. veronii*	NKXD01	SAMN07312747	PRJNA391781	GCA_006246495.1	AK247	Scaffold	4.55	58.8	36	4077	2019-06-11T00:00:00Z
269	*A. dhakensis*	NKWO01	SAMN07312764	PRJNA391781	GCA_006243335.1	BVH70	Scaffold	4.72	61.7	59	4247	2019-06-11T00:00:00Z
270	*A. salmonicida* subsp. *salmonic*	CDDW01	SAMEA2752412	PRJEB7036	GCA_000820065.1	CIP 103209	Scaffold	4.74	58.5	128	4260	2014-11-24T00:00:00Z
271	*A. bivalvium*	NXBX01	SAMN07680229	PRJNA408193	GCA_003265565.1	ZJ20-2	Scaffold	4.30	62.2	84	3829	2018-06-26T00:00:00Z
272	*A. caviae*	QKOU01	SAMN09289754	PRJNA472583	GCA_004024475.1	GEO_47_Up_A	Scaffold	4.51	61.6	261	4086	2019-01-15T00:00:00Z
273	*A. sobria*	NKWE01	SAMN07312774	PRJNA391781	GCA_006243315.1	PAQ091014-5	Scaffold	4.66	57.7	101	4168	2019-06-11T00:00:00Z
274	*A. veronii*	DDJB01	SAMN06453457	PRJNA348753	GCA_002339005.1	UBA1835	Scaffold	4.11	59.0	323	NA	2017-09-22T00:00:00Z
275	*A. hydrophila*	JAABKI01	SAMN13427745	PRJNA592528	GCA_019399935.1	Aer_Brac46	Scaffold	4.90	61.3	48	4318	2021-07-29T00:00:00Z
276	*A. salmonicida*	PSZK01	SAMN08524351	PRJNA264317	GCA_003947395.1	947C	Scaffold	4.78	58.4	48	4317	2018-12-14T00:00:00Z
277	*A. hydrophila*	DBMF01	SAMN06453168	PRJNA348753	GCA_002297625.1	UBA705	Scaffold	4.25	61.6	282	NA	2017-09-12T00:00:00Z
278	*A. caviae*	QKPH01	SAMN09289741	PRJNA472583	GCA_004024325.1	GEO_23_Dow	Scaffold	4.93	60.8	250	4563	2019-01-15T00:00:00Z
279	*A. veronii*	AGWU01	SAMN02463949	PRJNA71515	GCA_000298015.1	AMC34	Scaffold	4.58	58.4	1	4084	2012-09-17T00:00:00Z
280	*A. salmonicida*	JZTH01	SAMN03395037	PRJNA264317	GCA_001481545.2	Y577	Scaffold	4.74	58.6	104	4225	2015-12-15T00:00:00Z
281	*A. veronii*	NKXG01	SAMN07312744	PRJNA391781	GCA_006246555.1	AK227	Scaffold	4.40	58.7	67	3953	2019-06-11T00:00:00Z
282	*A. dhakensis*	JAGFEC01	SAMN17860440	PRJNA701275	GCA_017582425.1	16006	Scaffold	4.88	61.8	160	4348	2021-03-24T00:00:00Z
283	*A. dhakensis*	AGWR01	SAMN02463947	PRJNA71509	GCA_000298055.1	SSU	Scaffold	4.94	61.5	2	4431	2012-09-17T00:00:00Z
284	*A. caviae*	NXBR01	SAMN07680235	PRJNA408193	GCA_003265475.1	ZJ17-2	Scaffold	4.56	61.3	256	4121	2018-06-26T00:00:00Z
285	*A. hydrophila*	NKWD01	SAMN07312775	PRJNA391781	GCA_006243275.1	PAQ091014-9	Scaffold	4.93	60.9	72	4431	2019-06-11T00:00:00Z
286	*A. veronii*	NKXH01	SAMN07312743	PRJNA391781	GCA_006246275.1	ADV102	Scaffold	4.52	58.6	87	4086	2019-06-11T00:00:00Z
287	*A. sobria*	NKXA01	SAMN07312750	PRJNA391781	GCA_006246215.1	ARS-145-14	Scaffold	4.79	57.6	100	4304	2019-06-11T00:00:00Z
288	*A. hydrophila*	NKWF01	SAMN07312773	PRJNA391781	GCA_006243365.1	PAQ091014-1	Scaffold	4.94	60.9	114	4452	2019-06-11T00:00:00Z
289	*A. veronii*	RZII01	SAMN10644079	PRJNA511801	GCA_003990305.1	CQ-AV1	Scaffold	4.78	58.5	36	4328	2019-01-01T00:00:00Z
290	*A. rivipollensis*	JAAILB01	SAMN14091305	PRJNA606446	GCA_010974915.1	G42	Scaffold	4.58	61.3	55	4099	2020-02-23T00:00:00Z
291	*A. caviae*	JAKCNV01	SAMN24245012	PRJNA762299	GCA_021440765.1	INSAq239	Scaffold	4.37	61.3	363	3992	2022-01-11T00:00:00Z
292	*A. caviae*	MDSC01	SAMN05525171	PRJNA338160	GCA_001730205.1	CHZ306	Scaffold	4.79	60.7	65	4180	2016-09-16T00:00:00Z
293	*A. hydrophila*	MJGE01	SAMN05791072	PRJNA343595	GCA_014839605.1	4LNC202	Scaffold	5.00	60.9	32	4454	2020-09-30T00:00:00Z
294	*A. caviae*	NXBP01	SAMN07680237	PRJNA408193	GCA_003265425.1	ZJ66-1	Scaffold	4.39	61.5	150	3944	2018-06-26T00:00:00Z
295	*A. veronii*	JACGXR01	SAMN15693637	PRJNA650139	GCA_015318285.1	XhG1.2	Scaffold	4.57	58.7	34	4041	2020-11-09T00:00:00Z
296	*A. veronii*	AGWW01	SAMN02463951	PRJNA71519	GCA_000298035.1	AMC35	Scaffold	4.57	58.5	2	3980	2012-09-17T00:00:00Z
297	*A. veronii*	VZQA01	SAMN12777764	PRJNA566091	GCA_008802305.1	D	Scaffold	4.43	59.0	149	3962	2019-10-01T00:00:00Z
298	*A. caviae*	JAAALW01	SAMN13763657	PRJNA600002	GCA_009906375.1	BVH84	Scaffold	4.48	61.3	151	3988	2020-01-19T00:00:00Z
299	*A. caviae*	BQVV01	SAMD00432572	PRJDB11802	GCA_022836195.1	KAM478	Contig	4.73	60.9	490	4245	2022-03-05T00:00:00Z
300	*A. veronii*	CDBU01	SAMEA2752398	PRJEB7050	GCA_000820365.1	CECT 4486	Scaffold	4.41	58.9	66	3943	2014-11-24T00:00:00Z

Supplementary Table 3. Dice index between *Aeromonas* genomes

NCBI species name (originally designated species)	Re-identified species name based on phylogenetic analysis and OrthoANI.	Serial no.	ACC.no	66 AP022038.1 *A. veronii* / *A. allosaccharophila*	67 AP022264.1 *A. veronii* / *A. allosaccharophila*	68 AP022290.1 *A. veronii* / *A. allosaccharophila*	145 MRZP01 *A. allosaccharophila* / *A. allosaccharophila*	241 UETN01 *A. allosaccharophila* / *A. allosaccharophila*	reference NKWZ01 *A. allosaccharophila* / *A. allosaccharophila*	reference JRGL01 *A. aquatica* / *A. aquatica*	reference CDDH01 *A. australiensis* / *A. australiensis*	reference CDDA01 *A. bestiarum* / *A. bestiarum*	251 NXBQ01 *A. bivalvium* / *A. bivalvium*	271 NXBX01 *A. bivalvium* / *A. bivalvium*	reference CDBT01 *A. bivalvium* / *A. bivalvium*	2 AP019195.1 *A. caviae* / *A. caviae*	3 AP022214.1 *A. caviae* / *A. caviae*	4 AP022241.1 *A. caviae* / *A. caviae*	5 CP062787.1 *A. caviae* / *A. caviae*	6 CP024198.1 *A. caviae* / *A. caviae*	7 CP039832.1 *A. caviae* / *A. caviae*	8 CP047982.1 *A. caviae* / *A. caviae*	9 CP047983.1 *A. caviae* / *A. caviae*	10 CP065937.1 *A. caviae* / *A. caviae*	11 CP066813.1 *A. caviae* / *A. caviae*
A. veronii	*A. allosaccharophila*	66	AP022038.1	1.00	0.79	0.83	0.78	0.81	0.81	0.55	0.68	0.58	0.50	0.50	0.49	0.57	0.56	0.57	0.55	0.55	0.56	0.56	0.56	0.55	0.56
A. veronii	*A. allosaccharophila*	67	AP022264.1	0.79	1.00	0.80	0.79	0.80	0.81	0.55	0.68	0.58	0.49	0.49	0.49	0.55	0.57	0.55	0.56	0.54	0.54	0.56	0.54	0.53	0.56
A. veronii	*A. allosaccharophila*	68	AP022290.1	0.83	0.80	1.00	0.80	0.82	0.82	0.56	0.69	0.60	0.51	0.51	0.50	0.57	0.58	0.57	0.57	0.56	0.58	0.57	0.57	0.56	0.57
A. allosaccharophila	*A. allosaccharophila*	145	MRZP01	0.78	0.79	0.80	1.00	0.79	0.80	0.55	0.69	0.59	0.50	0.50	0.50	0.56	0.57	0.56	0.57	0.56	0.56	0.56	0.55	0.55	0.57
A. allosaccharophila	*A. allosaccharophila*	241	UETN01	0.81	0.80	0.82	0.79	1.00	0.83	0.56	0.70	0.60	0.51	0.51	0.51	0.57	0.57	0.57	0.56	0.56	0.56	0.57	0.55	0.55	0.57
A. allosaccharophila	*A. allosaccharophila*	reference	NKWZ01	0.81	0.81	0.82	0.80	0.83	1.00	0.56	0.70	0.60	0.50	0.50	0.50	0.56	0.57	0.56	0.56	0.56	0.55	0.56	0.55	0.54	0.57
A. aquatica	*A. aquatica*	reference	JRGL01	0.55	0.55	0.56	0.55	0.56	0.56	1.00	0.54	0.66	0.54	0.54	0.53	0.67	0.69	0.67	0.68	0.69	0.67	0.67	0.67	0.65	0.68
A. australiensis	*A. australiensis*	reference	CDDH01	0.68	0.68	0.69	0.69	0.70	0.70	0.54	1.00	0.56	0.51	0.51	0.51	0.55	0.55	0.55	0.54	0.54	0.54	0.55	0.53	0.52	0.55
A. bestiarum	*A. bestiarum*	reference	CDDA01	0.58	0.58	0.60	0.59	0.60	0.60	0.66	0.56	1.00	0.54	0.54	0.54	0.64	0.65	0.64	0.65	0.65	0.63	0.64	0.62	0.62	0.65
A. bivalvium	*A. bivalvium*	251	NXBQ01	0.50	0.49	0.51	0.50	0.51	0.50	0.54	0.51	0.54	1.00	0.99	0.89	0.59	0.60	0.59	0.59	0.59	0.60	0.59	0.58	0.57	0.60
A. bivalvium	*A. bivalvium*	271	NXBX01	0.50	0.49	0.51	0.50	0.51	0.50	0.54	0.51	0.54	0.99	1.00	0.89	0.59	0.60	0.59	0.59	0.59	0.59	0.59	0.58	0.57	0.60
A. bivalvium	*A. bivalvium*	reference	CDBT01	0.49	0.49	0.50	0.50	0.51	0.50	0.53	0.51	0.54	0.89	0.89	1.00	0.58	0.59	0.58	0.59	0.59	0.58	0.59	0.57	0.56	0.59
A. caviae	*A. caviae*	2	AP019195.1	0.57	0.55	0.57	0.56	0.57	0.56	0.67	0.55	0.64	0.59	0.59	0.58	1.00	0.91	1.00	0.87	0.87	0.88	0.91	0.87	0.86	0.89
A. caviae	*A. caviae*	3	AP022214.1	0.56	0.57	0.58	0.57	0.57	0.57	0.69	0.55	0.65	0.60	0.60	0.59	0.91	1.00	0.91	0.89	0.89	0.89	0.93	0.89	0.86	0.91
A. caviae	*A. caviae*	4	AP022241.1	0.57	0.55	0.57	0.56	0.57	0.56	0.67	0.55	0.64	0.59	0.59	0.58	1.00	0.91	1.00	0.87	0.87	0.88	0.91	0.87	0.86	0.89
A. caviae	*A. caviae*	5	CP062787.1	0.55	0.56	0.57	0.57	0.56	0.56	0.68	0.54	0.65	0.59	0.59	0.59	0.87	0.89	0.87	1.00	0.90	0.87	0.88	0.85	0.83	0.88
A. caviae	*A. caviae*	6	CP024198.1	0.55	0.54	0.56	0.56	0.56	0.56	0.69	0.54	0.65	0.59	0.59	0.59	0.87	0.89	0.87	0.90	1.00	0.87	0.88	0.86	0.83	0.88
A. caviae	*A. caviae*	7	CP039832.1	0.56	0.54	0.58	0.56	0.56	0.55	0.67	0.54	0.63	0.60	0.59	0.58	0.88	0.89	0.88	0.87	0.87	1.00	0.88	0.87	0.84	0.88
A. caviae	*A. caviae*	8	CP047982.1	0.56	0.56	0.57	0.56	0.57	0.56	0.67	0.55	0.64	0.59	0.59	0.59	0.91	0.93	0.91	0.88	0.88	0.88	1.00	0.90	0.85	0.89
A. caviae	*A. caviae*	9	CP047983.1	0.56	0.54	0.57	0.55	0.55	0.55	0.67	0.53	0.62	0.58	0.58	0.57	0.87	0.89	0.87	0.85	0.86	0.87	0.90	1.00	0.84	0.87
A. caviae	*A. caviae*	10	CP065937.1	0.55	0.53	0.56	0.55	0.55	0.54	0.65	0.52	0.62	0.57	0.57	0.56	0.86	0.86	0.86	0.83	0.83	0.84	0.85	0.84	1.00	0.85
A. caviae	*A. caviae*	11	CP066813.1	0.56	0.56	0.57	0.57	0.57	0.57	0.68	0.55	0.65	0.60	0.60	0.59	0.89	0.91	0.89	0.88	0.88	0.88	0.89	0.87	0.85	1.00
A. caviae	*A. caviae*	12	CP084350.1	0.56	0.56	0.57	0.57	0.57	0.57	0.69	0.55	0.65	0.59	0.59	0.59	0.89	0.91	0.89	0.91	0.90	0.88	0.89	0.86	0.84	0.89
A. caviae	*A. caviae*	13	CP085468.1	0.56	0.56	0.57	0.56	0.57	0.56	0.67	0.55	0.64	0.59	0.59	0.59	0.89	0.90	0.89	0.86	0.86	0.88	0.90	0.87	0.85	0.89
A. caviae	*A. caviae*	87	BQVA01	0.55	0.54	0.56	0.55	0.55	0.54	0.66	0.53	0.62	0.58	0.57	0.57	0.88	0.90	0.88	0.85	0.85	0.85	0.88	0.85	0.85	0.87
A. caviae	*A. caviae*	89	BQVU01	0.55	0.53	0.56	0.54	0.54	0.54	0.65	0.53	0.62	0.57	0.56	0.56	0.86	0.86	0.86	0.82	0.82	0.84	0.85	0.84	0.83	0.85
A. caviae	*A. caviae*	92	BPOK01	0.53	0.53	0.55	0.54	0.53	0.53	0.64	0.51	0.61	0.57	0.57	0.56	0.82	0.85	0.82	0.85	0.85	0.84	0.83	0.82	0.81	0.83
A. caviae	*A. caviae*	93	BQVH01	0.56	0.55	0.57	0.56	0.57	0.56	0.68	0.55	0.64	0.59	0.59	0.58	0.91	0.91	0.91	0.87	0.88	0.87	0.90	0.87	0.85	0.89
A. caviae	*A. caviae*	98	WUTZ01	0.55	0.54	0.55	0.55	0.55	0.54	0.64	0.53	0.62	0.56	0.57	0.56	0.84	0.85	0.84	0.82	0.82	0.83	0.83	0.82	0.81	0.84
A. caviae	*A. caviae*	110	BPNG01	0.56	0.54	0.56	0.55	0.55	0.54	0.65	0.52	0.62	0.57	0.56	0.56	0.84	0.85	0.84	0.83	0.82	0.84	0.84	0.83	0.82	0.86
A. caviae	*A. caviae*	115	JAEHJB01	0.56	0.55	0.57	0.56	0.56	0.56	0.68	0.54	0.64	0.60	0.60	0.59	0.88	0.89	0.88	0.89	0.89	0.89	0.88	0.87	0.85	0.89
A. caviae	*A. caviae*	118	LIIX01	0.56	0.54	0.57	0.55	0.56	0.55	0.66	0.53	0.63	0.59	0.59	0.57	0.90	0.90	0.90	0.86	0.86	0.88	0.90	0.87	0.86	0.89
A. caviae	*A. caviae*	119	BQVP01	0.55	0.53	0.56	0.54	0.55	0.54	0.65	0.53	0.62	0.57	0.57	0.56	0.86	0.86	0.86	0.83	0.83	0.85	0.85	0.84	0.83	0.86
A. caviae	*A. caviae*	121	BPNM01	0.55	0.54	0.56	0.54	0.55	0.54	0.64	0.52	0.62	0.57	0.57	0.56	0.86	0.86	0.86	0.83	0.82	0.84	0.85	0.84	0.83	0.86
A. caviae	*A. caviae*	124	BPOJ01	0.54	0.54	0.56	0.54	0.54	0.54	0.65	0.52	0.62	0.57	0.57	0.57	0.83	0.85	0.83	0.85	0.85	0.85	0.84	0.82	0.82	0.84
A. caviae	*A. caviae*	127	BQVT01	0.56	0.54	0.57	0.55	0.55	0.55	0.65	0.53	0.62	0.57	0.57	0.56	0.87	0.87	0.87	0.83	0.83	0.85	0.85	0.85	0.83	0.86
A. caviae	*A. caviae*	130	BPOM01	0.53	0.52	0.55	0.54	0.53	0.53	0.64	0.51	0.61	0.56	0.56	0.56	0.82	0.84	0.82	0.84	0.84	0.84	0.83	0.82	0.81	0.83
A. caviae	*A. caviae*	131	BPOO01	0.54	0.53	0.56	0.55	0.54	0.54	0.65	0.52	0.62	0.57	0.57	0.57	0.83	0.85	0.83	0.85	0.85	0.85	0.84	0.83	0.81	0.84
A. caviae	*A. caviae*	133	BPNL01	0.54	0.54	0.56	0.54	0.55	0.54	0.65	0.52	0.61	0.56	0.56	0.56	0.85	0.86	0.85	0.83	0.83	0.84	0.86	0.84	0.83	0.86
A. caviae	*A. caviae*	138	BQVJ01	0.56	0.54	0.57	0.55	0.55	0.55	0.66	0.53	0.63	0.57	0.57	0.57	0.87	0.87	0.87	0.84	0.84	0.85	0.86	0.85	0.83	0.87
A. caviae	*A. caviae*	139	JAEHJE01	0.54	0.55	0.56	0.56	0.56	0.55	0.67	0.53	0.64	0.58	0.58	0.58	0.86	0.88	0.86	0.89	0.89	0.85	0.86	0.84	0.83	0.87
A. caviae	*A. caviae*	142	BQVL01	0.55	0.54	0.56	0.55	0.55	0.55	0.65	0.53	0.62	0.57	0.57	0.56	0.86	0.86	0.86	0.83	0.83	0.85	0.85	0.84	0.83	0.86
A. caviae	*A. caviae*	148	BQVO01	0.56	0.55	0.57	0.56	0.56	0.56	0.67	0.54	0.64	0.59	0.59	0.58	0.90	0.90	0.90	0.87	0.87	0.87	0.90	0.87	0.85	0.88
A. caviae	*A. caviae*	149	BPNC01	0.55	0.55	0.57	0.55	0.56	0.55	0.66	0.54	0.63	0.58	0.57	0.57	0.88	0.88	0.88	0.87	0.85	0.87	0.87	0.86	0.84	0.88
A. caviae	*A. caviae*	151	BPNT01	0.56	0.54	0.57	0.55	0.56	0.55	0.67	0.54	0.63	0.59	0.59	0.57	0.90	0.90	0.90	0.86	0.87	0.89	0.89	0.88	0.85	0.88
A. caviae	*A. caviae*	152	JAEHIL01	0.57	0.56	0.58	0.57	0.57	0.57	0.69	0.55	0.65	0.60	0.60	0.59	0.89	0.91	0.89	0.88	0.88	0.88	0.89	0.88	0.85	0.90
A. caviae	*A. caviae*	153	CAAKNG01	0.56	0.55	0.57	0.57	0.57	0.57	0.68	0.55	0.65	0.60	0.60	0.59	0.90	0.92	0.90	0.89	0.89	0.88	0.91	0.88	0.86	0.90
A. caviae	*A. caviae*	158	JAEHIC01	0.56	0.55	0.57	0.56	0.56	0.55	0.68	0.54	0.64	0.59	0.59	0.58	0.88	0.89	0.88	0.87	0.87	0.90	0.88	0.87	0.84	0.88
A. caviae	*A. caviae*	160	JAEHHN01	0.55	0.55	0.57	0.56	0.56	0.56	0.67	0.54	0.64	0.59	0.59	0.58	0.88	0.90	0.88	0.87	0.87	0.87	0.87	0.86	0.85	0.88
A. caviae	*A. caviae*	161	BPNN01	0.54	0.51	0.54	0.52	0.53	0.52	0.63	0.50	0.60	0.56	0.56	0.55	0.82	0.83	0.82	0.82	0.82	0.83	0.82	0.82	0.82	0.83
A. caviae	*A. caviae*	165	BQUY01	0.56	0.54	0.57	0.56	0.56	0.55	0.66	0.54	0.63	0.58	0.58	0.58	0.87	0.88	0.87	0.85	0.85	0.89	0.87	0.86	0.83	0.87
A. caviae	*A. caviae*	171	UETO01	0.57	0.55	0.58	0.56	0.56	0.56	0.67	0.55	0.64	0.60	0.60	0.58	0.90	0.90	0.90	0.86	0.87	0.89	0.89	0.88	0.85	0.89
A. caviae	*A. caviae*	176	JAEHIM01	0.56	0.55	0.57	0.56	0.56	0.56	0.67	0.55	0.64	0.59	0.59	0.58	0.88	0.89	0.88	0.87	0.87	0.87	0.88	0.86	0.85	0.89
A. caviae	*A. caviae*	178	CABMOA01	0.55	0.55	0.56	0.56	0.56	0.56	0.69	0.54	0.65	0.60	0.59	0.59	0.87	0.89	0.87	0.90	1.00	0.87	0.88	0.86	0.83	0.88
A. caviae	*A. caviae*	179	JAGDEV01	0.54	0.55	0.56	0.56	0.56	0.56	0.68	0.54	0.64	0.58	0.58	0.58	0.87	0.89	0.87	0.90	0.91	0.86	0.88	0.85	0.83	0.87
A. caviae	*A. caviae*	180	JAEHIE01	0.55	0.55	0.57	0.56	0.56	0.56	0.69	0.54	0.65	0.60	0.60	0.59	0.87	0.90	0.87	0.91	0.92	0.87	0.88	0.86	0.83	0.88
A. caviae	*A. caviae*	181	BPOE01	0.56	0.54	0.57	0.56	0.56	0.55	0.67	0.54	0.63	0.59	0.59	0.57	0.91	0.90	0.91	0.86	0.87	0.89	0.89	0.88	0.85	0.88
A. caviae	*A. caviae*	183	JAEHHY01	0.55	0.55	0.57	0.56	0.56	0.56	0.69	0.54	0.65	0.60	0.60	0.59	0.87	0.89	0.87	0.91	0.91	0.89	0.87	0.86	0.84	0.89
A. caviae	*A. caviae*	187	BPOR01	0.55	0.54	0.56	0.55	0.55	0.54	0.66	0.53	0.62	0.58	0.58	0.57	0.88	0.90	0.88	0.86	0.86	0.86	0.89	0.86	0.84	0.87
A. caviae	*A. caviae*	189	PUTR01	0.56	0.54	0.57	0.56	0.56	0.55	0.66	0.54	0.63	0.59	0.58	0.58	0.88	0.89	0.88	0.85	0.86	0.88	0.88	0.87	0.86	0.88
A. caviae	*A. caviae*	190	JAGDEX01	0.55	0.56	0.56	0.57	0.56	0.56	0.68	0.54	0.64	0.59	0.59	0.59	0.87	0.89	0.87	0.91	0.91	0.86	0.87	0.85	0.83	0.88
A. caviae	*A. caviae*	207	BPNA01	0.56	0.55	0.56	0.56	0.56	0.55	0.66	0.54	0.64	0.58	0.58	0.57	0.89	0.89	0.89	0.87	0.86	0.86	0.88	0.85	0.85	0.88
A. caviae	*A. caviae*	212	BPOL01	0.55	0.53	0.56	0.55	0.54	0.54	0.65	0.52	0.62	0.57	0.57	0.57	0.83	0.85	0.83	0.85	0.85	0.85	0.84	0.83	0.83	0.84
A. caviae	*A. caviae*	219	JAEHIJ01	0.56	0.55	0.57	0.56	0.56	0.55	0.67	0.54	0.63	0.59	0.59	0.58	0.90	0.91	0.90	0.87	0.86	0.88	0.90	0.87	0.85	0.89
A. caviae	*A. caviae*	220	BPNK01	0.56	0.54	0.56	0.54	0.55	0.54	0.64	0.52	0.62	0.57	0.57	0.56	0.86	0.86	0.86	0.83	0.82	0.84	0.85	0.84	0.83	0.86
A. caviae	*A. caviae*	224	BPNY01	0.54	0.53	0.55	0.54	0.54	0.53	0.64	0.51	0.61	0.57	0.56	0.56	0.83	0.85	0.83	0.85	0.85	0.84	0.83	0.82	0.82	0.84
A. caviae	*A. caviae*	228	JAEHJL01	0.56	0.56	0.58	0.57	0.58	0.57	0.69	0.55	0.66	0.60	0.60	0.60	0.89	0.91	0.89	0.89	0.89	0.89	0.90	0.87	0.85	0.90
A. caviae	*A. caviae*	230	BQVX01	0.55	0.54	0.56	0.55	0.55	0.55	0.65	0.53	0.62	0.57	0.57	0.56	0.86	0.86	0.86	0.83	0.83	0.85	0.85	0.84	0.83	0.86
A. caviae	*A. caviae*	235	BPOH01	0.54	0.53	0.55	0.55	0.54	0.54	0.65	0.52	0.61	0.57	0.57	0.57	0.83	0.85	0.83	0.85	0.85	0.85	0.84	0.83	0.81	0.84
A. caviae	*A. caviae*	252	JAAALU01	0.55	0.54	0.57	0.55	0.56	0.55	0.67	0.54	0.63	0.58	0.58	0.57	0.87	0.89	0.87	0.86	0.87	0.86	0.87	0.86	0.85	0.88
A. caviae	*A. caviae*	254	CDBK01	0.54	0.55	0.56	0.56	0.56	0.56	0.69	0.54	0.64	0.58	0.58	0.58	0.86	0.88	0.86	0.90	0.90	0.85	0.86	0.85	0.83	0.87
A. caviae	*A. caviae*	259	JAAALX01	0.56	0.56	0.57	0.57	0.57	0.57	0.68	0.55	0.65	0.60	0.60	0.59	0.88	0.91	0.88	0.89	0.89	0.88	0.89	0.87	0.86	0.89
A. caviae	*A. caviae*	272	QKOU01	0.55	0.54	0.56	0.56	0.55	0.55	0.66	0.53	0.63	0.57	0.57	0.58	0.84	0.86	0.84	0.87	0.87	0.84	0.85	0.83	0.83	0.85
A. caviae	*A. caviae*	278	QKPH01	0.52	0.52	0.53	0.52	0.52	0.52	0.64	0.50	0.60	0.55	0.54	0.54	0.81	0.83	0.81	0.82	0.82	0.82	0.82	0.81	0.78	0.82

[illegible]

Supplementary Table 4. Misidentified genomes and their correct taxonomic affiliation based on phylogenetic analysis and OrthoANI

Acc.no	Serial no.	NCBI species name (originally designated species)	Taxonomic affiliation based on phylogenetic tree (presumed species)	OrthoANI value against type strain of presumed species	Re-identified species name
JEMK01	116	*A. hydrophila*	*A. dhakensis*	97.176	*A. dhakensis*
ANPN01	177	*A. hydrophila*	*A. dhakensis*	97.3004	*A. dhakensis*
JWJR01	184	*A. jandaei*	*A. dhakensis*	97.2878	*A. dhakensis*
AOBO01	203	*A. hydrophila*	*A. dhakensis*	97.2657	*A. dhakensis*
AOBP01	204	*A. hydrophila*	*A. dhakensis*	97.2837	*A. dhakensis*
CP006579.1	19	*A. hydrophila*	*A. rivipollensis*	95.6574	*A. rivipollensis*
CP050994.1	35	*A. hydrophila*	*A. rivipollensis*	97.2004	*A. rivipollensis*
CP038441.1	47	*A. media*	*A. rivipollensis*	96.8833	*A. rivipollensis*
CP061477.1	51	*A. media*	*A. rivipollensis*	96.9025	*A. rivipollensis*
CALUBH01	85	*A. media*	*A. rivipollensis*	97.2402	*A. rivipollensis*
UETL01	261	*A. media*	*A. rivipollensis*	97.1698	*A. rivipollensis*
AP022038.1	66	*A. veronii*	*A. allosaccharophila*	96.2179	*A. allosaccharophila*
AP022264.1	67	*A. veronii*	*A. allosaccharophila*	96.3007	*A. allosaccharophila*
AP022290.1	68	*A. veronii*	*A. allosaccharophila*	96.2183	*A. allosaccharophila*
BQUX01	144	*A. caviae*	*A. hydrophila*	96.8988	*A. hydrophila*
OU015320.1	1	*A. australiensis*	*A. media*	97.1512	*A. media*
AGWU01	279	*A. veronii*	No closely located species	-	undetermined
